# A lesion-derived brain network of somatic symptoms for transdiagnostic individual application

**DOI:** 10.1186/s12916-026-05044-y

**Published:** 2026-07-02

**Authors:** Yue Yu, Wenqiang Xu, Yang Ji, Li Qi, Yue Wu, Yuanyuan Guo, Qiang Hua, Lihong Wang, Rui Qian, Tongjian Bai, Qianqian Li, Feng Geng, Yuhui Wan, Kai Wang, Gong-Jun Ji, Yanghua Tian

**Affiliations:** 1 Department of Neurology, the First Affiliated Hospital of Anhui Medical University, 218 Jixi Rd, Hefei, 230022 China; 2 School of Mental Health and Psychological Sciences, Anhui Medical University, 81 Meishan Rd, Hefei, 230032 China; 3 Department of Neurology, the Second Affiliated Hospital of Anhui Medical University, Hefei, 230601 China; 4 Department of Psychology and Sleep Medicine, The Second Affiliated Hospital of Anhui Medical University, Hefei, 230601 China; 5 Center for Big Data and Population Health of IHM, School of Public Health, Anhui Medical University, Hefei, 230032 China; 6 Anhui Institute of Translational Medicine, Hefei, 230032 China; 7 Anhui Province Key Laboratory of Cognition and Neuropsychiatric Disorders, Hefei, 230032 China; 8 Collaborative Innovation Center of Neuropsychiatric Disorders and Mental Health, Hefei, 230032 China; 9 Institute of Artificial Intelligence, Hefei Comprehensive National Science Center, Hefei, 230032 China; 10 Department of Neurology, Division of Life Sciences and Medicine, The First Affiliated Hospital of USTC, University of Science and Technology of China, Hefei, 230001 Anhui China

**Keywords:** Somatic symptom, Lesion network mapping, Interoception, Brain stimulation, Normative model, Functional connectivity

## Abstract

**Background:**

Somatic symptoms is an umbrella term that describes distressing somatic complaints occurring across a wide spectrum of diseases. However, their underlying neural mechanisms remain poorly understood. Elucidating their mechanisms could therefore benefit patients with diverse conditions.

**Methods:**

Using a recently validated lesion network mapping method and a large-scale healthy connectome database (*n* = 652, recruited in Anhui Province, China), we identified a somatic network from brain lesions causing somatic symptoms. The lesion-derived network was validated using independent multimodal neuroimaging signatures of somatic symptoms and interoceptive processing. It was further characterized by transcriptomic, neurochemical, and cognitive meta-analytic mapping. We further assessed the therapeutic potential of the somatic network by quantifying its spatial convergence with empirically validated neuromodulation targets. Finally, to determine whether the group-level network can predict personalized symptom severity across diagnoses, we built a normative model of gray matter volume using Gaussian process regression based on 1,342 healthy controls from Anhui. This model was then used to generate personalized atrophy maps in 399 local somatic patients with anxiety, depression, schizophrenia and bipolar disorder. We then assessed whether individual atrophy volume within the somatic network was associated with somatic symptom severity.

**Results:**

We found that 21 heterogeneous lesions associated somatic symptoms occurred in many different brain locations but were characterized by a common brain network, with the hub region of the right insula and putamen. The somatic network demonstrated strong spatial alignment with multimodal somatic imaging abnormalities from 66 independent studies and interoceptive processing circuits from 69 task-fMRI studies. 11 effective brain stimulation targets were colocalization with the somatic network. In individual transdiagnostic patients, greater atrophy volume within the somatic network was significantly correlated with somatic symptom severity (*r* = 0.182, *p* = 0.004), but not with anxiety or depression symptoms.

**Conclusions:**

These convergent findings establish a unified somatic network framework, advancing both mechanistic understanding and precision medicine applications.

**Supplementary Information:**

The online version contains supplementary material available at https://doi.org/10.1186/s12916-026-05044-y.

## Background

Somatic symptoms is an umbrella term used to describe persistent distressing somatic complaints, regardless of cause, often present on most days for several months [1]. Somatic symptoms occur in diverse clinical contexts including functional somatic disorders, mental disorders, and some undiagnosed conditions [2]. Therefore, elucidating the mechanisms of somatic symptoms holds immense potential to benefit patients across a wide spectrum of diseases.

Although multimodal neuroimaging studies have identified structural and functional brain impairments associated with somatic symptoms [3–5], most have relied on small sample sizes and cross-section comparisons, not only undermining statistical robustness but also limiting the ability to infer causal relationships [6, 7]. Lesion analyses emerged as a promising approach to causally link specific brain regions to neurological symptoms [8]. However, traditional lesion analysis only considered local changes while neglecting the distant effect of lesions mediated by functional connectivity (FC). Thus, a novel method, lesion network mapping (LNM), has been proposed and validated to identify the common network of multiple lesions causing a similar clinical symptom [8, 9]. LNM has been applied to a variety of neuropsychiatric symptoms such as auditory hallucinations, aphasia, pain, and disorders of volition [9–12]. As somatic symptoms has been recognized resulting from brain network dysfunction [1], LNM would represents a promising approach to explore its underlying mechanisms.

The network identified by LNM reflects a group-level finding. Determining whether and how this population-based finding can be applied to resolve individual problems is of great importance for its clinical translation. Normative modeling can complement the LNM analysis through individual-level inferences [13, 14]. Normative modeling is a statistical framework that connects behavioral, demographic, or clinical characteristics with quantitative brain phenotype, such as gray matter volume (GMV) [13, 15]. The model can establish the normative range of variants for comparison with new individuals. By fitting the model to data from various brain regions, a personalized deviation map can be generated to quantify the extent to which each new individual deviates from the population norms [13]. We hypothesize that integrating normative modeling with LNM may enable individualized localization of symptom-specific neural substrates, thereby validating the clinical translatability of the group-level somatic network.

**Fig. 1:**
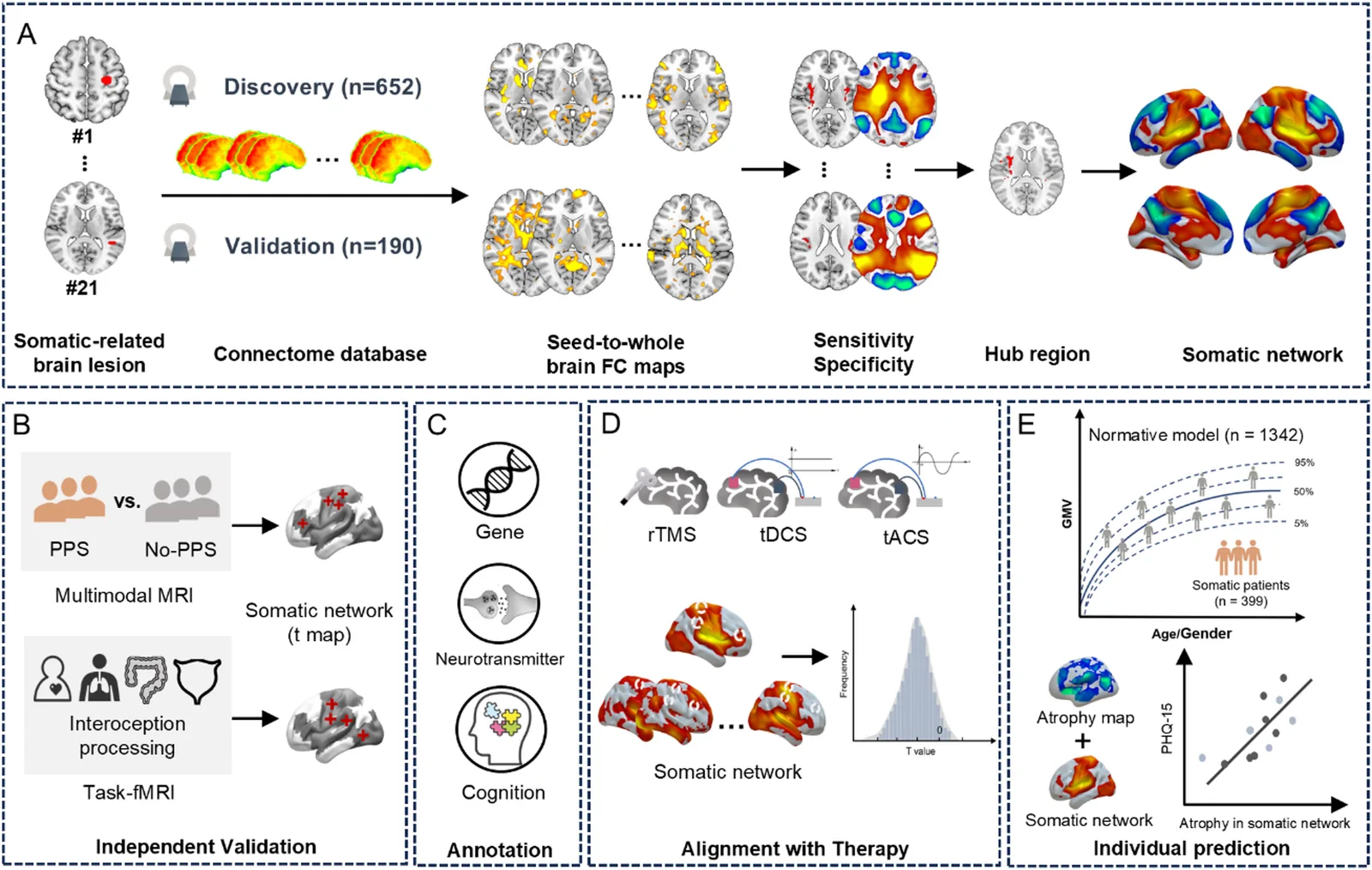
Study design and analysis workflow. A Somatic network construction: Brain lesions causing somatic symptoms were used as ROIs for computing functional connectivity in both discovery and validation connectome databases to construct a somatic network; B Independent validation data were from studies of somatic-related neuroimaging abnormalities and task-fMRI of interoceptive processing. We tested whether the somatic network was spatially aligned with regions (red dots) reported in the validation studies. C The somatic network was annotated with symptom related genes, neurotransmitters, and cognitive processing. D We tested whether the somatic network was co-localized with the effective brain stimulation (rTMS, tDCS and tACS) targets (white circles); E We combined the somatic network and a normative model to show the potential of somatic network in individual application. This model was established from structural imaging of 1342 HCs. We predicted that the individual atrophy volume in the somatic network is significantly correlated with somatic symptom severity (n = 399). Abbreviations: FC, functional connectivity; ROI, region of interests; rTMS, repetitive transcranial magnetic stimulation; tACS, transcranial alternating current stimulation; tDCS, transcranial direct current stimulation; task-fMRI, task-state magnetic resonance

In this study, we first identified the common network of brain lesions causing somatic symptoms using LNM. This somatic network was then (1) validated through neuroimaging signatures of somatic symptoms and interoceptive processing circuits; (2) annotated by a variety of genes, neurotransmitters, and cognitions; (3) and tested for therapeutic implication using a variety of brain stimulation data. In particular, we leveraged a cohort of 1342 healthy controls (HCs) to establish a normative model, which was then utilized to rigorously evaluate the somatic network’s efficacy in identifying personalized and symptom-specific brain atrophy. A schematic overview of the study design and analysis workflow is provided in Fig. 1.

## Methods

This study was carried out according to the Declaration of Helsinki, approved by the institutional review board of the Anhui Medical University. All participants provided written informed consent after receiving a full explanation of study procedures and goals.

### Case selection and lesion tracing

To identify brain lesions causing somatic symptoms, we performed a systematic literature search on PubMed, Web of Science and Embase databases following PRISMA guidelines. The search string combined terms describing somatic symptoms with terms describing lesions and neuroimaging. The inclusion criteria and search strategy are detailed in Additional file 1: Method S1 [16]. This protocol was registered on PROSPERO (https://www.crd.york.ac.uk/PROSPERO/; registration number: CRD42024623688).

**Fig. 2:**
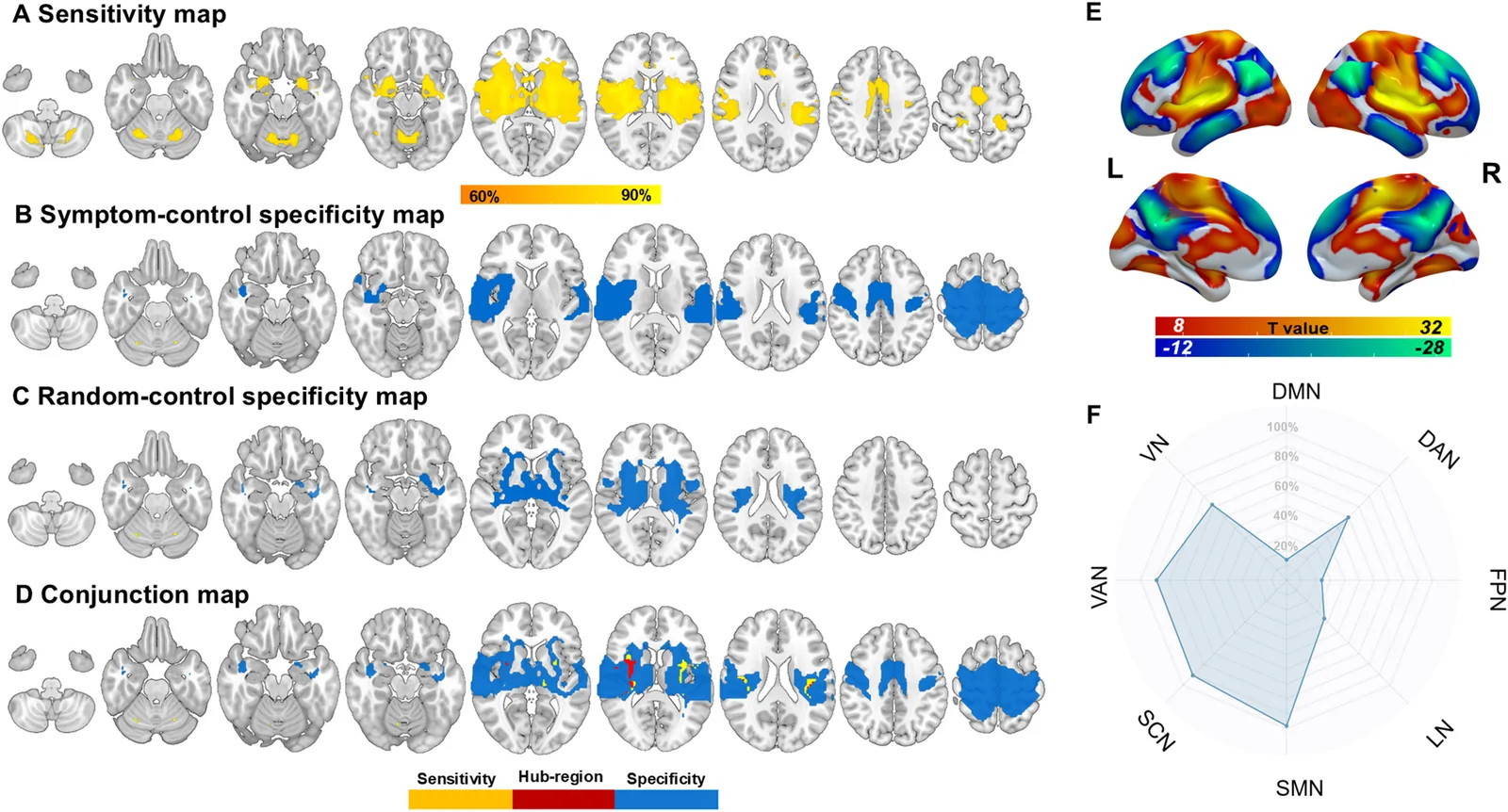
Lesion network mapping. **A** Sensitivity map: the networks generated from local lesions were binarized (*P*_FWE_ < 0.01) to get the sensitivity map that showing the common connectivity among cases. **B** Symptom-control specificity map: the networks of somatic related lesions were compared to a group of networks of non-somatic lesions (*P*_FWE_ < 0.05). **C** Random-control specificity map: the networks of somatic symptoms related lesions were compared to random networks (*P*_FWE_ < 0.05). **D** Conjunction map: The region that were both sensitive (threshold, 80%) and specific (*P*_FWE_ < 0.05) to somatic symptoms. The hub region of somatic symptoms is located in the right insula and putamen. **E** Somatic network: we examined the whole-brain functional connectivity of hub region in normative connectome to generate a somatic network. **F** Association of somatic network with classical brain networks canonical brain networks. Abbreviations: DMN, default mode network; DAN, dorsal attention network; FPN, Fronto-Parietal Network; LN, limbic network; SMN, sensorimotor network; SCN, salience network; VAN, ventral attention network; VN, visual network

Lesion locations were traced in the two-dimensional (2D) planes depicted in the published article, using neuroanatomical landmarks to ensure accurate mapping onto a template brain. All brain lesions were traced from structural three-dimensional (3D) images in 3D Slicer (v5.0.2, https://www.slicer.org/) [17] and registered to the normalized MNI152 T1 2 mm brain template (http://fsl.fmrib.ox.ac.uk/fsldownloads/) [9]. (Fig. 2A) To approximate the natural 3D contours of lesions, 2D lesions in the figures were extended by 2 mm perpendicular to the display plane, ensuring reproducibility and minimizing false overlap while preserving true lesion overlap. For comparison, a more liberal 4 mm extension was also analyzed. Lesion tracing was conducted by two researchers (YY and LQ) independently, with accuracy verification by GJ.

### Human connectome databases

To identify the functional connections of these lesion locations, we used two human connectome databases derived from resting-state fMRI data (rs-fMRI) of adults for LNM analysis: discovery database of 652 HCs and validation database of 190 patients with somatic symptoms. We collected resting-state functional MRI (rs-fMRI) and structural MRI of 652 healthy individuals (mean age ± SD = 22.9 ± 5.53 years; 334 females) from the community, and 190 somatic patients (median age = 35 years; IQR: 26–48; 130 females) from Anhui Mental Health Center (AMHC) or The First Affiliated Hospital of Anhui Medical University (AHMU). The inclusion/exclusion criteria and demographic characteristics are detailed in Additional file 1: Methods S2 [18] and Tables S1. Rs-fMRI data were obtained from the University of Science and Technology of China (USTC) with a 3-T scanner (Discovery 750; GE Healthcare, Milwaukee, Wisconsin, USA). The information of MRI databases and processing steps are detailed in the Additional file 1: Method S3 [19].

### Lesion network mapping (LNM)

LNM—a well-established, effective approach [9]—was employed to determine whether lesions causing somatic symptoms are mapped to a specific brain network. This approach involves sensitivity, specificity, conjunction analyses, and network mapping [20–23]. Among these, local-to-network mapping is a critical procedure that computes functional connectivity between each lesion location and all other brain voxels to generate a network (Fig. 1A). The term “local” encompasses not only specific lesion locations, but also whole-brain maps weighted by alternative metrics, such as the density of a specific neurotransmitter or the expression level of a particular gene. Local-to-network mapping involved two steps. First, the average (or weighted average) signal of the local areas was correlated with the signal of each brain voxel using Pearson’s correlation based on the functional connectome database. Next, a one-sample t-test with voxel-wise family-wise error (FWE) corrected was conducted to identify voxels significantly connected to the local areas (𝑃_*FWE*_ < 0.01). The resulting t-map was defined as the brain network of the local areas.

### Sensitivity analysis

The networks functionally connected to each lesion location were identified through local-to-network mapping based on the human connectome database. These connectivity maps were then binarized (*t* > 5.1; voxel-wised 𝑃_*FWE*_ < 0.01) and overlapped (i.e., summed and divided by the number of studies) to identify common connections across all lesions. The sensitivity map was defined by applying a threshold of 0.80.

### Specificity analysis

To identify somatic-specific regions in the sensitivity map, we conducted a specificity analysis between the networks derived from 21 somatic cases and two distinct control groups: (1) networks generated from 97 non-somatic lesions by local-to-network mapping. The inclusion criteria of non-somatic lesions are detailed in Additional file 1: Method S4 [24, 25]. (2) random networks. The random networks were calculated by local‒to‒network mapping using the randomly generated seed points, which with the same voxel count as the brain lesions causing somatic symptoms. These two control groups were compared to the 21 somatic networks using 5,000 permutation tests, resulting in symptom-controlled and random-controlled specificity maps, respectively. A cluster-level *P*_*FWE*_ < 0.05 (with the cluster-forming threshold at voxel-level *p* < 0.001) was considered statistically.

### Conjunction analysis

To identify the hub region of somatic symptoms, we performed a conjunction analysis to identify regions that were both sensitive (connected to > 80% of the sensitive map) and specific (voxels surviving both specificity analyses) to somatic symptoms. The identified regions were considered the hub regions of somatic symptoms.

### Network mapping

Using the hub regions as seeds, we derived a brain network via local-to-network mapping (a t-map with voxel-wised *P*_*FWE*_ < 0.01). The network functionally connected to the hub regions was defined as the somatic network, encompassing lesion locations specifically causing somatic symptoms.

### Robustness analysis

To validate the robustness of the identified somatic network, we performed the following three control analyses:

1. Database independence: to ensure that the somatic network was not influenced by databases, we repeated LNM analysis using an independent validation database of patients with somatic symptoms;
2. Lesion size effects: to ensure that the somatic network was independent of lesion mask size, we repeated the analysis using a more permissive 4 mm lesion extension during tracing [9];
3. Hub region effects: To ensure that the somatic network was not driven by the hub region size, we repeated the analysis using an 85% overlap of the sensitivity map.

Voxel-wise spatial correlations between somatic networks were examined to quantitatively assess the effects of dataset selection, lesion mask size, and hub region size, respectively.

### Association with canonical brain networks

To enhance interpretability, we investigated the spatial relationships between the somatic network and eight well-established canonical brain networks [26]. The spatial association was quantified by calculating the proportion of overlapping voxels between the somatic network and each canonical network relative to the total number of voxels within the corresponding canonical network.

### Multi-validation analyses

#### Validation of multimodal imaging findings in somatic symptoms

We identified peak coordinates of structural (GMV) or functional imaging abnormalities of somatic symptoms from published studies through a systematic literature search. The inclusion criteria are detailed in Additional file 1: Method S5 [27, 28]. The functional markers comprised amplitudes of low-frequency fluctuations (ALFF), regional homogeneity (ReHo), and task-based functional MRI (task-fMRI). Coordinates reported in Talairach space were converted to Montreal Neurological Institute (MNI) space using GingerALE (version 3.0.2, www.brainmap.org). We created a 3 mm-radius sphere centered at each peak coordinate. For studies reporting multiple coordinates, spheres from the same study were merged into study-level regions of interest (ROIs). We then extracted the average t-values of these ROIs within the somatic network and performed a one-sample t-test to assess its functional connectivity strength with the hub region. Furthermore, we implemented two control conditions: (1) 58 non-somatic control ROIs derived from independent studies and (2) 66 randomly generated ROIs matched for voxel count. We then extracted average t-values for control ROIs and conducted ANOVA to compare their difference with the values of somatic-related ROIs, post hoc pairwise comparisons were conducted using Fisher’s Least Significant Difference (LSD) test.

Subsequently, we classified the retrieved imaging indicators into structural and functional categories, and somatic symptoms into four subtypes: pain, gastrointestinal symptoms, anxiety and depression with somatic symptoms, and general somatic symptoms. The above analyses were then repeated to validate the robustness of the network.

#### Validation of interoceptive processing

Interoceptive dysfunction has been implicated as an important psychological mechanism of somatic symptoms [29]. To validate the FC between interoceptive processing circuit and the somatic network, we identified task-fMRI studies on interoceptive processing in HCs from a recent meta-analysis [30]. Voxel coordinates of task-induced activation were extracted, and 3 mm-radius spherical ROIs were generated around peak coordinates. Multiple coordinates from the same study were merged into a study-level ROI. The average t-values of interoceptive ROIs within the somatic network were extracted and assessed using a one-sample t-test. Additionally, ANOVA was performed to compare t-values of interoceptive ROIs with those of non-interoceptive and random ROIs, post hoc pairwise comparisons were conducted using LSD test. The non-interoceptive ROIs were identified from recently published meta-analysis [31–33].

### Network annotations

#### Genes

We adopted 214 somatic symptoms risk genes from the largest GWAS. In line with our previous studies, gene expression maps were constructed using the abagen toolbox (v.0.1.3, https://github.com/rmarkello/abagen) based on data from the Allen Human Brain Atlas (AHBA) (http://human.brain-map.org), which includes 3,702 spatially distinct samples from six post-mortem brains (age: 42.50 ± 13.38 years, male/female = 5/1) [34, 35]. Following probe reannotation, low-intensity probes and duplicates were filtered, retaining the most stable probes for each gene. Normalized gene expression values were computed for 160 Human Connectome Project-Multi-Modal Parcellation (HCP-MMH) atlas [36] regions, 9 cerebellum, and 4 subcortical ROIs. Owing to missing data in the right hemisphere of two AHBA brains, only left hemisphere ROIs were included. Expression maps were then transformed into brain networks via local-to-network mapping. Gene enrichment analysis was conducted by Metascape (https://metascape.org/) [37]. The obtained biological processes were corrected by False Discovery Rate (FDR) correction (*P*_*FDR*_ < 0.05). The processes of genes selection and annotation were detailed in Additional file 1: Methods S6 [18, 34–36, 38–52].

#### Neurotransmitters

Neurotransmitter density maps were obtained from previous positron emission tomography studies of healthy individuals, including dopamine receptors (D1 receptor, D1R [53] and D2 receptor, D2R [54]), serotonin receptors (5-hydroxytryptamine 1 A receptor, 5-HT1AR [55]; 5-hydroxytryptamine 1B receptor, 5-HT1BR [56]; 5-hydroxytryptamine 2 A receptor, 5-HT2AR [56]; 5-hydroxytryptamine 4 receptor, 5-HT4R [56]), a dopamine transporter (DAT [57]), a serotonin transporter (5-hydroxytryptamine transporter, 5-HTT [56]), fluorodopa (F-DOPA), γ-aminobutyric acid type A receptor (GABAAR [57]), norepinephrine transporter (NAT [58]), vesicular acetylcholine transporter (VAChT [54]) and metabotropic glutamate receptor (mGluR [54]). The 13 distribution maps of neurotransmitter receptors and transporters were transformed into brain networks also through local-to-network mapping.

#### Cognition

The neural correlates of different cognitive processes have been reported by abundant task-fMRI studies. Neurosynth, a meta-analytic tool (https://github.com/neurosynth/neurosynth), synthesized findings from more than 15,000 studies by searching for high-frequency keywords using volumetric association test maps [59]. We focused primarily on cognitive function and limited the terms of interest to 123 cognitive or behavioral terms selected from the Cognitive Atlas—a public ontology of cognitive science [60]. The coordinates reported by Neurosynth in the Schaefer-100 atlas [54, 61] were transformed into brain networks also through local-to-network mapping.

#### Null model for spatial correlation

The spatial correlation between networks was tested against a null model to correct for spatial autocorrelation using Moran’s test [62] implemented in the Neuromaps toolbox [63] (https://github.com/netneurolab/neuromaps). The analysis encompassed 398 brain regions, comprising 360 cortical areas from the HCP-MMP atlas [36] and 38 cerebellar and subcortical structures derived from an automated anatomical labeling template [52]. A null model was generated by performing 5,000 spatial permutations, and the empirical p-value was calculated as the proportion of permuted results exceeding the observed correlation strength. For analyses involving multiple comparisons, statistical significance was further adjusted using the Benjamini–Hochberg FDR correction.

### Alignment with therapeutic targets

To evaluate whether the defined somatic network can serve as a potential target network for clinical treatment, we analyzed its connectivity with empirically validated neuromodulation targets. We conducted a literature search to identify brain stimulations that improve somatic symptoms, including transcranial magnetic stimulation (TMS), transcranial direct current stimulation (tDCS), and transcranial alternating current stimulation (tACS). Studies that provided MNI or Talairach coordinates of stimulation targets or clear anatomical depictions of stimulation sites were selected. The inclusion criteria are detailed in Additional file 1: Method S7. Coordinates reported in Talairach space were converted to MNI space. We created a 3 mm-radius sphere centered at each target coordinate and tested whether the targets were aligned with the somatic network by a permutation test. Specifically, we randomly redistributed the hub regions of the somatic network 2,000 times, generating 2,000 random networks through local-to-network mapping. We extracted the average value of target spheres independently in these random networks. A *p* value was defined as the proportion of 2,000 random results with values greater than that of the somatic network.

### Normative model and individual atrophy analysis

To identify the clinical predictive value of the somatic network, we employed normative model to assess correlation between somatic network-specific individual atrophy and severity of somatic symptom. Structural MRI data of 1342 HCs (30.61 ± 0.45 years; 713 females) from community were collected at the USTC with a 3-T scanner to construct a normative model. In addition, 399 patients with somatic symptoms (30.77 ± 0.64 years; 271 females) diagnosed with anxiety, depression, schizophrenia and bipolar disorder, were recruited from AMHC or AHMU. Their structural MRI data were also collected at the USTC to assessed individual atrophy volume. The inclusion/exclusion criteria and demographic characteristics are detailed in Additional file 1: Method S8 and Tables S13.

After controlling image quality, the voxel-wise estimates of GMV for each participant were aggregated into regional estimates based on well-validated brain parcellations, comprising 1,000 cortical [64] and 32 subcortical regions [65]. Then, for each of the 1,032 brain regions, a normative model of GMV was estimated as a function of age and sex by using gaussian process regression (GPR) in 1342 HCs [66, 67]. Normative modeling was performed using the Predictive Clinical Neuroscience toolkit package (PCNtoolkit, version 0.30, https://pcntoolkit.readthedocs.io/en/latest/). To assess model generalizability, GPR models were trained on 80% of HCs using age and sex as covariates, while the remaining 20% were withheld for testing. We assessed model fit for each brain region by evaluating three performance metrics: explained variance, overall standardized mean squared error, and mean squared log-loss [68]. The processes of GMV estimation, normative modeling and model evaluation are detailed in Additional file 1: Method S8 [8, 67, 69–74].

The established normative models were subsequently applied to 399 patients with somatic symptoms. For each patient, we quantified the extent to which regional GMV estimates deviated from normative model predictions as *z *values. For a given somatic patient *i*, the deviation *z* value of a brain region *j* was calculated as follows:

1 $$z_{ij} = \frac{y_{ij} - \hat{y}_{ij}}{\sqrt{\sigma_{ij}^2 + \sigma_{nj}^2}}$$

where ${\it{y}_{ij}}$ is the observed GMV, $\hat{y}_{ij}$ is the predictive GMV, $\sigma_{ij}$is the predictive uncertainty, and $\sigma_{nj}$is the variance learned from the normative distribution. We established the threshold using *z* < -1.96 (i.e., *p* < 0.05) to further define individual-level extreme negative deviations, which were quantified as GMV atrophy maps of each somatic patient.    

We hypothesized that the somatic network could be used to identify somatic-specific atrophy, which is critical to symptom-specific neuromodulation. In order to verify this hypothesis, we calculated the volume of atrophy (total voxel number) within the somatic network for each patient and examined its correlation with Patient Health Questionnaire-15 (PHQ-15) scores. To evaluate the specificity of the somatic network in predicting somatic symptoms, we also examined the correlation between the atrophic volume within the somatic network and other clinical symptoms, including anxiety and depression.

## Results

### Systematic search and brain lesions

The systematical search revealed 21 cases of brain lesions from 12 studies, which were included in our analyses to derive a somatic network (Additional file 1: Table S2, Figure S1). These 21 cases primarily reflect somatoform disorders (*n* = 15), chronic pain (*n* = 6), palpitations (*n* = 4), or excessive somatic preoccupation (*n* = 17). The lesions scatter across a variety of brain areas including the cortex, brainstem, limbic structures, subcortical nuclei, and cerebellum (Additional file 1: Figure S2).

### Somatic network identified by LNM

The 21 heterogeneous brain lesions causing somatic symptoms were transformed into networks by employing a local-to-network mapping approach. Over 80% of lesions showed common connectivity to the bilateral insula, thalamus, putamen, and Rolandic operculum (sensitivity map, Fig. 2A), Notably, 90% showed common FC to the bilateral insula and left Rolandic operculum. Part of the common connectivity was specific to somatic symptoms compared to non-somatic symptoms and random networks, two-sample t-test with FEW corrected *P*_*FWE*_ < 0.05 (specificity maps, Fig. 2B–C). A conjunction analysis revealed that one cluster in the right insula and putamen (the hub region) was both sensitive (> 80% in the sensitivity map) and specific (*P*_*FWE*_ < 0.05 in both specificity maps) to somatic symptoms (conjunction map, Fig. 2D). The whole-brain connectivity map of the hub region delineated a distributed brain network encompassing lesion locations that cause somatic symptoms (hereafter, the somatic network) (Fig. 2E). The positive component of the Somatic network mainly overlaps with several canonical brain networks, specifically the sensorimotor network (SMN, 95.55%), subcortical network (SCN, 72.50%), and ventral attention network (VAN, 79.88%) (Fig. 2F).

### Robustness analysis

The lesion-derived somatic network demonstrated robustness across different methodological variations. First, a strong voxel-wise spatial correlation was observed between the networks derived from diseased and healthy connectome databases (*r* = 0.95; Additional file 1: Figure S4). Second, the network showed high reproducibility (*r* = 0.98; Additional file 1: Figure S5) when using more permissive 4 mm lesion margin extensions compared to 2 mm extensions. Finally, the network demonstrated robustness as applying an 85% threshold for the sensitivity map generated a highly consistent network topology (*r* = 0.99; Additional file 1: Figures S6–S7).

### Multi-validation analyses

#### Validation through imaging signatures of somatic symptoms

**Fig. 3:**
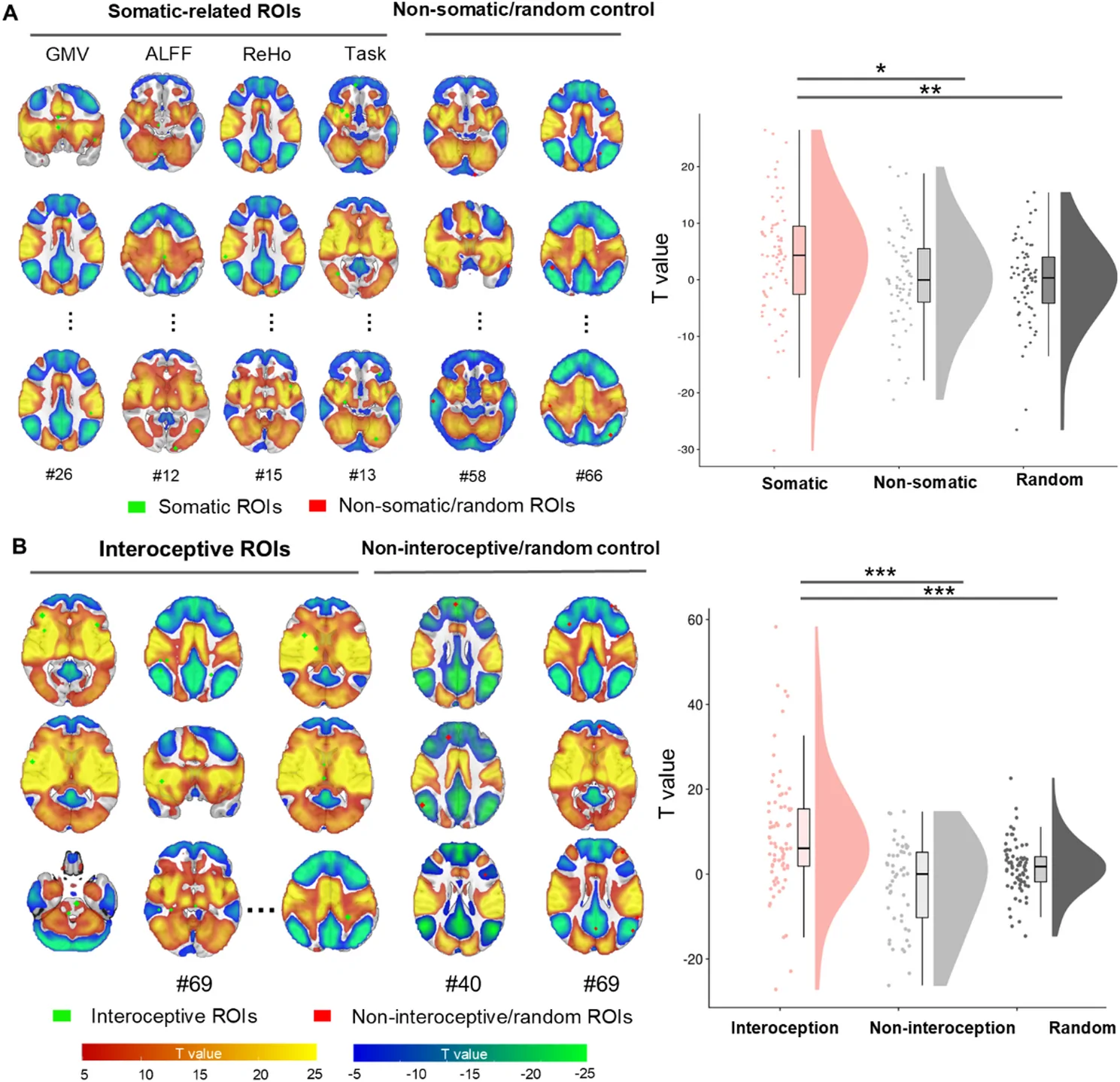
Multiple validation**. A** Validation of brain structural and functional imaging. We identified 66 somatic-related ROIs, 58 non-somatic ROIs and 66 random ROIs. somatic-related ROIs had significantly stronger functional connectivity with somatic network compared to both the non-somatic ROIs and random ROIs (*F* = 3.931, *p* = 0.021). **B** Validation of interoceptive processing. We identified 69 interoception ROIs, 40 non-interoception ROIs, and 69 random control ROIs. Interoception ROIs had significantly stronger functional connectivity with somatic network compared to both the non-interoception ROIs and random ROIs (*F* = 17.953, *p* < 0.0001). * *p* < 0.05; ** *p* < 0.01; *** *p* < 0.001

A total of 66 case-control studies investigating somatic-related imaging abnormalities were identified, comprising 2,721 somatic patients and 3,541 HCs. Specifically, there are 26 GMV studies (1,525 somatic patients and 2,326 HCs), 12 ALFF studies (433 somatic patients and 449 HCs), 15 ReHo studies (499 somatic patients and 498 HCs), and 13 task-fMRI studies (264 somatic patients and 268 HCs) (Additional file 1: Tables S4–S6). These 66 study-level ROIs revealed significant FC with the somatic network (one-sample t-test: *t* = 3.297, *p* = 0.002). Additionally, we implemented two control conditions: 58 non-somatic ROIs and 66 random ROIs (Additional file 1: Table S7). ANOVA revealed that FC of somatic-related ROIs differed significantly from two control ROIs with somatic network (*F* = 3.931, *p* = 0.021). Post hoc analysis revealed somatic-related ROIs exhibited significantly stronger FC with the somatic network compared to both non-somatic and random ROIs (*p* = 0.043; *p* = 0.008) (Fig. 3A). The results remained robust when we repeated the analyses by disease subtype and by imaging indicator. (Additional file 1: Figure S8, S9)

#### Validation through interoceptive processing circuits 

We identified 69 task-fMRI studies of interoceptive processing from a recent meta-analysis [30] (Additional file 1: Table S8). The interoceptive ROIs revealed significant FC with the somatic network (one-sample t-test: *t* = 5.414, *p* < 0.0001). Additionally, we identified 40 non-interoceptive ROIs and 69 random ROIs (Additional file 1: Table S9). ANOVA revealed that FC of interoceptive ROIs differed significantly from two control ROIs with somatic network (*F* = 17.953, *p* < 0.0001). The interoceptive ROIs exhibited stronger connectivity in the somatic network compared to both non-interoceptive and random ROIs (*p* < 0.0001; *p* < 0.0001) (Fig. 3B).

### Network annotations

#### Genetic and molecular mechanisms

**Fig. 4:**
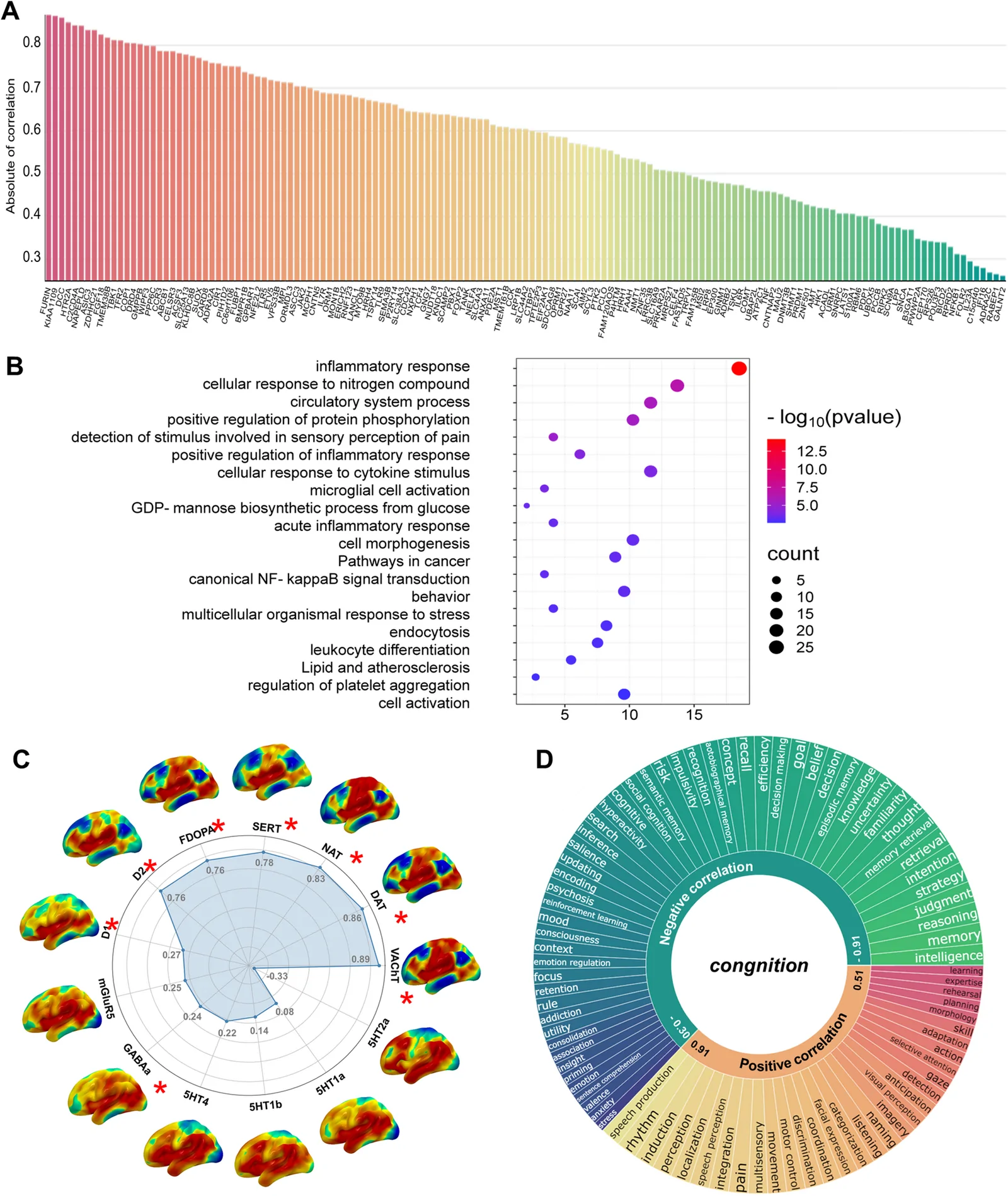
Annotation of the somatic network.** A** The somatic network showing a similar network pattern as 149 of the 216 risk genes (*P*_*FDR*_ < 0.05). **B **Enrichment analysis identified 20 significant terms (*P*_*FDR*_ < 0.05). Bubble size represents the number of genes in this term, and color represents enrichment significance (-log_10_ (*p* value)). **C** The somatic network shows significant spatial correlation with 8 neurotransmitter networks marked by red asterisks. The brain maps illustrating the network pattern of each neurotransmitter transporter/receptor. **D** The somatic network showing significantly positive correlation (warm color) and anti-correlation (cool color) with 31 and 53 cognitive networks, respectively

There were 147 gene networks that revealed significant spatial correlation with the somatic network (*P*_*Moran&FDR*_ < 0.05, Fig. 4A, Additional file 1: Table S10). Enrichment analysis further indicated that these genes were significantly enriched in several ontological terms such as “detection of stimulus involved in sensory perception of pain” and “inflammatory response” (*P*_*FDR*_ < 0.05, Fig. 4B). There were 8 neurotransmitter networks revealed significantly spatial correlation with the somatic network, including 4 dopamine transporter/receptor, 1 serotonin transporter, 1 gamma aminobutyric acid (GABA) subtype A receptor, 1 noradrenaline transporter, and 1 vesicular acetylcholine transporter (*P*_*Moran&FDR*_ < 0.05, Fig. 4C).

### Cognition relevance

The somatic network revealed a significantly positive correlation and an anti-correlation with 31 and 53 cognitive networks, respectively (*P*_*Moran&FDR*_ < 0.05, Fig. 4D, Additional file 1: Table S11). The functions of the 31 positive networks were related to sensation and movement (such as “perception,” “pain,” and “multisensory”). The 53 anti-correlated networks were associated with cognitive functions (such as “intelligence,” “reasoning,” and “judgment”).

### Alignment with therapeutic targets

**Fig. 5:**
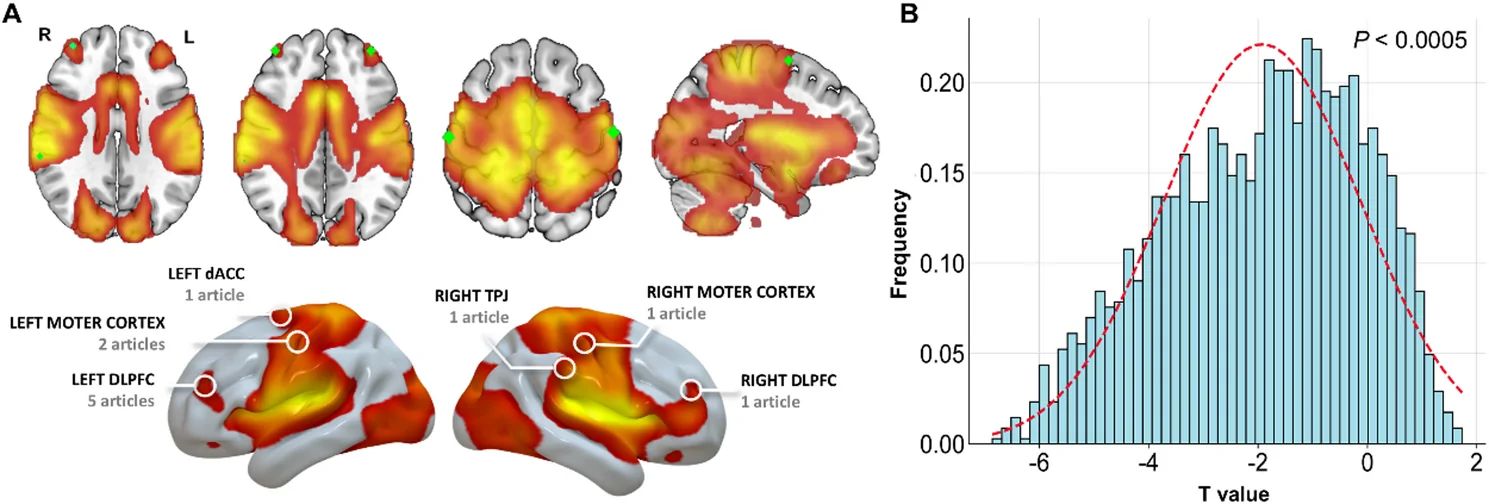
Alignment with therapeutic targets. **A** Spatial location of effective brain stimulation targets. **B** We randomly redistributed somatic hub regions 2,000 times to generate random networks. Based on the distribution of the targets in these random networks, we found that the targets were significantly co-localized with the somatic network (permutation test, *p* < 0.0005,). Abbreviations: DLPFC, dorsolateral prefrontal cortex; dACC, dorsal anterior cingulate gyrus; TPJ, temporal parietal junction

We identified 11 effective brain stimulation targets from 8 clinical studies. (Fig. 5A, Additional file 1: Table S12). The target ROIs revealed significant spatial convergence within the somatic network (one-sample t-test: *t* = 3.145, *p* = 0.014). To further validate this finding, we randomly redistributed somatic hub regions 2,000 times to generate random networks. The targets and network connectivity values in the somatic network were significantly higher than in random networks (*p* < 0.0005) (Fig. 5B).

#### Personalized symptom-specific atrophy identification

**Fig. 6:**
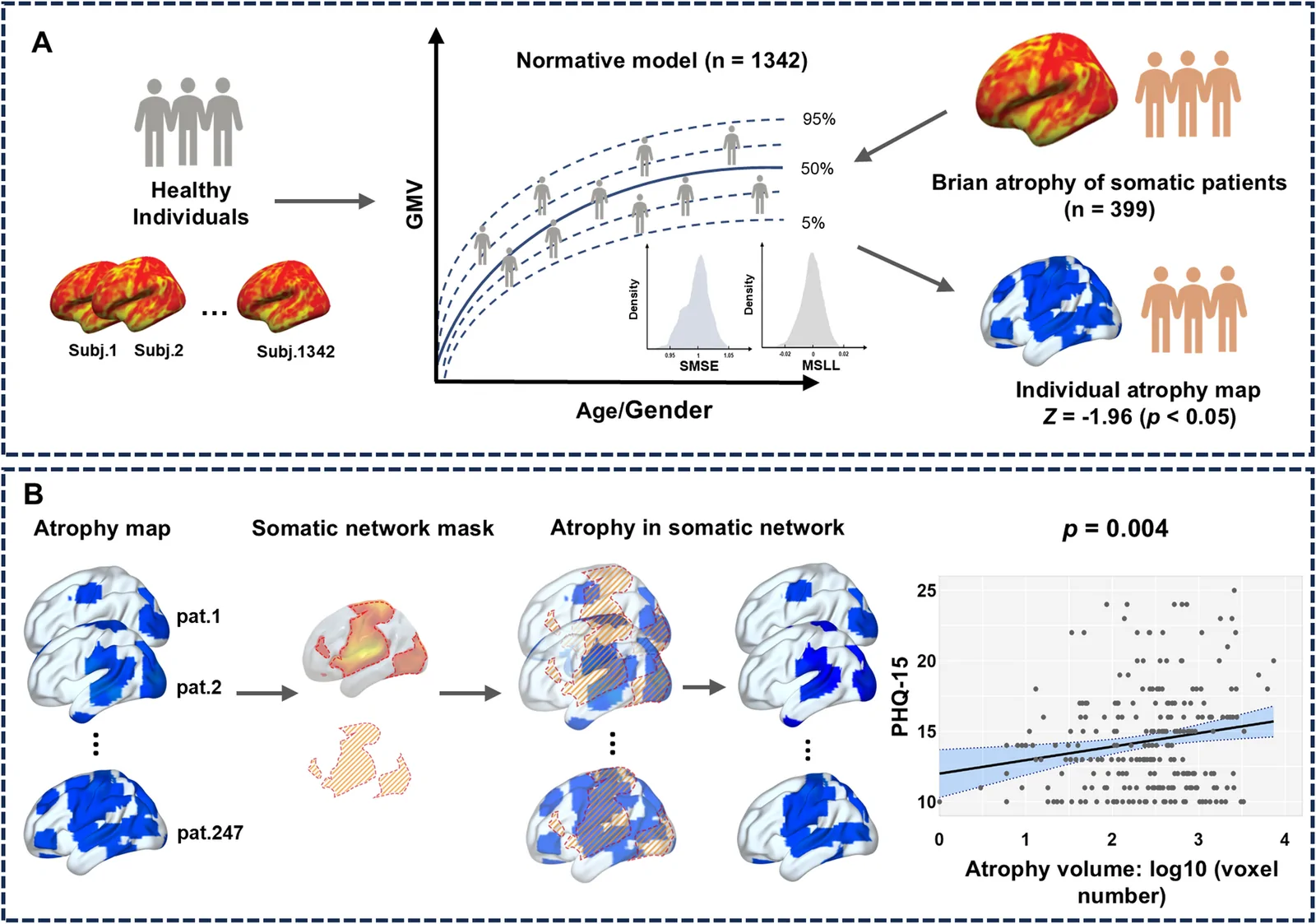
Individual application of the somatic network.** A** We established the normative model of gray matter volume by gaussian process regression on the healthy control dataset and estimated its performance using independent data. Through this model, we identified the atrophy map for each somatic patient (threshold *z* = -1.96). **B** The personalized atrophy volume (i.e., total voxel number) within the somatic network showing significant correlation with the PHQ-15 scores

We estimated a normative model of GMV as a function of age and sex using GPR in a structural MRI dataset of 1342 HCs. (Fig. 6A) The normative model’s performance demonstrated high generalizability when evaluated by explained variance, overall standardized mean squared error, and mean squared log-loss metrics (Additional file 1: Figure S10). When applied it to 399 somatic patients, the highest overlapping of personalized atrophy loci was 7% (Additional file 1: Figure S11), suggesting great heterogeneity. The atrophy volume within the somatic network was significantly positively correlated with somatic symptom severity, as assessed by the PHQ-15 scores (*r* = 0.182, *p* = 0.004) (Fig. 6B). This correlation remained significant after controlling for age, sex and total atrophy volume (Additional file 1: Figure S12). Specifically, the atrophic volume within the somatic network revealed no significant correlation with other symptoms, including anxiety assessed by the Hamilton Anxiety Rating Scale (*r* = 0.071, *p* = 0.263) or depression assessed by the Hamilton Depression Rating Scale (*r* = 0.053, *p* = 0.404) (Additional file 1: Figure S13).

## Discussion

There are four important findings in the present study. First, brain lesions causing somatic symptoms were anatomically distant but functionally converged on a common brain network. Second, the lesion-derived somatic network was validated by neuroimaging signatures of somatic symptoms and interoceptive circuits. Third, the somatic network aligned with effective brain stimulation targets, suggesting its potential therapeutic relevance. Fourth, the somatic network may help identify somatic symptom-specific atrophy areas, which could inform future research on personalized and symptom-specific stimulation. Collectively, these findings provide preliminary insights into the network-level neural mechanisms of somatic symptoms and may inform future development of network-guided therapeutic strategies.

The insula and putamen, as hub regions of somatic symptoms, have been implicated in cognitive functions relevant to somatic symptoms [75, 76]. Since the formulation of the “somatic marker hypothesis” [77], the insula has attracted increasing attention for its central role in integrating multimodal sensory, motor, and interoceptive signals to support emotional, cognitive, and motivational processes [78–80]. The putamen also acts as a key node in both cognitive and sensorimotor circuits, suggesting its involvement in both the etiology and clinical manifestations of somatic symptoms [81, 82]. Additionally, the somatic network predominantly overlapped with the SMN, SCN and VAN. The SMN plays a primary role in integrating and regulating sensory-motor information, which is significantly associated with somatic symptoms [83, 84]. The SCN includes thalamic, basal ganglia, and limbic structures that mediate sensory-affective integration [85, 86]. The VAN not only facilitates stimulus processing and transmission but also plays a crucial role in attention, emotion, and cognitive regulation [87]. Dysfunction in both SCN and VAN has been observed in various somatic-related conditions [88, 89]. Our findings suggest that the heterogeneity in prior neuroimaging findings could be unified in a common somatic network. It is worth noting that we primarily focused on the positive network, which is more interpretable [24, 90]. While emerging evidence suggests that negative functional connectivity may be also meaningful. For example, anticorrelation between DMN and task-positive networks supports the balance between internally directed cognition and externally oriented attention, and abnormalities in this balance are linked to psychiatric disorders [91, 92]. However, our exploratory study observed that the abnormal somatic brain regions, interoceptive circuits, and effective therapeutic targets were all predominantly located within the positive network. We therefore consider the positive values of the somatic network to be more meaningful, and this finding was further validated using normative models.

Interoception—the perception of internal bodily states—regulates homeostasis, motivation, and emotional processing [93]. When dysregulated, it leads to complex central–peripheral symptom interactions commonly seen in irritable bowel syndrome, chronic pain syndromes, fibromyalgia, and functional neurological disorders [94, 95]. Specifically in somatic symptoms, patients exhibit both impaired interoceptive processing and related cognitive deficits [96, 97]. Interoceptive dysfunction is generally believed to significantly contribute to the psychological mechanisms of somatic symptoms [93, 98]. Consistently, the insula—the primary cortical site for interoceptive representation [99]—serves as a hub region of the somatic network. Additionally, the spatial consistency with the interoceptive circuit validates our somatic network from the perspective of psychological mechanism.

As a newly identified brain circuit, the somatic network underwent functional annotation integrating genomic, neurochemical, and cognitive data to bridge the gap between brain network organization and underlying pathophysiological mechanisms. Gene enrichment analysis revealed significant involvement in ontology terms such as “detection of stimulus involved in sensory perception of pain,” indicating sensory processing abnormalities in somatic symptoms. Furthermore, the somatic network exhibited FC with inflammatory pathways, supporting a well-established link between neuroimmune interactions and emotion regulation [100, 101]. These findings suggest that somatic symptoms may essentially involve immune-inflammatory responses driven by emotional dysregulation, positioning the network as a potential interface between emotion and peripheral physiological processes. Abnormal neurotransmitter release has long been recognized as a key mechanism underlying psychiatric disorders [102]. Our findings specifically implicated dopaminergic dysfunction in somatic symptoms, demonstrating significant associations with four dopamine transporters and receptors. The dopamine system features discrete projections to specific brain regions involved in motor behavior, cognition, and emotion [102–104]—functions that are consistently disrupted in somatic symptoms. Additionally, we revealed the involvement of serotonergic, GABAergic, noradrenergic, and cholinergic systems, all of which play crucial roles in regulating emotion and cognition [105–107], highlighting the multi-transmitter pathophysiology underlying somatic symptoms. These neurotransmitter systems represent key targets of first-line pharmacotherapies for somatic symptoms, including selective serotonin reuptake inhibitors (SSRIs), serotonin-norepinephrine reuptake inhibitors (SNRIs), and GABAergic modulators [108]. Somatic network may serve as a convergence hub mediating the therapeutic effects of these drugs, thereby providing a mechanistic foundation for optimizing pharmacological interventions. Cognitive processing analysis revealed not only a significant positive correlation between somatic symptoms and sensorimotor-related processes but also a negative correlation with higher-order cognitive functions, such as executive function. These findings suggest that perceptual hyper-processing, resulting from cognitive dysfunction, may contribute to the severity of somatic symptoms [109]. Somatic network may provide a direction for investigating the neurocircuitry underlying cognitive and behavioral characteristics in somatic patients.

Neuromodulation approaches have demonstrated therapeutic efficacy for somatic symptoms including chronic pain, fibromyalgia, and functional neurological disorders [110–114]. The modulated brain regions mainly include the dorsolateral prefrontal cortex, primary motor cortex and temporoparietal junction [111, 115, 116]. However, a consistent principle explaining why these brain areas are efficient is lacking. In this study, we found a high alignment of these targets with the somatic network, supporting a network theory to optimize the neuromodulation protocol. A specific direction would be choosing the damaged area within the somatic network of each patient as a personalized modulation target. Normative modeling is a promising approach to identify the individual brain atrophy maps of patients. However, the atrophy maps are highly heterogeneous, with a diffuse distribution that lacked clear clinical interpretability. In contrast, the somatic network derived from LNM is considered as a symptom-specific brain network. We combined atrophy maps with the somatic network and successfully extract symptom- specific atrophy components. This finding may suggest an explorable scope for individualized diagnosis, treatment, and outcome monitoring. Given that atrophy is more closely linked to disease pathology and lesion-based networks, we focused only on regions with extreme atrophy rather than those with increased volume. This approach combines individual-level statistical inference with network analysis, which aligns with the framework proposed in previous studies [13, 22]. 

Despite the strengths of this study, including causal network mapping with multiple validations and potential clinical application, several limitations should also be considered. First, we focused on the heterogeneous somatic group, hypothesizing that cross-disease somatic symptoms may localize in a common brain network. However, we did not deny the diversity of somatic symptoms, the heterogeneity of included cases may affect the specificity of the obtained somatic network and cannot reflect the unique network of a particular type of symptom. Further classification analysis should help clarify the specific mechanisms associated with different subtypes of somatic symptoms. Second, the number of brain lesions associated with somatic symptoms in our study was small, primarily because of strict inclusion criteria and the limited availability of relevant studies. The literature search was limited to three databases and two languages. Heterogeneous imaging parameters across original studies may also compromise result consistency. Nevertheless, previous studies have suggested that this sample size is methodologically adequate for network mapping [90, 117]. The robustness of the network was checked across various multi-dimensional validation, and its translational value is supported by alignment with neuromodulation targets and ability to delineate somatic symptom-related brain atrophy. These evidences helped mitigate the impact of the small sample size. Third, a causal inference of lesion network mapping cannot be established with absolute certainty, because of absence of prospective or interventional validation. Future longitudinal interventional studies will further explore the therapeutic utility of the somatic network and strengthen the causal link between the network and symptom. Fourth, we found that effective stimulation targets significantly colocalized with the somatic network. However, this analysis only confirms spatial overlap and does not directly demonstrate that the therapeutic effects are mediated by this network. Future prospective studies examining the somatic network’s ability to guide clinical treatment and predict outcomes will be essential for further validation and clinical translation. In addition, the respective roles of gray matter and white matter in the network were not differentiated. As the fMRI signal in white matter has been recognized as an important measure in understanding brain function [34, 118, 119], future studies may integrate white matter function to construct a more comprehensive conclusion [120].

## Conclusions

By integrating multimodal data from brain lesions, neuroimaging, and neuromodulation, this study derived a brain network that may help explain why somatic symptoms persist across a wide spectrum of diseases with heterogeneous abnormal brain regions. The combined framework of the somatic network and normative model offers a strategy for future research to define symptom-specific neural targets, potentially advancing the therapeutic efficacy of neuromodulation.

## Supplementary Information

Below is the link to the electronic supplementary material.

Supplementary Material 1: Additional file 1: Method S1-S8, Table S1-S13, Figure S1-S13

Supplementary Material 2: Additional file 2: PRISMA checklist

Supplementary Material 3: Additional file 3: COBIDAS checklist

## Data Availability

The raw neuroimaging data are protected and are not available due to data privacy laws. The processed neuroimaging data is available upon reasonable request to the corresponding author. The NIfTI files of brain lesions causing somatic symptoms and the somatic network image, text files containing all coordinates of validation analysis, and Excel file of correlation results from the normative model are available at (https://github.com/Yuyue06116/somatic-network ). The code for the preprocessing and connectivity analyses is available as part of the open-access WhiteMatterSF software (https://github.com/jigongjun/Neuroimaging-and-Neuromodulation; https://doi.org/10.5281/zenodo.14252802).

## Abbreviations

5-HT1AR: 5-hydroxytryptamine 1 A receptor
5-HT1BR: 5-hydroxytryptamine 1B receptor
5-HT2AR: 5-hydroxytryptamine 2 A receptor
5-HT4R: 5-hydroxytryptamine 4 receptor
5-HTT: 5-hydroxytryptamine transporter
AHBA: Allen Human Brain Atlas
AHMU: Anhui Medical University
ALFF: amplitudes of low-frequency fluctuations
AMHC: Anhui Mental Health Center
D1R: Dopamine D1 receptor
D2R: Dopamine D2 receptor
DAT: Dopamine transporter
FC: functional connectivity
F-DOPA: Fluorodopa
FDR: False Discovery Rate
FWE: family-wise error
GABAAR: γ-aminobutyric acid type A receptor
GMV: gray matter volume
GPR: gaussian process regression
HCP-MMH: Human Connectome Project-Multi-Modal Parcellation
HCs: healthy controls
LNM: lesion network mapping
LSD: Least Significant Difference
mGluR: metabotropic glutamate receptor
MNI: Montreal Neurological Institute
NAT: norepinephrine transporter
PHQ-15: Patient Health Questionnaire-15
ReHo: regional homogeneity
ROIs: regions of interest
SCN: subcortical network
SMN: sensorimotor network
SNRIs: serotonin-norepinephrine reuptake inhibitors
SSRIs: selective serotonin reuptake inhibitors
tACS: transcranial alternating current stimulation
task-fMRI: task-based functional MRI
tDCS: transcranial direct current stimulation
TMS: transcranial magnetic stimulation
USTC: University of Science and Technology of China
VAChT: vesicular acetylcholine transporter
VAN: ventral attention network
