# Supplementary material for: A lesion-derived brain network of somatic symptoms for transdiagnostic individual application
Source: BMC Med. 2026 Jul 2;24:510. doi: 10.1186/s12916-026-05044-y (PMC13599187; doi:10.1186/s12916-026-05044-y)
Supplement: Supplementary file 1 [file 12916_2026_5044_MOESM1_ESM.docx]

**Additional file 1**

**A Lesion-derived Brain Network of Somatic Symptoms for Transdiagnostic Individual Application**

**Yu *et al.***

**Contents**

[**Additional file 1**	1](#_Toc233124437)

[**Supplementary Methods**	4](#_Toc233124438)

[Method S1: Literature search and selection	4](#_Toc233124439)

[Method S2: Details of the discovery and validation databases	4](#_Toc233124440)

[Method S3: MRI preprocessing	5](#_Toc233124441)

[Method S4: Specificity analysis	6](#_Toc233124442)

[Method S5: Validation of multimodal imaging findings in somatic symptoms	6](#_Toc233124443)

[Method S6: Network annotation of gene	7](#_Toc233124444)

[Method S7: Alignment with therapeutic targets	8](#_Toc233124445)

[Method S8: Normative model analysis	8](#_Toc233124446)

[Imaging dataset acquisition	8](#_Toc233124447)

[Estimation of gray matter volume (GMV)	9](#_Toc233124448)

[Normative modeling for GMV	9](#_Toc233124449)

[**Supplementary Tables**	11](#_Toc233124450)

[Table S1. Demographic information of discovery and validation databases.	11](#_Toc233124451)

[Table S2. Characteristics of brain lesions causing somatic symptoms.	12](#_Toc233124452)

[Table S3. Characteristics of brain lesions causing control symptoms	14](#_Toc233124453)

[Table S4. Sample and imaging characteristics of studies included in the resting-state activation analysis of somatic symptoms	20](#_Toc233124454)

[Table S5. Sample and imaging characteristics of the studies included in the gray matter volume analysis of somatic symptoms	21](#_Toc233124455)

[Table S6. Sample and imaging characteristics of the studies included in the task-induced activation analysis of somatic symptoms	22](#_Toc233124456)

[Table S7. Sample and imaging characteristics of the studies included in structure, resting-state activation or task-induced activation analysis of control symptoms	23](#_Toc233124457)

[Table S8. Sample and imaging characteristics of the studies included in the task-induced activation analysis of interoceptive processing	26](#_Toc233124458)

[Table S9. Sample and imaging characteristics of the studies included in the task-induced activation analysis of control behavioral processing	29](#_Toc233124459)

[Table S10. Gene networks showing similar spatial similarity with the somatic network	31](#_Toc233124460)

[Table S11. Cognitive networks showing similar spatial correlation with the somatic network	33](#_Toc233124461)

[Table S12. Sample and imaging characteristics of brain stimulation improving somatic symptoms	35](#_Toc233124462)

[Table S13. Demographic and clinical characteristics of the participants in normative model analysis	36](#_Toc233124463)

[**Supplementary Figures**	37](#_Toc233124464)

[Fig. S1. Flow diagram of the literature search for identifying lesions causing somatic symptoms.	37](#_Toc233124465)

[Fig S2. Brain lesions causing somatic symptoms.	38](#_Toc233124466)

[Fig. S3. Flow diagram of the literature search for identifying brain structural and functional abnormalities of somatic symptoms.	39](#_Toc233124467)

[Fig. S4. Lesion network mapping in disease connectome (n = 190).	40](#_Toc233124468)

[Fig. S5. Lesion network mapping using a 4mm lesion extension.	41](#_Toc233124469)

[Fig. S6. Lesion network mapping using 85% overlapping regions of the sensitivity map and both specificity maps.	42](#_Toc233124470)

[Fig. S7. Comparison of 4 somatic lesion networks.	43](#_Toc233124471)

[Fig S8 Validation of multimodal imaging findings in somatic symptoms by symptom.	44](#_Toc233124472)

[Fig S9 Validation of multimodal imaging findings in somatic symptoms by indicator.	45](#_Toc233124473)

[Fig. S10. Model fit evaluation.	46](#_Toc233124474)

[Fig. S11. Individual atrophy loci.	47](#_Toc233124475)

[Fig. S12. Partial correlation analysis between atrophy volume in somatic network and PHQ-15 scores (controlling for age, sex and total atrophy volume as covariates).	48](#_Toc233124476)

[Fig. S13. Correlation analysis between atrophy volume in somatic network and HAMA, HAMD scores.	49](#_Toc233124477)

# Supplementary Methods

## Method S1: Literature search and selection

A comprehensive and systematic literature search in the PubMed, Web of Science and Embase databases was performed to identify brain lesions causing somatic symptoms before December 1, 2024 following PRISMA guidelines. We used a combination of the following search terms: “somatic symptom*”, “physical symptom*”, “somatic disorder”, “somatoform symptom*”, “somatoform disorder”, “somatization symptom*”, “somatization disorder”, “somatisation symptom*”, “somatisation disorder”, “lesion”, “stroke”, “infarct”, “ischem*”, “ischaem*”, “hemorrhag*”, “tumor”, “tumour”, “MRI”, “magnetic resonance imaging”, “fMRI”, “functional magnetic resonance imaging”, “CT”, “computed tomography”. In addition, the reference sections of the retrieved studies and review articles were systematically reviewed to identify additional relevant sources. To be included, papers needed to meet pre-defined inclusion criteria: 1) English reports describing 2) patients (case reports, case series, letters, and observational studies), with 3) new-onset somatic symptoms or somatic symptoms attributed to 4) lesions of CNS, present on most days for at least several months. And 5) had structural images of sufficient quality for lesion tracing. The exclusion criteria included: 1) cases where lesions could not be localized to a clearly delineated intra-parenchymal lesion, such those cases where lesions were due to extrinsic compression injuries; 2) lesions caused by a surgical complication; 3) physical or somatic symptoms are obvious traditional focal signs, such as hemiplegia caused by basal ganglia lesion; 4) a pre-existing patient history of somatic symptoms. A flow diagram of the literature selection process is presented in Figure S1. The lesions were mapped onto a 3D template for subsequent functional connectivity calculations and overlap analysis.

We checked the reference lists and review articles to identify studies that may have been missed in the original search. Two researchers (Y.Y. and Q.L.) independently searched the articles. Our search was limited to English or Chinese and human articles. If there were some inconsistent opinions, the two researchers reached a consensus result through the discussion under the guidance of the corresponding author (T.Y. and J.G.). This protocol was registered (<https://www.crd.york.ac.uk/PROSPERO/>, registration number: CRD42024623688). The included literatures were presented in Table S2.

## Method S2: Details of the discovery and validation databases

We collected resting-state functional MRI (rs-fMRI) and structural MRI of 652 healthy individuals (mean age ± SD = 22.9 ± 5.53 years; 334 females) from the community, and 190 somatic patients (median age = 35 years; IQR: 26–48; 130 females) from Anhui Mental Health Center (AMHC) or The First Affiliated Hospital of Anhui Medical University (AHMU). The inclusion criteria of healthy controls (HCs) included: 1) no diagnosis of any neuropsychiatric disorder; 2) no history of alcohol or substance dependence; 3) normal intelligence, with the ability to complete basic neuropsychological assessments. 4) right-handed. Exclusion criteria included: 1) presence of metal implants that do not meet the safety requirements for MRI screening, including but not limited to pacemakers, cardiac stents, cochlear implants, metal dentures, deep brain stimulators, and other surgically implanted metallic devices; 2) history of major brain injury or significant surgical procedures, including but not limited to internal fixation with steel plates after fractures, and post-shunt procedures requiring stents in the liver, heart, or kidneys; 3) presence of severe systemic diseases such as cardiovascular disease, liver disease, or kidney disease.

The inclusion criteria for somatic patients included: 1) Patient Health Questionnaire-15 (PHQ-15) score > 5, which represents symptom burden existence; 2) There is at least one specific somatic symptom, such as gastrointestinal symptoms, chronic pain; 3) somatic symptoms present on most days for at least several months; 4) right-handed. The exclusion criteria were as follows: 1) individuals aged < 18 or > 60 years; 2) contraindications for MRI; 3) severe somatic disorders were excluded to avoid interference on PHQ-15 scores. we mainly recruited patients with anxiety and depression accompanied by somatic symptoms. The demographic information is presented in Table S1.

MRI data were obtained at the University of Science and Technology of China (USTC) with a 3-T scanner (Discovery GE750w; GE Healthcare, Milwaukee, WI, USA). Rs-MRI images were acquired using a single shot gradient‒recalled echo planar imaging sequence (repetition/echo time, 2400/30 ms; flip angle, 90; field of view, 192 × 192 mm^2^; 64 × 64 in‒plane matrix; section thickness, 3 mm; voxel size, 3 × 3 × 3 mm^3^; 46 transverse sections). A total of 217 volumes were acquired (~ 8.7 min). During scanning, participants were instructed to rest with their eyes closed without falling asleep. High-resolution T1-weighted images were acquired in the sagittal orientation using a magnetization prepared rapid gradient-echo sequence (repetition/echo time, 8.16/3.18 ms; flip angle, 12; field of view, 256 × 256 mm^2^; 256 × 256 matrices; section thickness, 1 mm; voxel size, 1 × 1 × 1 mm^3^). All resting-state fMRI and T1-weighted sequences achieved whole-brain coverage. The cerebellum and brainstem were fully included in the scanning range. Axial slices were acquired coplanar with the AC-PC (anterior commissure-posterior commissure) line.

All acquired brain MRI scans were reviewed by professional neuroradiologists for incidental findings. Participants with clinically significant intracranial abnormalities were excluded from the dataset and referred for clinical follow-up. Data from participants with benign, non-clinical incidental findings were kept for analysis.

## Method S3: MRI preprocessing

Rs‒fMRI data were preprocessed using SPM12 software (https://www.fil.ion.ucl.ac.uk/spm/) and ANFI (https://afni.nimh.nih.gov/afni/). The processing steps were as follows: 1) data conversion: the images were converted from DICOM to NIfTI; 2) delete time points: we deleted the first 5 time points to ensure signal equilibrium and participant adaptation to scanner noise; 3) dispiking: remove temporal spikes more than 2.5 SDs from the mean (AFNI’s 3dDespike); 4) slice timing: adjusted temporal offsets between slices to synchronize acquisition times across all image layers; 5) head motion correction: head motion was quantified by calculating translational displacements (x/y/z axes) and rotational changes (pitch/roll/yaw) for each volume. All included participants exhibited head motion below predefined thresholds (< 3 mm translation or < 3° rotation); 6) co‒registration to the structural image; 7) regress out nuisance regressors: 24 head motion parameters, and average signals in the cerebrospinal fluid, white matter, and whole brain; 8) spatial normalization: normalize to the MNI space using the matrix produced by structural image segmentation using DARTEL algorithm in SPM12; 9) spatial smoothing: smooth with a 4 mm full width at half maximum Gaussian kernel.

For each subject, Pearson correlation coefficients were calculated between the mean time series of each brain lesion ROI and the time series of every brain voxel. Correlation values were converted via Fisher’s r-to-z transformation for normality, generating individual RSFC maps for LNM analysis. All analyses ran on a local Windows 11 workstation with Intel Core i9 CPU

## Method S4: Specificity analysis

To identify somatic‒specific regions in the sensitivity map, we conducted a specificity analysis between the networks derived from 21 somatic cases and those from two distinct control groups:1) networks generated from 97 non‒somatic related brain lesions by local‒to‒network mapping; 2) random‒control networks. The 97 brain lesions causing control symptoms include amnesia, insomnia and aphasia. We selected cases of brain lesions causing amnesia from a recently published study. We finally included 53 cases of brain lesion resulting in aphasia. We selected cases of brain lesions causing insomnia by performing a systematic literature search on PubMed, Web of Science and Embase databases. The search string combined terms: “insomnia*”, “lesion”, “stroke”, “infarct”, “ischem*”, “ischaem*”, “hemorrhag*”, “tumor”, “tumour”, “MRI”, “magnetic resonance imaging”, “fMRI”, “functional magnetic resonance imaging”, “CT” and “computed tomography”. To be included, papers needed to meet pre-defined inclusion criteria: 1) English reports describing 2) patients (case reports, case series, letters, and observational studies), with 3) new-onset insomnia symptoms attributed to 4) lesions of CNS. And 5) had structural images of sufficient quality for lesion tracing.

We finally included 24 cases of brain lesion resulting in insomnia (Table S3). The 20 aphasia cases were part of the post-stroke aphasia patients we collected in AHMU (detailed information can be found in our previous study). The random‒control networks were calculated by local‒to‒network mapping using the randomly generated seed points, which with the same voxel count as the brain lesions causing somatic symptoms.

## Method S5: Validation of multimodal imaging findings in somatic symptoms

To validate the somatic network, we also conducted a systematic literature search in the PubMed, Web of Science and Embase databases, identifying somatic-related imaging abnormalities in brain structure (gray matter volume) or function (task-induced activation or resting-state activity). We used a combination of the following search terms: “somatic symptom*”, “physical symptom*”, “somatic disorder”, “somatoform symptom*”, “somatoform disorder”, “somatization symptom*”, “somatization disorder”, “somatisation symptom*”, “somatisation disorder”, “MRI”, “magnetic resonance imaging”, “neuroimaging”, “ReHo”, “regional homogeneity”, “ALFF”, “fALFF”, “amplitude of low frequency fluctuation”, “VBM”, “voxel-based morphometry”, “GMV”, “gray matter”, “grey matter”. The literature search was not limited to a specific disease. Eligible studies fulfilled the following inclusion criteria: 1) studies compared gray matter volume, resting-state activity and task-induced activation between somatic and non-somatic individuals; 2) comparisons were conducted in a voxel-wise manner and at a whole-brain level; 3) significant results were corrected for multiple comparisons; 4) coordinates of significant brain clusters were reported in standard reference space (Montreal Neurological Institute [MNI] space or Talairach). The exclusion criteria included: 1) studies did not report the coordinate system; 2) studies used region of interest analyses; 3) all reported coordinates were outside the gray matter; 4) studies were reviews or meta-analyses. A flow diagram of the literature selection process is presented in Figure S3. We focused our analysis on contrasts rather than studies. The coordinates of peak voxels from significant clusters identified in each contrast were extracted. The included symptoms and studies were presented in (Table S4-S6).

We hypothesized that somatic-related region of interests (ROIs) would exhibit stronger resting-state functional connectivity (RSFC) to the somatic network than non-somatic and random ROIs. To identify non-somatic control ROIs, we included aphasia, pathological gambling, insomnia and suicide as control symptoms. The neuroimaging abnormalities of aphasia, pathological gambling and suicide were included from recently published studies. The neuroimaging abnormalities of insomnia were searched by a systematic literature search on PubMed, Web of Science and Embase databases. The search string combined terms: “insomnia*”, “MRI”, “magnetic resonance imaging”, “neuroimaging”, “GMV”, “gray matter”, and “grey matter”. (Table S7) The average t-value within each ROI from the somatic network (t map) were extracted and performed Mann-Whitney tests to compare the somatic-related ROIs with both the non-somatic and random control ROIs.

## Method S6: Network annotation of gene

We adopted risk genes of somatic symptoms from the largest genome-wide association study (GWAS). We selected the four representative disease risk genes of somatic symptoms, which include Fibromyalgia, irritable bowel syndrome, chronic pain and somatoform symptoms. The risk genes were derived from recently published reviews. In line with our previous studies, gene expression maps were constructed from the left brain of Allen Human Brain Atlas (AHBA) (http://human.brain-map.org) using the abagen toolbox (v.0.1.3; https://github.com/rmarkello/abagen). The AHBA was based on 3,702 spatially distinct samples from six post-mortem brains (age = 42.50 ± 13.38 years; male/female = 5/1) and included expression maps of 214 somatic risk genes. The microarray probes were reannotated, and probes not matched to a valid Entrez ID were discarded. Probes with intensity less than a background in ≥ 50.00% of samples across donors were discarded, yielding 31,569 probes. When multiple probes indexed the expression of the same gene, we selected the probe with the most consistent pattern of regional variation across donors (that is, with the highest differential stability). Gene expression values were then normalized across tissue samples using an identical procedure, and computed in left brain ROIs. These ROIs included 160 regions from HCP-MMH atlas, 9 cerebellum, and 4 subcortical regions from the automatic anatomical labeling template. We only chose ROIs in the left hemisphere because the microarray probes in the right hemisphere are not available in two of the AHBA brains. Samples assigned to the same ROI were averaged separately for each donor and then across donors, yielding expression maps of 214 risk genes. These expression maps were transformed into brain networks through local-to-network mapping. The gene enrichment analysis was conducted by Metascape (https://metascape.org/), to perform Kyoto Encyclopedia of Genes and Genomes (KEGG) and Gene Ontology (GO) biological processes analyses. The background genes were obtained by excluding probes if they did not exceed the background in at least 20% of samples after probe to gene re-annotation. The obtained biological processes were corrected by FDR correction (*p* < 0.05).

## Method S7: Alignment with therapeutic targets

To evaluate whether the identified somatic network serves as a potential target network for clinical treatment, we conducted a literature search to identify brain stimulation which improve somatic symptoms, including transcranial magnetic stimulation (TMS), transcranial direct current stimulation (tDCS), and transcranial alternating current stimulation (tACS). The search string combined terms: “somatic symptom*”, “physical symptom*”, “somatic disorder”, “somatoform symptom*”, “somatoform disorder”, “somatization symptom*”, “somatization disorder”, “somatisation symptom*”, “somatisation disorder”, “transcranial magnetic stimulation”, “TMS”, “transcranial direct current stimulation”, “tDCS”, “transcranial alternating current stimulation”, “tACS”, “deep brain stimulation” and “DBS”. Studies demonstrated effectiveness in treating somatic symptoms and provided MNI coordinates of stimulation sites or a clear anatomical depiction of stimulation sites were selected. The included studies were presented in Table S12.

## Method S8: Normative model analysis

### *Imaging dataset acquisition*

Structural MRI databases of 1342 HCs and 399 somatic patients were collected from AMHC or AHMU. The inclusion criteria of HCs included: 1) no diagnosis of any neuropsychiatric disorder; 2) no history of alcohol or substance dependence; 3) Normal intelligence, with the ability to complete basic neuropsychological assessments; 4) right-handed. Exclusion criteria included: 1) presence of metal implants that do not meet the safety requirements for MRI screening, including but not limited to pacemakers, cardiac stents, cochlear implants, metal dentures, deep brain stimulators, and other surgically implanted metallic devices; 2) history of major brain injury or significant surgical procedures, including but not limited to internal fixation with steel plates after fractures, and post-shunt procedures requiring stents in the liver, heart, or kidneys; 3) presence of severe systemic diseases such as cardiovascular disease, liver disease, or kidney disease.

The inclusion criteria for somatic patients included: 1) PHQ-15 score > 5, which represents symptom burden existence; 2) there is at least one specific somatic symptom, such as gastrointestinal symptoms, chronic pain. 3) somatic symptoms present on most days for at least several months; 4) right-handed. The exclusion criteria were as follows: 1) individuals aged < 18 or > 60 years; 2) contraindications for MRI; 3) severe somatic disorders were excluded to avoid interference on PHQ-15 scores. We recruited primarily patients with psychiatric disorders accompanied by somatic symptoms, including anxiety, depression, bipolar disorder and schizophrenia. The demographic information is presented in Table S13. Partial data duplication in structural and functional databases.

MRI data were obtained at USTC. High-resolution T1-weighted images were acquired in the sagittal orientation using a magnetization prepared rapid gradient-echo sequence (repetition/echo time, 8.16/3.18 ms; flip angle, 12; field of view, 256 × 256 mm^2^; 256 × 256 matrices; section thickness, 1 mm; voxel size, 1 × 1 × 1 mm^3^). Axial slices were acquired coplanar with the AC-PC (anterior commissure-posterior commissure) line.

### *Estimation of gray matter volume (GMV)*

We conducted GMV analysis using Computational Anatomy Toolbox 12 (CAT12, https://neuro-jena.github.io/cat//) within the SPM12 framework. The preprocessing workflow was as follows: 1) Data conversion and quality check: T1-weighted structural images were converted from DICOM to NIfTI format using MRIcron, selecting reoriented images for preprocessing. A quality check was performed to exclude scans with incomplete acquisition or artifacts; 2) Tissue segmentation: T1-weighted images were segmented into gray matter (GM), white matter (WM), and cerebrospinal fluid (CSF) using tissue probability maps (TPM) to enhance classification accuracy; 3) Spatial normalization: segmented GM probability maps were registered to the MNI152 template via initial affine transformation, standardizing spatial coordinates and reducing inter-subject anatomical variability; 4) Nonlinear registration: optimized Shooting was used for nonlinear registration, refining spatial alignment for greater accuracy than traditional DARTEL. GM probability maps were resampled to 1.5 × 1.5 × 1.5 mm³; 5) Modulation and volume calculation: Jacobian determinant modulation was applied to GM probability maps, generating reliable GMV measures; 6) Data Quality Assessment: the "Get weighted overall image quality" function in CAT12 evaluated image clarity, noise, and artifacts, generating an interquartile range (IQR) quality score for exclusion of substandard scans; 7) Smoothing: A Gaussian kernel (8 × 8 × 8 mm) was applied to enhance the signal-to-noise ratio (SNR), mitigate misalignment effects, and ensure statistical normality. The smoothed GMV maps were then used for statistical analysis.

### *Normative modeling for GMV*

We first utilized a predefined functional parcellation template to divide the whole brain into 1,032 distinct regions, comprising 1,000 cortical and 32 subcortical regions. We computed the GMV of each region for subjects in 1342 HCs. We then estimated a normative model of GMV as a function of age and sex by using Gaussian process regression (GPR). GPR is a Bayesian nonparametric interpolation method that yields coherent measures of predictive confidence alongside point estimates. Briefly, a Gaussian process specifies a distribution over functions, such that any finite number of elements has a joint Gaussian distribution. A GP can be denoted by $N\left( m\left( x \right), k\left( x, x^{'} \right) \right)$, and is uniquely specified by a mean $\left( m\left( x \right) \right)$ and covariance $(k (x, x'))$ function. Gaussian process models can be used for Bayesian nonlinear regression based on a set of training data $D=\left\{ x_{i}{,y}_{i} \epsilon\right\}_{ⅈ=1}^{N}$, where $x_{i}$ are the covariates (i.e., age, sex), $y_{i}$ are the response variables (i.e., GMV of each participates). We use an unconstrained latent function $(f = [f_{i}, \ldots, f_{N}]^{T})$ to model the relationships between data points, which is assumed to differ from the true response variables by additive Gaussian noise, i.e., $y_{i}=fi+\epsilon_{i}$, where $\epsilon_{i}\sim N\left( 0,\sigma_{n}^{2} \right)$. The goal is to estimate this function from the training data in such a way that it allows us to accurately predict a new target $y_{*}$ from the test data $x_{*}$. To reach this end, a GP prior distribution is placed over the latent function and its posterior distribution is computed using the Bayes rule. A detailed description of this method can be found in Sun et al. and Marquand et al. The estimation of the normative models was performed using the PCNtoolkit package (<https://github.com/amarquand/PCNtoolkit>). To assess the generalizability of the model, we use 80% of HCs as the training set and 20% as the test set for a simple cross-validation. The performance of the classifier on unseen data samples was assessed using the overall standardized mean squared error (SMSE) and mean squared log-loss (MSLL) (Figure S8).

# Supplementary Tables

## Table S1. Demographic information of discovery and validation databases.

| **Dataset** | **Sample size** | **Age (years)** | **Gender (M/F)** | **PHQ-15** |
| --- | --- | --- | --- | --- |
| Normative connectome | 652 | 22.9 (5.53) | 318/334 | - |
| Diseased connectome | 190 | 35 (26, 48) | 60/130 | 10 (8, 13) |

Age of normative connectome is expressed as mean (standard deviation). Age and PHQ-15 of disease connectome were expressed as median (interquartile range) because of non-normally distribution. Abbreviations: F, female; M, male; PHQ-15, health questionnaire-15.

## Table S2. Characteristics of brain lesions causing somatic symptoms.

| **Primary reference** | **DOI /** **PMID** | **# of**  **cases** | **Age** | **Gender** | **Location and etiology of lesion** |
| --- | --- | --- | --- | --- | --- |
| Mukherjee 2021 | 10.1016/j.eurpsy.2019.01.002 | 1 | 23 | F | Retro-cerebellar Arachnoid cyst in and around midline. |
| Pujol 2020 | 10.1016/S0925-4927(00)00061-5 | 1 | NA | NA | Demyelinating lesions in the left arcuate fasciculus region. |
| Kato 2006 | 10.1016/j.pscychresns.2005.10.003 | 1 | 64 | F | A small infarction in the right caudate nucleus. |
| Agrawal 2007 | 10.1016/j.clineuro.2007.07.007 | 1 | 16 | M | Chronic infarct in the territory of left middle cerebral artery. |
| Gandelman-Marton 2006 | 10.1097/01.nrl.0000215787.45207.1e | 1 | 24 | M | A low-grade astrocytoma in the right frontal lobe. |
| Nickell 1994 | 10.1002/anxi.3070010110 | 1 | 39 | F | Meningiomas in the right lateral ventricle impinging on hippocampus. |
| Serinken 2018 | 10.1017/s1049023x18000511 | 1 | 51 | F | Infarction on the precentral region of left frontal lobe. |
|  |  | 2 | 43 | F | Corticospinal tract hemorrhage extending from basal ganglia level to the mesencephalon |
| Haas 2022 | 10.1007/s10143-021-01660-9 | 1 | 22 | F | Infarction in right middle cerebral artery (MCA) territory. |
| Bowsher 2006 | 10.1159/000093575 | 1 | 39 | F | Small infarct in the upper lip of the left sylvian fissure. |
|  |  | 2 | 34 | F | A lesion in the depth of the left lateral fissure (arrows) also involving the posterior insula. |
|  |  | 3 | 30 | M | Lesion involving the upper bank of the sylvian fissure and posterior insula. |
| Huber 2008 | 10.1016/j.pnpbp.2008.09.014 | 1 | 77 | F | The scan showed multiple bilateral asymmetric lesions in the centrum semiovale and a small (lacunar) lesion in the posterior aspect of the left putamen. Two small lesions were found in the right mid portion of the external capsule on the border between caudate nucleus and claustrum. Signs of slight atrophy were evident. |
|  |  | 2 | 84 | F | The scan showed several small lesions in the bilateral centrum semiovale (deep white matter). A small lesion was present in the anterior portion of the left putamen and one in the left caudate nucleus. Two very small lesions were present on the right in the outer portion of the putamen as well as severe atrophy. |
|  |  | 3 | 82 | M | Some white matter lesions were found in the deep white matter and one in the mid posterior portion of the left putamen and in the caudate nucleus bilaterally. A small lesion was present in the left pons and the left thalamus as well as severe atrophy. |
|  |  | 4 | 73 | F | Previous surgery for meningioma (left temporal) with partial resection of the left temporal and frontal lobe was observed associated with marginal gliosis in the external capsule and outer margin of putamen and caudate nuclei. One small lesion was present in the mid portion of the putamen on the right. |
| Fontaine 1990 | 10.1016/0006-3223(90)90004-L | 1 | NA | NA | Three small foci of reduced signal in the medial portion of the right temporal lobe. |
|  |  | 2 | NA | NA | Asymmetric atrophy of themedial anterior horn of the temporal lobe. |
| Wessel 2009 | 10.1111/j.1600-0404.1994.tb02700.x | 1 | 68 | M | Paraniedian ischemic thalamic lesion in the territory of the interpeduncular profunda artery on the right side. |
|  |  | 2 | 60 | F | A posterolateral ischemic thalaniic lesion in the territory of the geniculothalaniic artery on the right side. |
|  |  | 3 | 85 | M | A anterolateral ischemic thalamic lesion in the territory of the tuberothalaniic artery on the right side. |

Patient identification, reference and characteristics of 21 cases of brain lesions causing somatic symptoms. Abbreviations: F, female; M, male; NA, not applicable.

## Table S3. Characteristics of brain lesions causing control symptoms

| **Primary reference** | **DOI /** **PMID** | **# of**  **cases** | **Age** | **Gender** | **Location and etiology of lesion** |
| --- | --- | --- | --- | --- | --- |
| *Insomnia* | | | | | |
| Aytaç 2019 | 10.18071/isz.72.0285 | 1 | 41 | F | Hyperintensities in both hippocampus and right occipital region. |
| Salih 2008 | 10.1212/01.wnl.0000312336.92028.9b | 1 | 64 | F | A singular, symmetric, non-enhancing T2-hyperintense pontomesencephalic lesion around the periaqueductal gray extending into the tegmentum. |
| Quigg 2007 | PMID: 17557455 | 1 | 66 | M | Bilateral ventralis intermedius nucleus |
| Priano 2008 | 10.1136/jnnp.2008.154815 | 1 | 53 | F | Cerebral MRI confirmed the presence of two ventricular shunts, one correctly placed in the left ventricle and the other ending in the right caudate nucleus |
| Papadopoulou 2005 | 10.1007/s10072-005-0457-y | 1 | 48 | M | An abnormal hyperintensive signal in the area of basal ganglia |
| Papantoniou 2024 | 10.22365/jpsych.2023.026 | 1 | 67 | F | Hemorrhage around the hypothalamus and the suprachiasmatic nucleus |
| Kulisevsky 1993 | 10.1212/wnl.43.7.1422 | 1 | 81 | F | A hypointense thalamic lesion at the level of the right ventral lateral and ventromedial nuclei |
| Szucs 2001 | 10.1159/000050760 | 1 | 53 | M | A space-occupying lesion in the posterior part of the left gyrus rectus extending to the subcallosal area and the septal region, displacing the anterior cerebral artery |
| Tamler 2006 | 10.1089/thy.2006.16.317 | 1 | 49 | M | A pituitary mass with heterogeneous enhancement, extending suprasellar into the prechiasmatic area and into the right cavernous sinus |
| Montiel 2008 | 10.1016/j.neurol.2007.08.007 | 1 | 63 | F | T1-weighted MRI showed right hippocampal atrophy. |
| Sagnier 2015 | 10.1016/j.neurol.2015.08.005 | 1 | 26 | M | A recent ischemic stroke located in the left anterior and mediodorsal thalamus, an area supplied by the pre-mamillary artery |
|  |  | 2 | 36 | M | An isolated left medio-dorsal thalamic stroke in the paramedian artery territory |
| Ayhan 2011 | 10.1016/s0028-3843(14)60129-8 | 1 | 28 | M | An ovoid-shaped cystic lesion in the pineal region without compression |
| Gruber 2007 | 10.1080/02699050701644111 | 1 | 55 | M | Selective damage of the left anterior temporal lobe including the temporal pole and underlying amygdala |
| Xu 2020 | 10.1080/00207454.2020.1860965 | 1 | 52 | M | Bilateral hyperintensity in the semioval centers on axial images and perivascular linear enhancement oriented radially to the ventricles on sagittal images |
| Mumoli 2024 | 10.1016/j.sleep.2024.02.047 | 1 | 55 | M | Brain-MRI showed hyperintensities on FLAIR sequences in the basal ganglia |
| Attarian 2009 | 10.1016/j.sleep.2009.02.004 | 1 | 67 | F | Sagittal and axial cuts of the MRI of the brain showing the original site of the bleed and the original AV malformation |
| Gai 2022 | 10.4103/0028-3886.338681 | 1 | 63 | F | a T1-weighted image of bilateral basal ganglia is normal, a T2-weighted image and fluid-attenated inversion recovery (FLAIR) image show hyperintensity of bilateral caudate nuclei and putamen. A diffusion-weighted image (DWI) shows restricted diffusion in bilateral putamen and caudate nuclei |
| Matsubara 2017 | 10.5692/clinicalneurol.cn-001048 | 1 | 70 | F | Diffusion-weighted imaging showed a high signal intensity in the right posterior limb of the internal capsule |
| Takado 2008 | 10.5692/clinicalneurol.48.338 | 1 | 65 | M | Axial and coronal FLAIR images showing right medial temporal high-intensity signal |
| Tirado-García 2023 | 10.7759/cureus.41595 | 1 | 18 | F | MRI fluid-attenuated inversion recovery (FLAIR) sequence. Bilateral hippocampal hyperintensities. Bilateral asymmetrical multifocal hyperintensities on cortical, subcortical, and periventricular zones. Diffusive bilateral lesions on frontal lobes white matter. |
| Shah 2024 | 10.7759/cureus.59387 | 1 | 48 | F | Paranasal sinuses demonstrated widening of the sella with an isodense lesion of approximately 3.3 × 1.8 cm^2^, involving the sella and suprasellar region |
| Truzzi 2021 | 10.5935/1984-0063.20200039 | 1 | 40 | F | MRI of the brain shows a residual lesion in the left nucleocapsular region |
| Zhang 2016 | 10.1186/s12883-016-0555-x | 1 | 40 | F | Diffusion-weighted magnetic resonance imaging (DWI) and the corresponding plane in fluid-attenuated inversion recovery (FLAIR) showed bilateral hyper-intensity of the medial temporal lobe, insular lobe and basal ganglia. |
| *Amnesia* | | | | | |
| Masuo 1999 | 10.1080/026990599121728 | 1 | 62 | M | A subcortical haematoma in the right occipital lobe with ventricular penetration. |
| Meissner 1987 | 10.1161/01.str.18.2.380 | 1 | 52 | M | Bilateral paramedian diencephalic infarctions with hyperemia |
| Mendizabal 1997 | 10.1046/j.1526-4610.1997.3709594.x | 1 | 47 | M | Bilateral infarctions of the posterior thalami,left occipital cortex, and left superior cerebellar hemisphere |
| Miranda 2015 | 10.1016/j.jns.2015.10.014 | 1 | 56 | F | Infarction with diffusion restriction and edema in the bilateral anterior columns of the fornix and caudate and lenticular nuclei bilaterally, with more involvement on the left |
| Moudgil 2000 | 10.1161/01.str.31.6.1418 | 1 | 71 | F | Acute ischemic lesions in anterior fornix and genu of corpus callosum |
| Moussouttas 2005 | 10.1159/000086104 | 1 | 61 | M | Acute ischemia of the bilateral anterior for nices, septum pellucidum and genu of the corpus callosum, and left frontal subcortical region |
| B R Ott 1993 | 10.1161/01.str.24.7.1033 | 1 | 67 | M | Middle and posteriorportions of left hippocampus and parahippocampal gyrus. |
|  |  | 2 | 65 | F | Left anterior hippocampus and amygdala. |
| Park SA 2000 | 10.1212/wnl.54.6.1379 | 1 | 60 | F | Discrete acute cerebral infarction in bilateral fornices and genu of right corpus callosum |
| Parkin 1994 | 10.1016/0028-3932(94)90067-1 | 1 | 48 | M | The hippoeampal pathway, the amvgdalar pathway, and the pathway from perirhinal cortex to the dorsomedial thalamic nucleus |
| Renou 2008 | 10.1001/archneur.65.9.1252 | 1 | 68 | M | An acute and isolated infarction of bilateral anterior columns offornices and genu of corpus callosum. |
| Philippe Rizek 2013 | 10.1017/s0317167100015031 | 1 | 56 | M | The bilateral fornix |
| Ryan 2010 | 10.1136/bcr.03.2010.2798 | 1 | 70 | M | Bilateral posterior cerebral artery (PCA) territory infarction |
| Yuko Saito 2006 | 10.1016/j.jstrokecerebrovasdis.2006.04.004 | 1 | 71 | F | The anterior fornix and genu of the corpus callosum |
| Szabo 2009 | 10.1161/STROKEAHA.108.536144 | 1 | 81 | M | Multiple acute ischemic lesions in the posterior cerebral artery territories, including the thalamus, the occipital lobes, and the hippocampus on the left and less hyperintense on the right. |
| Stenset 2007 | 10.1111/j.1600-0404.2007.00851.x | 1 | 67 | F | A left anteromedial thalamic lesion |
| Katsuaki Suzuki 2003 | 10.1016/s0920-9964(02)00386-9 | 1 | 58 | M | The bilateral hippocampi |
| SWANSON  1985 | 10.1161/01.str.16.5.823 | 1 | 27 | F | Both medial thalami |
| Takamatsu 1990 | 10.2169/internalmedicine1962.29.301 | 1 | 66 | F | Right thalamic infarction |
| Turnbull 2006 | 10.1016/j.neuropsychologia.2005.04.019 | 1 | 85 | M | Posterior cerebral artery infarction |
| Valenstein 1987 | 10.1093/brain/110.6.1631 | 1 | 39 | M | An intracerebral haemorrhage medial to the trigone of the left lateral ventricle, with extravasation into the ventricle. |
| Cramon1992 | 10.1016/s0010-9452(13)80151-7 | 1 | 25 | M | A left-sided lesion of the dorsal route involving the posterior cingulate bundle, the longitudinal striae (as part of the supracommissural hippo campus) and the fornix |
| Vucic 2003 | 10.1016/s0967-5868(02)00341-7 | 1 | 43 | M | Bilateral thalamic |
| Warren 2000 | 10.1136/jnnp.68.2.248 | 1 | 78 | F | The left anterior thalamus and periventricular white matter ischaemic changes |
| Yasuda 1997 | 10.1016/s0303-8467(97)00605-7 | 1 | 62 | M | The splenium of the corpus callosum and the retrosplenial region on the right side |
| Yuichiro Yoneoka 2004 | PMID: 15205131 | 1 | 56 | M | Multiple ischemic lesions in the midpons, the head of the right caudate nucleus, and the left anterior thalamus. |
| Abe 1998 | 10.1136/jnnp.65.1.126 | 1 | 61 | M | A discrete lesion located in the right basal forebrain including the diagonal band of Broca, anterior hypothalamus, and the lamina terminalis |
| Adamovich 2009 | 10.1212/WNL.0b013e3181bd80af | 1 | 53 | F | An acute infarction of the anterior fornices |
| Amuluru 2015 | 10.1016/j.jstrokecerebrovasdis.2015.06.038 | 1 | 50 | F | Focal restricted diffusion involving the left mammillary body |
| Bereznyakova 2014 | 10.1212/WNL.0000000000000676 | 1 | 60 | F | An acute ischemic stroke in the anterior fornices and the  anterior cingulate gyrus |
| Bird 2007 | 10.1016/j.neuropsychologia.2006.10.011 | 1 | 58 | F | Selective right hippocampal atrophy |
| Chen 2008 | PMID: 18754267 | 1 | 72 | F | Diffusion-weighted image with restricted diffusion in the region of the fornix |
| Coslett 1991 | 10.1001/archneur.1991.00530210075027 | 1 | 54 | F | The hyperdense lesion in the antenior part of the left thalamus was isodense in Tl and took up gadolinium |
| Goldenberg 1983 | 10.1007/BF00313445 | 1 | 40 | M | Ischaemic infarction in the left anterolateral thalamus |
| Grewal 2003 | 10.1016/s0967-5868(02)00278-3 | 1 | 60 | M | An infarct extending from the left temporal lobe including the hippocampal complex to the occipital lobe in the distribution of the left posterior cerebral artery |
| Hankey 1988 | 10.1161/01.str.19.6.776 | 1 | 52 | M | hemorrhage in third ventricle and frontal horn of left lateral ventricle. |
| Kim 1994 | 10.3346/jkms.1994.9.5.427 | 1 | 61 | M | Brain MRI showed a left anterior thalamic infarction with normal angiographic findings. |
| Kim 2008 | 10.1016/j.jns.2008.04.031 | 1 | 71 | M | A high signal intensity lesion in the left globus pallidus |
| Kooistra 1988 | 10.1136/jnnp.51.6.866 | 1 | 69 | M | Lucency in left posterior internal capsule. |
| Korematsu 2010 | 10.1016/j.clineuro.2009.10.016 | 1 | 52 | M | MRI revealed a small infarction at the anterior column of the left fornix. |
| Kostić 2015 | 10.2298/vsp140330015k | 1 | 61 | F | hyperintensity of the signal in the region of the hippocampus and parahippocampally on the right. |
| Koutsouraki 2009 | 10.1016/j.jns.2009.02.369 | 1 | 38 | M | Bilateral thalamic infarction involving the medial group of thalamic nuclei MRI |
| Clarke 1994 | 10.1136/jnnp.57.1.27 | 1 | 54 | M | Bilateral thalamic infarction involving the medial group of thalamic nuclei MRI |
| Lanna 2012 | 10.1016/j.jns.2012.08.004 | 1 | 54 | F | Thalamus anteromedian stroke on MRI probable MTT and IML disruptions. |
|  |  | 2 | 45 | F | Thalamus anterior stroke on MRI probable MTT disruption and clinical results suggest ILM lesion. |
|  |  | 3 | 54 | M | Thalamus dorsomedian nuclear stroke on MRI. Probable  thalamus–frontal path disruption. |
| Laplane 1981 | 10.1016/0022-510x(81)90107-6 | 1 | 70 | F | Infarct lesions of the rostral Acgs and adjacent medial frontal cortex. Atrophy in the left thalamus and absence of infarct lesion in posterior parts of the brain. |
| Lobaz 2012 | 10.1136/bcr.12.2011.5296 | 1 | 66 | M | Infarction in the posteromedial temporal lobes and occipital lobes bilaterally. |
| Maeshima 2001 | 10.1054/jocn.2000.0801 | 1 | 21 | M | Subcortical haematoma in the left cingulate isthmus |
| Maeshima 2010 | 10.1007/s10072-010-0247-z | 1 | 54 | M | MRI at stroke onset revealed a hematoma across the pons on both sides but no obvious lesions in the cerebellum or cerebrum |
| Maeshima 2014 | 10.1016/j.jstrokecerebrovasdis.2013.03.026 | 1 | 68 | M | A subcortical hematoma was evident between the right forceps occipitalis and the parietal lobe, involving the right cingulate isthmus. |
| Maeshima 2002 | 10.1054/jocn.2001.1065 | 1 | 48 | M | Postoperative CT scan showed a low density area in the left anterior thalamus |
| Male 2017 | 10.1016/j.jstrokecerebrovasdis.2016.11.115 | 1 | 53 | M | Magnetic resonance imaging revealed an isolated left mammillary body infarct sparing adjacent structures. |

Patient identification, reference and characteristics of 77 cases of brain lesions causing Insomnia and Amnesia. Abbreviations: F, female; M, male.

## Table S4. Sample and imaging characteristics of studies included in the resting-state activation analysis of somatic symptoms

| **Study** | **DOI /** **PMID** | **Number/female** | | **Age (years)** | | **Diagnosis** | **Measure** |
| --- | --- | --- | --- | --- | --- | --- | --- |
|  |  | **Somatic**  **patients** | **HC** | **Somatic**  **patients** | **HC** |  |  |
| Chen 2021 | 10.3389/fnins.2021.721822 | 36/20 | 36/26 | 34.36 ± 9.53 | 31.67 ± 8.85 | IBS | ALFF |
| Ma 2015 | 10.1016/j.clinph.2014.10.004 | 21/7 | 21/10 | 41.82 ± 11.92 | 35.91 ± 14.76 | IBS | ALFF |
| Qi 2016 | 10.1007/s11682-015-9478-1 | 30/6 | 31/7 | 28.93 ± 9.71 | 26.87 ± 8.08 | IBS | ALFF |
| Ao 2021 | 10.1186/s12880-020-00541-9 | 13/5 | 14/6 | 32.23 ± 5.96 | 29.14 ± 5.92 | IBS | ALFF |
| Jin 2019 | 10.1111/nmo.13566 | 46/34 | 53/35 | 38.9 ± 1.8 (F)  43.3 ± 3.7 (M) | 42.2 ± 2.6 (F)  38.0 ± 3.5 (M) | FC | ALFF |
| Yan 2019 | 10.3389/fpsyt.2019.00267 | 35/16 | 27/14 | 33.1 ± 8.9 | 32.3 ± 7.8 | MDD | ALFF |
| Liu 2021 | 10.1016/j.jad.2021.08.143 | 50/31 | 70/38 | 33.33 ± 9.04 | 31.76 ± 9.15 | MDD | ALFF |
| Su 2014 | 10.1371/journal.pone.0099273 | 25/21 | 28/22 | 41.00 ± 10.76 | 38.71 ± 9.59 | SD | ALFF |
| Su 2021 | 10.1016/j.jad.2020.11.090 | 25/21 | 28/22 | 41.00 ± 10.76 | 38.71 ± 9.59 | SD | ALFF |
| Fu 2021 | 10.3389/fpsyt.2021.744898 | 35/22 | 17/11 | 30.86 ± 6.84 | 30.14 ± 5.00 | MDD | ALFF |
| Fan 2023 | 10.1007/s00234-023-03237-3 | 56/28 | 56/28 | 55.32 ± 5.67 | 54.37 ± 6.64 | cLBP | ALFF |
| Liu 2015 | 10.1016/j.bbr.2014.11.047 | 61/17 | 68/34 | 36.35 ± 13.28 | 35.55 ± 12.43 | MDD | ALFF |
| Chen 2021 | 10.3389/fnins.2021.721822 | 36/20 | 36/10 | 34.36 ± 9.53 | 31.67 ± 8.85 | IBS | ReHo |
| Ke J 2015 | 10.1111/nmo.12692 | 31/6 | 32/7 | 29.2 ± 9.7 | 27.5 ± 8.6 | IBS | ReHo |
| Yu 2017 | 10.1177/0300060516679345 | 25/12 | 20/10 | 47.68 ± 10.99 | 42.50 ± 11.94 | CNSP | ReHo |
| Li 2020 | 10.2147/JPR.S220370 | 37/17 | 24/14 | 53.65 ± 5.45 | 55.83 ± 4.72 | CSP | ReHo |
| Zhang 2024 | 10.1007/s00234-024-03286-2 | 60/30 | 60/30 | 46.7 ± 15.7 | 47.2 ± 16.3 | CNSP | ReHo |
| Jin 2021 | 10.1017/neu.2021.9 | 21/7 | 24/12 | 35.43 ± 6.76 | 30.92 ± 6.26 | HS | ReHo |
| Song 2015 | 10.1016/j.ijpsycho.2015.05.012 | 25/21 | 28/22 | 41.00 ± 10.76 | 38.71 ±9.59 | SD | ReHo |
| Li 2016 | 10.1002/brb3.521 | 11/7 | 12/6 | 49.5 ± 12.1 | 49.0 ± 14.6 | SSD | ReHo |
| Geng 2019 | 10.1016/j.jad.2018.12.066 | 28/15 | 30/13 | 37.21 ± 7.16 | 35.83 ± 8.15 | SD | ReHo |
| Huang 2016 | 10.1371/journal.pone.0151360 | 13/8 | 23/11 | 46.0 ± 14.3 | 46.1 ± 12.7 | PSPD | ReHo |
| Liu 2020 | 10.1016/j.jad.2020.03.107 | 36/21 | 27/14 | 35.75 ± 9.93 | 32.44 ± 8.57 | MDD(GI) | ReHo |
| Yan 2021 | 10.3389/fpsyt.2021.636820 | 35/22 | 28/14 | 30.86 ± 6.84 | 30.86 ± 6.84 | MDD(GI) | ReHo |
| Yoshino 2014 | 10.1017/S1461145714000741 | 9/4 | 20/13 | 39.0 ± 5.1 | 42.4 ± 6.8 | SPD | ReHo |
| Ou 2018 | 10.1016/j.jad.2018.04.036 | 25/21 | 28/22 | 41.00 ± 10.76 | 38.71 ± 9.59 | SD | ReHo |
| Yoshino 2017 | 10.1038/s41598-017-03522-1 | 43/23 | 41/25 | 44.4 ± 11.0 | 45.9 ± 9.4 | MDD | ReHo |

Contrasts identification, reference and characteristics of resting-state activation of somatic patients and healthy controls. Age is expressed as mean ± standard deviation. Abbreviations: HC, healthy controls; M, male; F, female; IBS, irritable bowel syndrome; FC, functional constipation; MDD, major depressive disorder; SD, somatic disorder; cLBP, chronic low back pain; CNSP, Chronic neck and shoulder pain; CSP, Chronic shoulder pain; HS, patients with hypochondriasis; PSPD, Persistent somatoform pain disorder; GI, gastrointestinal.

## Table S5. Sample and imaging characteristics of the studies included in the gray matter volume analysis of somatic symptoms

| **Study** | **DOI /** **PMID** | **Number/female** | | **Age (years)** | | **Diagnosis** | **Measure** |
| --- | --- | --- | --- | --- | --- | --- | --- |
|  |  | **Somatic**  **patients** | **HC** | **Somatic**  **patients** | **HC** |  |  |
| Sun 2023 | 10.1016/j.jad.2022.11.023 | 342/221 | 509/291 | 34.98 ± 11.18 | 33.24 ± 11.57 | MDD (SD) | GMV |
| Bhatt 2019 | 10.1097/PSY.0000000000000655 | 32/32 | 26/26 | 11.40 ± 3.01 | 10.72 ± 2.79 | IBS | GMV |
| Öhlmann 2021 | 10.3389/fneur.2021.733035 | 23/23 | 23/23 | 46.91 ± 10.92 | 43.74 ± 12.17 | IBS | GMV |
| Schmidt-Wilcke 2010 | 10.1111/j.1526-4610.2010.01637.x | 11/9 | 11/9 | 52.2 ± 8.9 | 51.3 ± 8.6 | PIPF | GMV |
| Hans-Christian 2016 | 10.1016/j.jpain.2015.10.003 | 111/78 | 432/185 | 53.12 ± 11.77 | 48.92 ± 13.96 | CBP | GMV |
| Shen 2020 | 10.3389/fnhum.2020.596157 | 21/7 | 22/14 | 35.43 ± 6.76 | 31.64 ± 6.06 | Hypochondriasis | GMV |
| Ruscheweyh 2011 | 10.1016/j.pain.2011.01.013 | 45/29 | 31/12 | 66 ± 7 | 63 ± 9 | Pain disorder | GMV |
| Pirwani 2020 | 10.1002/jdn.10044 | 26/18 | 24/16 | 18.91 ± 1.17 | 19.08 ± 1.16 | GIS | GMV |
| Lee 2019 | 10.1111/jop.12838 | 12/NA | 14/NA | NA | NA | BMS | GMV |
| Tan 2019 | 10.3389/fpsyg.2019.01700 | 26/21 | 27/25 | 52.12 ± 8.81 | 51.11 ± 5.42 | BMS | GMV |
| Popp 2018 | 10.1002/acn3.570 | 44/24 | 44/24 | 44 ± 14 | 43 ± 14 | PPV | GMV |
| Liu 2020 | 10.1016/j.jad.2020.03.107 | 36/21 | 27/14 | 35.75 ± 9.93 | 32.44 ± 8.57 | MDD | GMV |
| Seminowicz 2011 | 10.1523/JNEUROSCI.5280-10.2011 | 18/10 | 16/8 | 46 ± 10.64 | 40 ± 13.16 | CLBP | GMV |
| Maurer 2018 | 10.1212/WNL.0000000000006514 | 48/38 | 55/40 | 44.6 ± 11.6 | 45 ± 12 | FMD | GMV |
| Riederer 2017 | 10.3109/15622975.2015.1073356 | 25/17 | 29/20 | 42.1 ± 9.9 | 42.4 ± 9.8 | NDSD | GMV |
| Valet 2009 | 10.1097/PSY.0b013e31818d1e02 | 14/14 | 25/25 | 51.1 ± 11.1 | 51.7 ± 7.2 | pain disorder | GMV |
| Huber 2018 | 10.1016/j.pnpbp.2017.11.022 | 18/10 | 20/12 | 74.3 ± 8.9 | 70.4 ± 11.9 | Delusional disorders | GMV |
| Kuchinad 2007 | 10.1523/JNEUROSCI.0098-07.2007 | 10/10 | 10/10 | 52 | 45 | FM | GMV |
| Delvecchio 2019 | 10.1016/j.psym.2018.07.005 | 20/13 | 24/17 | 45.5 ± 10.4 | 48.8 ± 15.5 | SD | GMV |
| Liu 2019 | 10.2147/NDT.S197351 | 24/15 | 30/14 | 34.79 ± 10.71 | 33.43 ± 8.98 | MDD | GMV |
| Liu 2021 | 10.1016/j.pnpbp.2021.110386 | 335/219 | 446/252 | 34.35 ± 11.30 | 33.15 ± 11.56 | MDD | GMV |
| Mo 2023 | 10.3389/fpsyt.2023.1197854 | 34/26 | 19/15 | 14.97 ± 1.54 | 15.91 ± 1.92 | MDD | GMV |
| Finkelmeyer 2018 | 10.1016/j.nicl.2017.09.024 | 42/32 | 28/19 | 45.6 ± 11.7 | 48.4 ± 11.3 | CFS | GMV |
| Kozlowska 2017 | 10.1016/j.nicl.2017.04.028 | 25/20 | 24/28 | 14.57 ± 2.03 | 14.34 ± 2.75 | FND | GMV |
| Wei 2015 | 10.1093/scan/nsu033 | NA | 288/134 | NA | 19.94 ± 1.32 | NA | GMV |
| Schmidt-Wilcke 2007 | 10.1016/j.pain.2007.05.010 | 20/19 | 22/20 | 53.6 ± 7.7 | 50.7 ± 7.3 | FM | GMV |

Contrasts identification, reference and characteristics of GMV analysis of somatic patients and healthy controls. Age is expressed as mean ± standard deviation. Abbreviations: MDD, major depressive disorder; GMV, gray matter volume; SD, somatic depression; IBS, irritable bowel syndrome; PIFP, persistent idiopathic facial pain; NA, not applicable; CBP, Chronic back pain; GIS, Gastrointestinal Syndrome; BMS, burning mouth syndrome; PPV, phobic postural vertigo; CLBP, chronic low back pain; FMD, functional movement disorder; NDSD, nondermatomal sensory deficits; CFS, Chronic Fatigue Syndrome; FND, functional neurological disorder; FM, Fibromyalgia.

## Table S6. Sample and imaging characteristics of the studies included in the task-induced activation analysis of somatic symptoms

| **Study** | **DOI /** **PMID** | **Number/female** | | **Age (years)** | | **Diagnosis** | **Paradigm** |
| --- | --- | --- | --- | --- | --- | --- | --- |
|  |  | **Somatic**  **patients** | **HC** | **Somatic**  **patients** | **HC** |  |  |
| Gündel 2008 | 10.1016/j.pain.2007.10.003 | 12/12 | 13/NA | 47.4 ± 8.2 | 47.3 ± 9.3 | Somatoform pain disorder | Pain stimulation protocol |
| Bogaerts 2023 | 10.1038/s41398-023-02567-3 | 30/30 | 30/30 | 43.3 ± 11.34 | 42.97 ± 11.81 | FSS | Watch neutral, positive and negative affective picture blocks |
| Glass 2011 | 10.1016/j.jpain.2011.06.007 | 18/18 | 14/14 | 43.6 ± 9.79 | 41.13 ± 11.91 | FM | Go/NoGo Task |
| Gracely 2002 | 10.1002/art.10225 | 16/15 | 16/15 | 52.6 ± 12.3 | 45.8 ± 10.5 | FM | Subjective pain control condition |
| Landgrebe 2008 | 10.1016/j.neuroimage.2008.04.171 | 15/6 | 15/6 | 47.7 ± 10.5 | 46.9 ± 9.9 | Subjective electro-hypersensitivity | Exposed to sham mobile phone radiation and heat as a control condition |
| Huang 2016 | 10.1097/MPG.0000000000001390 | 10/8 | 10/6 | 17 ± NA | 20 ± NA | IBS | Anticipated and thermal pain stimuli |
| Yan 2017 | 10.1007/s00406-017-0832-8 | 32/19 | 35/19 | 40.28 ± 12.11 | 41.63 ± 13.72 | PHA | The body‑symptom implicit association task |
| Greck 2012 | 10.1002/hbm.21392 | 20/NA | 20/NA | NA | NA | Somatoform disorder | Monetary-Incentive-Delay task |
| Greck 2013 | 10.3389/fnhum.2013.00410 | 20/12 | 20/12 | 42.5 ± 14.0 | 37.0 ± 13.6 | Somatoform disorder | Florida Affect Battery |
| Song 2006 | 10.1016/j.pain.2006.06.017 | 12/NA | 12/NA | 23 ± 0.39 | 23 ± 0.92 | IBS | Rectal balloon distension sensory pressure thresholds |
| Yoshino 2013 | 10.1016/j.nicl.2013.06.001 | 11/6 | 11/6 | 49 ± 6.5 | 40.6 ± 6.1 | Somatoform pain disorder | A 2 × 2 block design with variables of pain stimulation and emotional context |
| Stoeter 2007 | 10.1016/j.neuroimage.2007.01.052 | 17/11 | 17/11 | 44.7 ± 9.1 | 45.4 ± 9.2 | Somatoform pain disorder | Pin-prick pain;  Cognitive stress task |
| Luo 2016 | 10.1111/papr.12358 | 12/5 | 10/4 | 45.83 ± 14.95 | 35.2 ± 10.78 | PSPD | Percutaneous nerve electrical stimulation |

Contrasts identification, reference and characteristics of task-induced activation of somatic patients and healthy controls. Age is expressed as mean ± standard deviation. Abbreviations: FSS, Functional somatic syndromes; FM, fibromyalgia; IBS, irritable bowel syndrome; PHA, pathological health anxiety; PSPD, Persistent Somatoform Pain Disorder.

## Table S7. Sample and imaging characteristics of the studies included in structure, resting-state activation or task-induced activation analysis of control symptoms

| **Study** | **DOI /** **PMID** | **Number/female** | | **Age (years)** | | **Diagnosis** | **Measure or Task** |
| --- | --- | --- | --- | --- | --- | --- | --- |
|  |  | **Somatic**  **patients** | **HC** | **Somatic**  **patients** | **HC** |  |  |
| *Gambling disorder* | | | | | | | |
| Balodis 2012 | 10.1016/j.biopsych.2012.01.006 | 14/4 | 14/4 | 35.8 ± 11.7 | 37.1 ± 11.3 | PG | MIDT |
| Brevers 2017 | 10.1016/j.biopsycho.2017.01.009 | 17/6 | 16/10 | 28.91 ± NA | 26.87 | high-frequency poker players | Modified version of the stop-signal paradigm |
| Brevers 2016 | 10.1111/adb.12239 | 15/9 | 15/9 | 24.67 ± 5.32 | 22.07 ± 1.67 | frequent poker players | IGT |
| Cerasa 2014 | 10.1016/j.parkreldis.2014.09.001 | 12/1 | 24/4 | 58 ± 8.3 | 60.3 ± 9.1 | PD with PG | GMV |
| Chase 2010 | 10.1523/jneurosci.5758-09.2010 | 20/2 | 20/2 | 33.7 ± 1.8 | NA | Regular gamblers | Simplified slot machine task |
| Crockford 2005 | 10.1016/j.biopsych.2005.04.037 | 10/0 | 10/0 | 39.3 ± 7.6 | 39.2 ± 8.3 | PG | Visual presentations of gambling-related video alternating with video of nature scenes |
| Dannon 2011 | 10.1007/s11682-010-9109-9 | 10/0 | NA | NA | NA | PG | Alternation learning task |
| de Ruiter 2009 | 10.1038/npp.2008.175 | 19/0 | 19/0 | 34.3 ± 9.4 | 34.1 ± 9.3 | PG | Probabilistic reversal-learning task |
| Fauth-Bühler 2014 | 10.1016/j.nicl.2014.09.008 | 80/0 | 89/0 | 37.4 ± 9.1 | 36.2 ± 9.4 | GD | Effort-dependent task |
| Freinhofer 2020 | 10.3389/fpsyt.2020.00109 | 27/4 | 22/2 | 43.89 ± 11.89 | 40.77 ± 14.29 | PG | The Cups task |
| Fuentes 2015 | 10.1016/j.pscychresns.2015.04.001 | 30/0 | 30/0 | 37.3 ± 9.6 | 37.3 ± 9.7 | PG | GMV |
| Gelskov 2016 | 10.1016/j.neuroimage.2016.01.002 | 14/0 | 15/0 | 29.43 ± 6.05 | 29.87 ± 6.06 | PG | Mixed gain/loss gambles |
| Genauck 2017 | 10.1038/s41598-017-16433-y | 19/0 | 17/0 | 32.9 ± 10 | 38.8 ± 11.5 | PG | Loss aversion task |
| Goudriaan 2010 | 10.1111/j.1369-1600.2010.00242.x | 17/0 | 17/0 | 35.3 ± 9.4 | 34.7 ± 9.7 | PRG | Watching gambling pictures |
| Joutsa 2011 | 10.1016/j.pscychresns.2011.08.001 | 12/0 | 12/0 | 30.0 ± NA | 27.0 ± NA | PG | GMV |
| Limbrick-Oldfield 2017 | 10.1038/tp.2016.256 | 20/0 | 22/0 | 31 ± NA | 28 ± NA | GD | Cue reactivity procedure |
| Miedl 2012 | 10.1001/archgenpsychiatry.2011.1552 | 16/1 | 16/1 | 35 ± 2 | 38 ± 2 | PG | DD and PD tasks |
| Mohammadi 2016 | 10.1007/s00429-015-1093-9 | 15/0 | 15/0 | 36.7 ± 5.8 | 36.8 ± 5.6 | PG | GMV |
| Power 2012 | 10.1007/s10899-011-9278-5 | 13/0 | 13/0 | 42.4 ± 10.8 | 41.0 ± 11.0 | PG | IGT |
| Sescousse 2013 | 10.1523/jneurosci.5828-12.2013 | 18/0 | 20/0 | 34.1 ± 11.6 | 31 ± 7.3 | PG | A simple incentive task |
| Sescousse 2016 | 10.1038/npp.2016.43 | 22/0 | 22/0 | 35.7 ± 8.8 | 32.2 ± 11.1 | PG | A slot machine task |
| Tanabe 2007 | 10.1002/hbm.20344 | 14/8 | 18/11 | 35 ± 7 | 37 ± 9 | PG | IGT |
| Tsurumi 2014 | 10.3389/fpsyg.2014.01013 | 23/0 | 27/0 | 32.6 ± 6.9 | 33.4 ± 8.0 | PG | MIDT |
| van Holst 2012 | 10.1016/j.drugalcdep.2011.12.025 | 40/0 | 54/0 | 36.5 ± 10.67 | 35.3 ± 10.1 | PRG | GMV |
| Worhunsky 2014 | 10.1016/j.drugalcdep.2014.09.013 | 24/9 | 24/11 | 38.2 ± 10.9 | 33.4 ± 10.5 | PG | A slot-machine task |
| Yip 2018 | 10.1111/adb.12492 | 26/7 | 28/7 | 38.40 ± 11.80 | 38.00 ± 11.03 | PG | GMV |
| Zois 2017 | 10.1111/adb.12368 | 107/0 | 98/0 | 36.7 ± 8.9 | 36.1 ± 9.7 | PG | GMV |
| *Insomnia* | | | | | | | |
| Altena 2013 | 10.1016/j.biopsych.2009.08.003 | 24/17 | 13/9 | 60.3 ± 6.0 | 60.2 ± 8.4 | Insomnia | GMV |
| Chou 2021 | 10.1093/sleep/zsaa202 | 22/15 | 27/21 | 40.2 ± 11.7 | 41.3 ± 10.1 | Insomnia | GMV |
| Grau-Rivera 2020 | 10.1186/s13195-019-0547-3 | 137/87 | 229/117 | 56.6 ± 6.8 | 55.4 ± 6.5 | Insomnia | GMV |
| Joo 2013 | 10.5665/sleep.2796 | 27/25 | 27/23 | 52.3 ± 7.8 | 51.7 ± 5.4 | PI | GMV |
| Li 2018 | 10.1007/s11682-018-9844-x | 60/32 | 53/35 | 39.90 ± 10.75 | 39.55 ± 8.77 | PI | GMV |
| Li 2018 | 10.1097/md.0000000000011944 | 15/9 | 15/8 | 37.13 ± 2.530 | 32.60 ± 2.54 | PI | GMV |
| Li 2018 | 10.1007/s00330-017-5012-8 | 59/32 | 53/32 | 39.78 ± 10.63 | 39.70 ± 8.84 | PI | FC |
| *Aphasia* | | | | | | | |
| Crinion 2005 | 10.1093/brain/awh659 | 17/5 | 18/10 | 62 ± 2.7 | 58 ± 2.8^a^ | Aphasia | Auditory repetition tasks |
| Fridriksson 2012 | 10.1093/brain/aws301 | 13/4 | 20/14 | 53 ± 7 | 53 ± 7 | Broca’s aphasia | Three separate behavioural tasks |
| Lorca-Puls 2021 | 10.1093/braincomms/fcab230 | 7/3 | 55/33 | 63.3 6 ± 13.4 | 43.46 ± 17.6 | Broca’s aphasia | 13 different tasks |
| Qiu 2017 | 10.4103/1673-5374.198996 | 10/3 | 10/3 | 55.89 ± 13.37 | 55.89 ± 11.78 | Broca’s aphasia | Picture naming task |
| Saur 2006 | 10.1093/brain/awl090 | 14/3 | 14/3 | 51.9 ± 14.2 | 48.6 ± 13.9 | Aphasia | Auditory comprehension task |
| Skipper-Kallal 2017 | 10.1155/2017/8740353 | 39/13 | 37/17 | 59.8 ± 10.1 | 58.7 ± 13.2 | Aphasia | Picture naming task |
| Szaflarski 2011 | 10.1016/j.jstrokecerebrovasdis.2010.02.003 | 4/2 | 4/2 | 53.2 ± 3.9 | 46.0 ± 15.2 | Aphasia | Picture identification task |
| *Suicide* | | | | | | | |
| Aguilar 2008 | 10.1016/j.pnpbp.2008.06.016 | 13/0 | 24/0 | 37.12 ± 10.02 | 42.65 ± 10.19 | Schizophrenia | GMV |
| Benedetti 2011 | 10.1016/j.jad.2011.07.006 | 38/28 | 19/10 | NA | NA | BD | GMV |
| Canal-Rivero 2020 | 10.1016/j.pnpbp.2020.109948 | 23/9 | 122/46 | 28.41 ± 7.68 | 29.39 ± 8.29 | FEP | GMV |
| Ding 2015 | 10.1038/tp.2015.1 | 67/39 | 82/20 | 39.2 ± 10.6 | 37.8 ± 8.1 | Mood disorder | GMV |
| Duarte 2017 | 10.1016/j.pscychresns.2017.04.012 | 20/12 | 20/11 | 41.10 ± 12.64 | 37.40 ± 10.20 | BD | GMV |
| Fan 2019 | 10.1016/j.jad.2018.11.095 | 21/18 | 25/21 | 20.3 ± 3 | 20.2 ± 3.4 | BD | GMV |
|  |  | 19/16 | 18/15 | 18.7 ± 3 | 18.9 ± 2.7 | MDD | GMV |
| Guo 2021 | 10.3389/fpsyt.2021.630849 | 30/30 | 75/75 | 25.77 ± 9.18 | 30.54 11.55 | BD | GMV |
| Hwang 2010 | 10.1177/0891988710363713 | 70/0 | 26/0 | 79.1 ± 5.6 | 79.6 ± 5.1 | MDD | GMV |
| Jabbi 2020 | 10.1016/j.psychres.2020.113516 | 11/8 | 40/21 | 16.1 ± 1.53 | 22.45 ± 5.44 | BD | GMV |
| Johnston 2017 | 10.1176/appi.ajp.2016.15050652 | 26/20 | 42/23 | 20.5 ± 3.0 | 20.6 ± 3.2 | BD | GMV |
| Jollant 2018 | 10.1038/s41398-018-0170-2 | 17/14 | 66/36 | 32.3 ± 7.4 | 37.6 ± 10.2 | MDD | GMV |
| Lee 2016 | 10.1016/j.comppsych.2016.02.013 | 19/11 | 19/9 | 41.95 ± 10.81 | 41.11 ± 15.15 | MDD | GMV |
| Lippard 2019 | 10.1016/j.jad.2018.11.097 | 29/16 | 17/12 | 18.1 ± 2.7 | 18.2 ± 3.4 | Mood disorder | GMV |
| Peng 2014 | 10.1016/j.jad.2014.04.046 | 20/13 | 28/13 | 27.75 ± 7.21 | 28.61 ± 5.45 | MDD | GMV |
| Shao 2022 | 10.1016/j.bpsc.2021.11.011 | 35/33 | 45/32 | 64.74 ± 5.22 | 67.73 ± 5.68 | LLD | GMV |
| Soloff 2012 | 10.1016/j.jpsychires.2012.01.003 | 68/52 | 52/24 | 28.3 ± 7.5 | 25.9 ± 7.2 | BPD | GMV |
| Wang 2020 | 10.1186/s12991-020-00318-y | 36/29 | 34/26 | 24.58 ± 8.19 | 30.65 ± 10.14 | Mood disorder | GMV |

Contrasts identification, reference and characteristics of brain damage of patients with control symptoms and healthy individuals. Age is expressed as mean ± standard deviation. Abbreviations: PG, pathological gambling; MIDT, Monetary Incentive Delay Task; IGT, Iowa Gambling Task; GD, Gambling disorder; PRG, problem gamblers; PI, primary insomnia; BD, bipolar disorder; FEP, first-episode psychosis; LLD, late-life depression; BPD, Borderline Personality Disorder.

## Table S8. Sample and imaging characteristics of the studies included in the task-induced activation analysis of interoceptive processing

| **Study** | **DOI /** **PMID** | **Number/F** | **Age (years)** | **Scanner** | **Paradigm** |
| --- | --- | --- | --- | --- | --- |
| Tracy 2007 | 10.1097/PSY.0b013e31815b60cf | 17/11 | 94.7 ± 8.4 | 1.5 T MRI | heartbeat > tone |
| Pollatos 2007 | 10.1002/hbm.20258 | 20/0 | 26.8 ± 3.7 | 1.5 T MRI | heartbeat > tone |
| Kuehn 2016 | 10.1007/s00429-015-0989-8 | 23/9 | NA | 3.0 T MRI | heartbeat > tone |
| Zaki 2012 | 10.1016/j.neuroimage.2012.05.012 | 16/11 | 19.10 ± 1.72 | 1.5 T MRI | heartbeat > tone |
| Caseras 2013 | 10.1002/hbm.21503 | 18/16 | 21.72 ± 2.80 | 1.5 T MRI | heartbeat > tone |
| Stern 2017 | 10.1002/hbm.23811 | 19/NA | NA | 3.0 TMRI | heartbeat > tone |
| Blefari 2017 | 10.1111/ejn.13567 | 16/5 | 24.5 ± 2.7 | 7.0 T MRI | heartbeat > color |
| Sclocco 2016 | 10.1098/rsta.2015.0189 | 11/3 | 33 ± 4 | 7.0 T MRI | covariation of HF-HRV over tasks |
| Napadow 2008 | 10.1016/j.neuroimage.2008.04.238 | 7/3 | NA | 3.0 T MRI | covariation of HF-HRV and BOLD |
| Wager 2009 | 10.1016/j.neuroimage.2009.05.044 | 18/9 | 21 ± NA | 1.5 T MRI | correlation of HR and BOLD |
| Napadow 2013 | 10.1002/hbm.22091 | 18/11 | 27.9 ± 11.0 | 3.0 T MRI | correlation of HR and BOLD |
| Simmons 2013 | 10.1155/2013/632402 | 14/8 | 29 ± NA | 3.0 T MRI | Heart & stomach > rest |
| Führer 2008 | 10.1038/oby.2008.33 | 12/0 | 26.42 ± NA | 3.0 T MRI | food > nonfood |
| Martens 2013 | 10.3945/ajcn.112.044024 | 20/10 | 22.4 ± 0.5 | 3.0 T MRI | food > nonfood |
| Grimm 2012 | 10.1016/j.appet.2012.02.007 | 27/14 | 26.0 ± 3.3 | 3.0 T MRI | food > nonfood |
| Kaurijoki 2008 | 10.1111/j.1475-097X.2008.00804.x | 28/16 | 25 ± NA | 1.5 T MRI | food > nonfood |
| Kim 2012 | 10.1016/j.neulet.2012.05.075 | 20/20 | 23.3 ± 1.8 | 1.5 T MRI | food > nonfood |
| Malik 2011 | 10.1016/j.appet.2011.01.005 | 10/0 | 25.8 ± 0.8 | 1.5 T MRI | food > nonfood |
| Haase 2011 | 10.1016/j.appet.2011.06.009 | 21/12 | 22.25 ± 2.7 (M)  21.94 ± 1.9 (F) | 3.0 T MRI | food > nonfood |
| Eiler 2012 | 10.1007/s12078-011-9112-6 | 11/11 | 23.5 ± 4.4 | 3.0 T MRI | food > nonfood |
| Uher 2006 | 10.1016/j.bbr.2005.12.008 | 18/10 | 28.4 ± 8.4 (M)  29.4 ± 7.8 (F) | 1.5 T MRI | food > nonfood |
| Yan 2011 | 10.1007/s00221-010-2516-8 | 19/NA | NA | 3.0 T MRI | food > nonfood |
| Gizewski 2010 | 10.1159/000319360 | 12/12 | 25.3 ± NA | 1.5 T MRI | food > nonfood |
| Yousuf 2018 | 10.1007/s11682-017-9811-y | 20/0 | 23.65 ± 3.31 | 3.0 T MRI | food > money |
| Frank 2010 | 10.1016/j.brainres.2010.04.030 | 12/6 | 27.42 ± 4.14 | 3.0 T MRI | high calorie > low calorie |
| Filbey 2012 | 10.1016/j.neuroimage.2012.08.073 | 26/14 | 32.88 ± 11.04 | 3.0 T MRI | high calorie > low calorie |
| Bragulat 2010 | 10.1038/oby.2010.57 | 5/5 | 23.4 ± 1.1 | 3.0 T MRI | appetitive > non-appetitive |
| Farr 2018 | 10.1111/dom.13060 | 9/3 | NA | 3.0 T MRI | appetitive > non-appetitive |
| Haase 2009 | 10.1016/j.neuroimage.2008.09.044 | 18/9 | 20.7 ± 0.99 | 3.0 T MRI | hunger > satiety |
| Green 2015 | 10.1016/j.brainres.2015.03.034 | 15/8 | 48.7 ± 0.8 | 3.0 T MRI | hunger > satiety |
| Tataranni 1999 | 10.1073/pnas.96.8.4569 | 11/0 | 35 ± 8 | 1.5 T MRI | hunger > satiety |
| Farrell 2011 | 10.1152/ajpregu.00817.2010 | 15/4 | 23 ± NA | 3.0 T MRI | thirst > baseline |
| Becker 2015 | 10.1093/scan/nsv063 | 24/12 | 22.9 ± NA | 1.5 T MRI | beverage > chair |
| Lowell 2008 | 10.1016/j.neuroimage.2008.04.234 | 14/7 | 36 ± 10.4 | 3.0 T MRI | Dyspnea > baseline |
| Stoeckel 2015 | 10.3389/fpsyg.2015.00748 | 46/18 | 28.5 ± NA | 3.0 T MRI | Dyspnea > baseline |
| Chan 2019 | 10.1038/s41598-019-51396-2 | 34/20 | 23 ± 3.4 | 3.0 T MRI | Dyspnea > baseline |
| Ladabaum 2007 | 10.1016/j.neuroimage.2006.07.033 | 18/4 | 32 ± 6.5 | 1.5 T MRI | Gastric filling > baseline |
| Wang 2008 | 10.1016/j.neuroimage.2007.11.008 | 15/0 | 32.0 ± 6.6 | 4.0 T MRI | Gastric filling > baseline |
| Ly 2017 | 10.1016/j.neuroimage.2016.09.032 | 15/7 | 27.3 ± 2.1 | 3.0 T MRI | Gastric filling > baseline |
| Kuhtz-Buschbeck 2005 | 10.1097/01.ju.0000173007.84102.7c | 22/22 | 24.5 ± 2.8 | 1.5 T MRI | Micturition > baseline |
| Nour 2000 | 10.1093/brain/123.4.781 | 12/0 | 23.4 ± 1.1 | 1.5 T MRI | Micturition > baseline |
| Seseke 2006 | 10.1016/j.neuroimage.2006.02.012 | 11/11 | 30.0 ± 6.9 | 3.0 T MRI | Micturition > baseline |
| Zhang 2005 | 10.1016/j.neuroimage.2004.08.027 | 12/0 | 23.8 ± 0.65 | 3.0 T MRI | Micturition > baseline |
| Mehnert 2008 | 10.1016/j.neuroimage.2008.03.006 | 8/8 | 24.3 ± NA | 3.0 T MRI | Micturition > baseline |
| Seseke 2008 | 10.1016/j.neuroimage.2008.07.044 | 12/0 | 32.4 ± 7.9 | 3.0 T MRI | Micturition > baseline |
| Athwal 2001 | 10.1093/brain/124.2.369 | 11/0 | NA | NA | Micturition > baseline |
| Kuhtz-Buschbeck 2007 | 10.1016/j.neuroimage.2006.12.032 | 30/15 | 26.6 ± 2.8 | 1.5 T MRI | Micturition > baseline |
| Tadic 2008 | 10.1016/j.neuroimage.2007.10.059 | 24/24 | NA | 3.0 T MRI | Micturition > baseline |
| Griffiths 2007 | 10.1016/j.neuroimage.2007.04.061 | 10/10 | 56.5 ± NA | 3.0 T MRI | Micturition > baseline |
| Kuhtz-Buschbeck 2009 | 10.1016/j.neuroimage.2009.04.020 | 33/17 | 26.4 ± 4.2 | 1.5 T MRI | Micturition > baseline |
| Mehnert 2011 | 10.1002/hbm.21070 | 14/14 | 24.8 ± NA | 1.8 T MRI | Micturition > baseline |
| Michels 2015 | 10.1093/cercor/bhu140 | 22/0 | 26.4 ± 6.2 | 3.0 T MRI | Micturition > baseline |
| Gao 2015 | 10.1007/s11255-015-0978-0 | 30/22 | 29.8 ± 5.9 | 3.0 T MRI | Micturition > baseline |
| Walter 2019 | 10.1016/j.neuroimage.2019.02.031 | 20/10 | 39.2 ± 11.6 | 3.0 T MRI | Micturition > baseline |
| Kleint 2015 | 10.1371/journal.pone.0133164 | 36/26 | 22.3 ± 3.1 | 3.0 T MRI | heartbeat > tone |
| Ernst 2012 | 10.1002/hbm.22014 | 18/12 | 27 ± 7.6 | 3.0 T MRI | heartbeat > tone |
| Garfinkel 2014 | 10.1523/jneurosci.3507-13.2014 | 23/15 | 26.7 ± 8.6 | 1.5 T MRI | diastole > systole |
| Kroemer 2012 | 10.1111/j.1369-1600.2012.00489.x | 26/13 | 24.4 ± 3.4 | 3.0 T MRI | food > nonfood |
| Santel 2006 | 10.1016/j.brainres.2006.07.045 | 10/10 | 16.8 ± 2.6 | 1.5 T MRI | food > nonfood |
| Piech 2009 | 10.1371/journal.pone.0006581 | 8/3 | 27.9 ± 4.1 | 1.5 T MRI | food > nonfood |
| Burger 2016 | 10.3945/jn.116.231431 | 44/44 | 20.8 ± 1.3 | 3.0 T MRI | food > nonfood |
| Wang 2016 | 10.3389/fpsyg.2016.00152 | 12/12 | 20.92 ± 1.5 | 3.0 T MRI | food > nonfood |
| Blechert 2016 | 10.1016/j.appet.2016.01.006 | 32/16 | 22.4 ± 2.41 | 3.0 T MRI | high calorie > low calorie |
| Egan 2003 | 10.1073/pnas.2136650100 | 4/1 | 26 ± NA | 3.0 T MRI | thirst > baseline |
| Saker 2018 | 10.1073/pnas.1717646115 | 20/7 | 30.0 ± 1.5 | 3.0 T MRI | thirst > satiety |
| Lu 2004 | 10.1111/j.1365-2982.2004.00562.x | 10/2 | 23.6 ± NA | 3.0 T MRI | Gastric filling > baseline |
| Camps 2018 | 10.1016/j.appet.2018.04.023 | 19/0 | 22.2 ± 2.5 | 3.0 T MRI | Gastric filling > baseline |
| Griffiths 2005 | 10.1097/01.ju.0000177450.34451.97 | 12/11 | 43.5 ± NA | 3.0 T MRI | Micturition > baseline pet |
| Krhut 2012 | 10.1016/j.juro.2012.04.004 | 12/12 | 49.6 ± 17.0 | 3.0 T MRI | Micturition > baseline |

Identification, reference and characteristics of interoceptive processing. Age is expressed as mean ± standard deviation. Abbreviations: NA, not applicable; MRI, magnetic resonance imaging; HF-HRV, high-frequency heart rate variability; HR, heart rate.

## Table S9. Sample and imaging characteristics of the studies included in the task-induced activation analysis of control behavioral processing

| **Study** | **DOI /** **PMID** | **Number/F** | **Age (years)** | **Scanner** | **Paradigm** |
| --- | --- | --- | --- | --- | --- |
| *Future Thinking* | | | | | |
| Addis 2007 | 10.1016/j.neuropsychologia.2006.10.016 | 16/9 | 23 ± NA | 3T MRI | Future > past |
| Botzung 2008 | 10.1016/j.bandc.2007.07.011 | 10/5 | 34.5 ± 5.14 | 2T MRI | Future > control |
| Abraham 2008 | 10.1016/j.brainres.2008.07.084 | 20/10 | 26 ± NA | 3T MRI | Future > past |
| Weiler 2010a | 10.1002/hipo.20695 | 17/11 | 24 ± 2.6 | 1.5T MRI | Future > past |
| Weiler 2010b | 10.1016/j.bbr.2010.04.013 | 32/20 | NA | 1.5T MRI | Future > control |
| Nyberg 2010 | 10.1073/pnas.1016823108 | 5/5 | 27.6 ± NA | 3T MRI | Future + past > past |
| D'Argembeau 2010 | 10.1162/jocn.2009.21314 | 20/10 | 23 ± NA | 3T MRI | Novel > repeat |
| Martin 2011 | 10.1073/pnas.1105816108 | 29/12 | (18-35) | 3T MRI | Future > control |
| Van Mulukom 2013 | 10.1371/journal.pone.0069596 | 20/11 | (18-30) | 1.5T MRI | Future > control |
| Van Hoeck 2013 | 10.1093/scan/nss031 | 14/8 | 21 ± 1.83 | 3T MRI | Future > past |
| Gilmore 2014 | 10.1093/cercor/bhu223 | 22/16 | 24.1 ± NA | 3T MRI | Future > past |
| Sasse 2015 | 10.1002/hbm.22912 | 23/11 | 24.96 ± 2.79 | 3T MRI | Future > control |
| Cascio 2016 | 10.1093/scan/nsv136 | 67/41 | 33.42 ± 13.04 | 3T MRI | Future > past |
| Kruschwitz 2018 | 10.3389/fnbeh.2018.00297 | 31/16 | 25.9 ± 3.34 | 3T MRI | Future > present |
| Palombo 2016 | 10.1093/cercor/bhw381 | 27/17 | 19.6 ± 3.4 | 3T MRI | Future > present |
| Beaty 2018 | 10.1162/jocn_a_01327 | 29/12 | 21.79 ± NA | 3T MRI | Future > control |
| *Prospective memory* | | | | | |
| Okuda 1998 | 10.1016/s0304-3940(98)00628-4 | 6/0 | 21.5 ± NA | PET | PM + Ongoing task block > Ongoing task block |
| Den ouden 2005 | 10.1016/j.neuroimage.2005.05.001 | 11/11 | (21-37) | 1.5T MRI | PM + Ongoing task block > Ongoing task block |
| Simons 2006 | 10.1016/j.neuropsychologia.2006.01.005 | 16/8 | 23.4 ± NA | 3T MRI | PM + Ongoing task block > Ongoing task block |
| Eschen 2007 | 10.1016/j.ijpsycho.2006.09.005 | 10/6 | 24.1 ± 4.10 | 3T MRI | Encoding of the intention |
| Poppenk 2010 | 10.1016/j.neuroimage.2009.08.049 | 30/9 | 25.8 ± NA | 3T MRI | Encoding of the intention |
| Okuda 2011 | 10.1016/j.neuropsychologia.2011.02.005 | 16/10 | 27.8 ± 6.6 | 3T MRI | PM + Ongoing task block > Ongoing task block |
| Gilbert 2012 | 10.1162/jocn_a_00094 | 32/14 | 25 ± NA | 1.5T MRI | Encoding of the intention |
| Benoit 2012 | 10.1093/cercor/bhr264 | 19/9 | 22.31 ± NA | 3T MRI | PM + Ongoing task block > Ongoing task block |
| McDaniel 2013 | 10.1177/0956797613481233 | 45/NA | (18-37) | 3T MRI | PM + Ongoing task block > Ongoing task block |
| Beck 2014 | 10.1016/j.neuropsychologia.2013.10.020 | 47/27 | 56.55 ± 4.31 | 3T MRI | PM + Ongoing task block > Ongoing task block |
| Wang 2014 | 10.1037/neu0000035 | 22/12 | 19.68 ± 0.72 | 3T MRI | PM + Ongoing task block > Ongoing task block |
| Gao 2014 | 10.1371/journal.pone.0090307 | 13/7 | 27.1 ± 3.6 | 3T MRI | PM + Ongoing task block > Ongoing task block |
| Peira 2016 | 10.1016/j.neuroimage.2015.10.075 | 15/6 | 22.4 ± 1.8 | 3T MRI | PM + Ongoing task block > Ongoing task block |
| *Semantic processing* | | | | | |
| Kotz 2002 | 10.1006/nimg.2002.1316 | 13/7 | 23.5 ± NA | 3T MRI | Taxonomic vs thematic relation |
| Sachs 2008 | 10.1016/j.brainres.2008.03.045 | 16/0 | 27 ± 4.7 | 3T MRI | Taxonomic vs thematic relation |
| Sachs 2011 | 10.1162/jocn.2010.21483 | 16/0 | 27 ± 4.7 | 3T MRI | Taxonomic vs thematic relation |
| Abel 2009 | 10.1016/j.neuroimage.2008.10.018 | 19/15 | 26 ± NA | 3T MRI | Taxonomic vs thematic relation |
| Sass 2009 | 10.1016/j.brainres.2009.03.013 | 16/0 | 26 ± 3.0 | 3T MRI | Taxonomic vs thematic relation |
| De Zubicaray 2014 | 10.1016/j.cortex.2014.01.018 | 28/19 | 22.93 ± NA | 3T MRI | Taxonomic vs thematic relation |
| De Zubicaray 2013 | 10.1037/a0028717 | 20/18 | 21.25 ± NA | 4T MRI | Taxonomic vs thematic relation |
| Lee 2014 | 10.1016/j.cortex.2014.04.013 | 18/8 | (20-35) | 3T MRI | Taxonomic vs thematic relation |
| Jackson 2015 | 10.1093/cercor/bhv003 | 25/16 | 25.48 ± 6.49 | 3T MRI | Taxonomic vs thematic relation |
| Davey 2016 | 10.1016/j.neuroimage.2016.05.051 | 22/13 | 24.8 ± 3.8 | 3T MRI | Taxonomic vs thematic relation |
| Zhang 2021 | 10.1016/j.neuroimage.2020.117405 | 32/27 | 20.6 ± 1.52 | 3T MRI | Taxonomic vs thematic relation |

Identification, reference and characteristics of control behavioral processing. Age is expressed as mean ± standard deviation or range of age. Abbreviations: NA, not applicable; PM, Prospective memory.

## Table S10. Gene networks showing similar spatial similarity with the somatic network

| **Gene names** | **R values** | ***P* (FDR corrected)** |  | **Gene names** | **R values** | ***P* (FDR corrected)** |
| --- | --- | --- | --- | --- | --- | --- |
| GMPPB | -0.8038 | 0.001 |  | FURIN | -0.8719 | 0.001 |
| CELSR3 | -0.7867 | 0.001 |  | TFR2 | 0.8122 | 0.001 |
| SEMA3B | 0.6648 | 0.001 |  | EIF2AK1 | -0.5964 | 0.001 |
| VPS33B | -0.7166 | 0.001 |  | HRH4 | 0.6723 | 0.001 |
| FUBP1 | -0.7506 | 0.001 |  | NAPEPLD | -0.8463 | 0.001 |
| C6orf106 | -0.7513 | 0.001 |  | NFE2L2 | 0.7273 | 0.001 |
| SDCCAG8 | 0.5875 | 0.001 |  | MCPH1 | 0.7005 | 0.001 |
| SUOX | -0.771 | 0.001 |  | TBK1 | -0.813 | 0.001 |
| MST1 | 0.6100 | 0.001 |  | JAK2 | 0.7046 | 0.001 |
| RNF123 | -0.6836 | 0.001 |  | C2CD4A | 0.8473 | 0.001 |
| MRPS21 | -0.5058 | 0.001 |  | HTR2A | -0.8541 | 0.001 |
| P4HTM | -0.5455 | 0.001 |  | ADRA2A | -0.7592 | 0.001 |
| MON1B | 0.6874 | 0.001 |  | CCK | -0.6059 | 0.001 |
| PTK2 | 0.5616 | 0.001 |  | MYO9B | 0.6765 | 0.001 |
| SLC25A13 | 0.7790 | 0.001 |  | ABCB1 | 0.7870 | 0.001 |
| NUDT18 | -0.6397 | 0.001 |  | CARD8 | 0.7644 | 0.001 |
| SCAMP2 | 0.6386 | 0.001 |  | TLR5 | 0.7256 | 0.001 |
| LANCL1 | 0.6793 | 0.001 |  | TLR9 | 0.6663 | 0.001 |
| KLHDC8B | -0.7758 | 0.001 |  | IL7R | 0.6896 | 0.001 |
| TSPYL4 | -0.6693 | 0.001 |  | CDC42 | -0.6459 | 0.001 |
| MPI | -0.7136 | 0.001 |  | NXPH1 | 0.6451 | 0.001 |
| KNDC1 | -0.6386 | 0.001 |  | FAAH | -0.5348 | 0.001 |
| FASTKD5 | -0.5032 | 0.001 |  | GPBAR1 | 0.7335 | 0.001 |
| NELFA | -0.6285 | 0.001 |  | ORMDL3 | 0.7132 | 0.001 |
| ERICH2 | -0.6851 | 0.001 |  | TPTE2P3 | -0.5969 | 0.001 |
| PPP6C | -0.799 | 0.001 |  | PIH1D2 | 0.7523 | 0.001 |
| SLC38A3 | -0.6525 | 0.001 |  | TANK | 0.6318 | 0.001 |
| SCAI | -0.5700 | 0.001 |  | P2RY14 | -0.6615 | 0.001 |
| ACSF3 | -0.7821 | 0.001 |  | KIAA1109 | 0.8696 | 0.001 |
| HRH1 | -0.5370 | 0.001 |  | FOXP2 | -0.6328 | 0.001 |
| ANXA11 | -0.6275 | 0.001 |  | DCC | -0.8653 | 0.001 |
| CCR3 | -0.7875 | 0.001 |  | TCF4 | -0.6431 | 0.001 |
| ORM1 | 0.6876 | 0.001 |  | PBX1 | 0.6351 | 0.001 |
| COP1 | -0.8065 | 0.001 |  | SLC4A3 | -0.6278 | 0.001 |
| CIR1 | 0.7579 | 0.001 |  | PCLO | -0.5569 | 0.001 |
| NAA11 | -0.5716 | 0.001 |  | SLC16A9 | -0.5096 | 0.001 |
| BMPR1B | -0.7378 | 0.001 |  | SLC44A2 | -0.6044 | 0.001 |
| TMEM161B | -0.609 | 0.001 |  | TRPV1 | -0.4967 | 0.001 |
| FGF18 | -0.8259 | 0.001 |  | ADRB2 | -0.4770 | 0.001 |
| ASCC3 | -0.7048 | 0.001 |  | URM1 | -0.4161 | 0.001 |
| ZDHHC21 | -0.8363 | 0.001 |  | COMT | -0.4662 | 0.001 |
| TMEM38B | 0.8184 | 0.001 |  | SNRPC | -0.4071 | 0.002 |
| MSI2 | 0.4869 | 0.001 |  | SNCA | -0.3692 | 0.002 |
| CELF4 | -0.5042 | 0.001 |  | AIM2 | -0.5673 | 0.002 |
| UBAP2L | -0.4612 | 0.001 |  | LRP8 | 0.4829 | 0.002 |
| ETV5 | -0.7192 | 0.001 |  | FAM135B | 0.4922 | 0.002 |
| WIPF3 | -0.8000 | 0.001 |  | PDP1 | -0.4006 | 0.002 |
| CNTN5 | -0.6948 | 0.001 |  | ACADL | -0.4191 | 0.002 |
| GNG7 | 0.6426 | 0.001 |  | TSKU | 0.4738 | 0.003 |
| ASIC3 | 0.8364 | 0.001 |  | RIPK2 | -0.3782 | 0.003 |
| OPRM1 | -0.5860 | 0.001 |  | B3GAT1 | -0.3692 | 0.003 |
| SCYL3 | -0.5624 | 0.001 |  | IL6R | 0.4732 | 0.003 |
| DRD4 | -0.8057 | 0.001 |  | EP300 | -0.4812 | 0.003 |
| NMT1 | -0.5336 | 0.001 |  | ZNF501 | 0.4277 | 0.003 |
| GPR27 | -0.5850 | 0.001 |  | CEP170 | 0.3436 | 0.003 |
| CTBP2 | -0.6007 | 0.001 |  | S100A1 | -0.4062 | 0.003 |
| ZNF35 | 0.5268 | 0.001 |  | TNF | 0.4588 | 0.003 |
| LATS1 | 0.4069 | 0.001 |  | SCN9A | -0.3744 | 0.004 |
| SHMT1 | 0.4386 | 0.001 |  | MAU2 | -0.452 | 0.005 |
| PRKAR2A | -0.5085 | 0.001 |  | LPP | 0.3737 | 0.006 |
| ATP2C1 | -0.4588 | 0.001 |  | HK1 | -0.4200 | 0.007 |
| LRRC3B | -0.5222 | 0.001 |  | GINM1 | -0.4780 | 0.007 |
| PDE2A | -0.6145 | 0.001 |  | PRDM1 | 0.4346 | 0.007 |
| FAM120AOS | -0.5536 | 0.001 |  | AMT | -0.4238 | 0.008 |
| CNTNAP2 | -0.4566 | 0.001 |  | C15orf40 | -0.2837 | 0.012 |
| LRP1B | -0.6051 | 0.001 |  | FOLR2 | -0.3099 | 0.012 |
| NFKB1 | 0.3121 | 0.018 |  | BICD2 | -0.3388 | 0.014 |
| PCCB | -0.3827 | 0.020 |  | RPRD2 | 0.3285 | 0.017 |
| IL23A | -0.2951 | 0.024 |  | POU3F3 | 0.3398 | 0.030 |
| UBOX5 | -0.3941 | 0.024 |  | DNMT3B | -0.4458 | 0.040 |
| GALNT2 | 0.2610 | 0.024 |  | RABEP1 | -0.2648 | 0.044 |
| PWWP2A | 0.3479 | 0.025 |  | ADRA2C | -0.2687 | 0.045 |
| RBM6 | -0.4007 | 0.029 |  | IFI16 | 0.2795 | 0.046 |

## Table S11. Cognitive networks showing similar spatial correlation with the somatic network

| **Cognitive terms** | **R values** | ***P* (FDR corrected)** |
| --- | --- | --- |
| action | 0.6175 | 0.0003 |
| adaptation | 0.6145 | 0.0003 |
| addiction | -0.6017 | 0.0003 |
| anticipation | 0.6976 | 0.0003 |
| association | -0.5211 | 0.0003 |
| autobiographical memory | -0.7803 | 0.0003 |
| belief | -0.8195 | 0.0003 |
| categorization | 0.7755 | 0.0003 |
| cognitive | -0.7427 | 0.0003 |
| concept | -0.786 | 0.0003 |
| consciousness | -0.6638 | 0.0003 |
| context | -0.6607 | 0.0003 |
| coordination | 0.8045 | 0.0003 |
| decision | -0.8217 | 0.0003 |
| decision making | -0.8079 | 0.0003 |
| detection | 0.6549 | 0.0003 |
| discrimination | 0.8068 | 0.0003 |
| efficiency | -0.8058 | 0.0003 |
| emotion regulation | -0.6512 | 0.0003 |
| encoding | -0.6934 | 0.0003 |
| episodic memory | -0.8297 | 0.0003 |
| expertise | 0.5132 | 0.0003 |
| facial expression | 0.785 | 0.0003 |
| familiarity | -0.8489 | 0.0003 |
| focus | -0.6474 | 0.0003 |
| gaze | 0.632 | 0.0003 |
| goal | -0.8095 | 0.0003 |
| hyperactivity | -0.7211 | 0.0003 |
| imagery | 0.711 | 0.0003 |
| impulsivity | -0.7732 | 0.0003 |
| induction | 0.8836 | 0.0003 |
| inference | -0.7105 | 0.0003 |
| integration | 0.8526 | 0.0003 |
| intelligence | -0.9113 | 0.0003 |
| intention | -0.8788 | 0.0003 |
| judgment | -0.8975 | 0.0003 |
| knowledge | -0.845 | 0.0003 |
| learning | 0.5076 | 0.0003 |
| listening | 0.7542 | 0.0003 |
| localization | 0.862 | 0.0003 |
| memory | -0.9085 | 0.0003 |
| memory retrieval | -0.8564 | 0.0003 |
| mood | -0.6688 | 0.0003 |
| morphology | 0.5477 | 0.0003 |
| motor control | 0.8173 | 0.0003 |
| movement | 0.8228 | 0.0003 |
| multisensory | 0.8496 | 0.0003 |
| naming | 0.7505 | 0.0003 |
| pain | 0.8501 | 0.0003 |
| perception | 0.8665 | 0.0003 |
| psychosis | -0.6929 | 0.0003 |
| reasoning | -0.8986 | 0.0003 |
| recall | -0.804 | 0.0003 |
| recognition | -0.7765 | 0.0003 |
| reinforcement learning | -0.6735 | 0.0003 |
| retention | -0.6252 | 0.0003 |
| retrieval | -0.874 | 0.0003 |
| rhythm | 0.8968 | 0.0003 |
| risk | -0.7623 | 0.0003 |
| rule | -0.6233 | 0.0003 |
| salience | -0.7056 | 0.0003 |
| search | -0.7182 | 0.0003 |
| selective attention | 0.632 | 0.0003 |
| semantic memory | -0.7602 | 0.0003 |
| skill | 0.5942 | 0.0003 |
| social cognition | -0.7534 | 0.0003 |
| speech perception | 0.8533 | 0.0003 |
| speech production | 0.9118 | 0.0003 |
| strategy | -0.8823 | 0.0003 |
| thought | -0.85 | 0.0003 |
| uncertainty | -0.8479 | 0.0003 |
| updating | -0.6974 | 0.0003 |
| utility | -0.5935 | 0.0003 |
| visual perception | 0.6998 | 0.0003 |
| monitoring | -0.4481 | 0.0006 |
| sentence comprehension | -0.5058 | 0.0006 |
| consolidation | -0.5399 | 0.0010 |
| insight | -0.5206 | 0.0012 |
| planning | 0.5333 | 0.0012 |
| priming | -0.5101 | 0.0015 |
| strength | -0.3055 | 0.0015 |
| object recognition | 0.3831 | 0.0018 |
| distraction | -0.3698 | 0.0021 |
| rehearsal | 0.525 | 0.0032 |
| task difficulty | -0.3275 | 0.0035 |
| spatial attention | 0.3971 | 0.0042 |
| valence | -0.5045 | 0.0042 |
| emotion | -0.5065 | 0.0045 |
| response selection | 0.47 | 0.0047 |
| anxiety | -0.4837 | 0.0076 |
| stress | -0.4498 | 0.0076 |
| balance | 0.4096 | 0.0094 |
| sustained attention | 0.456 | 0.0101 |
| maintenance | -0.3001 | 0.0115 |
| language comprehension | -0.3764 | 0.0186 |
| reading | 0.3639 | 0.0220 |
| interference | 0.326 | 0.0246 |
| fixation | 0.2705 | 0.0299 |
| navigation | -0.3685 | 0.0311 |
| word recognition | 0.3405 | 0.0445 |

## Table S12. Sample and imaging characteristics of brain stimulation improving somatic symptoms

| **Study** | **DOI /** **PMID** | **Disease** | **Number** | **Treatment** | **Target** | **MNI** |
| --- | --- | --- | --- | --- | --- | --- |
| Bühler 2023 | 10.1136/bmjno-2023-000525 | FND | 23 | cTBS/iTBS | rTPJ | (62 -34 30) |
| Jimenez-Torres 2017 | 10.1016/j.cct.2016.12.024 | Chronic pain | 8 | tDCS | DLPFC | F3 of 10-20 EEG system |
| Armbrust 2024 | 10.1177/02692155241274718 | chronic low back pain | 72 | tDCS | M1 | C3/C4 of 10-20 EEG system |
| Mariano 2018 | 10.1093/pm/pny188 | chronic low back pain | 21 | tDCS | dACC | FC1 of 10-20 EEG system |
| Valle 2009 | PMID: 21170277 | Fibromyalgia | 41 | tDCS and rTMS | DLPFC and M1 | C3/F3 of 10-20 EEG system |
| Shi 2024 | 10.1111/cns.70110 | MDD | 70 | tDCS | DLPFC | F3/F4 of 10-20 EEG system |
| Leroy 2019 | 10.1016/j.brs.2019.01.019 | PNES | 1 | tDCS | DLPFC | F3 of 10-20 EEG system |
| Shi 2024 | 10.1111/cns.70110 | MDD | 70 | tDCS | DLPFC | F3/F4 of 10-20 EEG system |

Abbreviations: FND, functional neurological disorder; cTBS, continuous theta-burst stimulation; iTBS, intermittent theta-burst stimulation; rTPJ, right temporoparietal junction; DLPFC, Dorsolateral Prefrontal Cortex; tDCS, transcranial direct current stimulation; M1, primary motor cortex; dACC, dorsal anterior cingulate cortex; OFC, orbitofrontal cortex; PNES, psychogenic Non-Epileptic Seizures; MDD, major depressive disorder.

## Table S13. Demographic and clinical characteristics of the participants in normative model analysis

| **Group** | **Number** | **Age**  **(years)** | **Gender (M/F)** | **Duration (months)** | **PHQ-15** |
| --- | --- | --- | --- | --- | --- |
| Healthy individuals | 1342 | 30.61 (0.45) | 629/713 | - | - |
| somatic patients | 399 | 30.77 (0.64) | 128/271 | 50.78 (3.34) | 11.30 (0.25) |
| GAD | 101 | 34.38 (1.16) | 36/65 | 47.33(5.88) | 11.06 (0.41) |
| Depression | 273 | 29.57 (0.79) | 77/196 | 47.02 (3.91) | 11.73 (0.31) |
| Other disease | 25 | 30.04 (2.27) | 15/10 | 105.16 (18.47) | 7.60 (0.91) |

Age, duration and PHQ-15 were expressed as mean (standard deviation). Other disease included bipolar disorder and schizophrenia. Abbreviations: F, female; M, male; PHQ-15, health questionnaire-15; GAD, generalized anxiety disorder.

# Supplementary Figures

**Fig. S1. Flow diagram of the literature search for identifying lesions causing somatic symptoms.**

341 articles matched initial search criteria. Of these, 221 articles were excluded before screening because of duplication and other reasons. 1866 articles were excluded after screening the title and abstract. The left 45 articles for full-text review to assess for eligibility. Of these, 13 had suitable imaging for lesion network mapping, and a total of 21 lesions were included in the current study.

# Fig S2. Brain lesions causing somatic symptoms.

**Fig. S3. Flow diagram of the literature search for identifying brain structural and functional abnormalities of somatic symptoms.**

5948 articles matched initial search criteria. Of these, 2409 articles were excluded before screening because of duplication and other reasons. 3347 articles were excluded after screening the title and abstract. The left 192 articles for full-text review to assess for eligibility. Of these, 68 were suitable for analysis including 26 GMV studies, 12 ALFF studies, 16 ReHo studies and 14 task-fMRI studies. GMV, gray matter volume; ALFF, low-frequency fluctuation amplitudes; ReHo, regional homogeneity.

**Fig. S4. Lesion network mapping in disease connectome (n = 190).**

**Fig. S5. Lesion network mapping using a 4mm lesion extension.**

**Fig. S6. Lesion network mapping using 85% overlapping regions of the sensitivity map and both specificity maps.**

**Fig. S7. Comparison of 4 somatic lesion networks.**

(a) The initial somatic lesion network was mapped using the normative connectome database (n = 652). (b) The somatic lesion network derived from disease connectome data (n = 190) demonstrated a strong correlation with the original network (*r* = 0.95, *p* < 0.001). (c) The somatic lesion network generated using 4mm lesion tracing also exhibited a high correlation with the original network (*r* = 0.95, *p* < 0.001). (d) The somatic lesion network created from an 85% sensitivity map maintained a robust correlation with the original network (*r* = 0.95, *p* < 0.001).

## Fig S8 Validation of multimodal imaging findings in somatic symptoms by symptom.

ANOVA analysis revealed that FC of somatic-related ROIs differed significantly from two control ROIs with somatic network (*F* = 2.618, *p* = 0.025). Post hoc analysis revealed that pain related ROIs had significantly stronger FC with somatic network compared to random ROIs (*p* = 0.034); gastrointestinal related ROIs had significantly stronger FC with somatic network compared to both the non-somatic ROIs (*p* = 0.035) and random ROIs (*p* = 0.012); anxiety and depression with somatic symptom related ROIs had significantly stronger FC with somatic network compared to both the non-somatic ROIs (*p* = 0.045) and random ROIs (*p* = 0.018). The general somatic ROIs showed on significant FC with non-somatic ROIs (*p* = 0.388) and random ROIs (*p* = 0.185). GI, gastrointestinal symptoms; MDD, major depressive disorder. * *p* < 0.05.

## Fig S9 Validation of multimodal imaging findings in somatic symptoms by indicator.

ANOVA analysis revealed that FC of somatic-related ROIs differed significantly from two control ROIs with somatic network (*F* = 3.611, *p* = 0.014). Post hoc analysis revealed that ROIs of function indicator had significantly stronger FC with somatic network compared to both the non-somatic ROIs (*p* = 0.007) and random ROIs (*p* = 0.003). ** *p* < 0.001.

**Fig. S10. Model fit evaluation**.

The distribution and brain map of EXPV, MSLL and SMSE. Abbreviations: EXPV, explained variance; MSLL, mean squared log-loss; SMSE, standardized mean squared error.

## Fig. S11. Individual atrophy loci.

(a) Examples of the location of atrophy loci in patients. (b) The regions with overlapping degrees higher than 5% across 247 somatic patients.

**Fig. S12. Partial correlation analysis between atrophy volume in somatic network and PHQ-15 scores (controlling for age, sex and total atrophy volume as covariates).**

**Fig. S13. Correlation analysis between atrophy volume in somatic network and HAMA, HAMD scores.**
