# Supplementary material for: A lesion-derived brain network of somatic symptoms for transdiagnostic individual application
Source: BMC Med. 2026 Jul 2;24:510. doi: 10.1186/s12916-026-05044-y (PMC13599187; doi:10.1186/s12916-026-05044-y)
Supplement: Supplementary file 3 [file 12916_2026_5044_MOESM3_ESM.docx]

**Table D.1. Experimental Design Reporting**

| **Aspect** | **Notes** | **Mandatory** | **Location** |
| --- | --- | --- | --- |
| **Number of subjects** | *Elaborate each by group if have more than one group.* |  |  |
| Subjects approached |  | N | **LNM:**  Methods, page 8, paragraph 3;  Additional file 1: Methods S2, page 4, paragraph3  **Normative modeling:**  Methods, page 17, paragraph 1;  Additional file 1: Method S8, page 8, paragraph 2 |
| Subjects consented |  | N | Methods, page 7, paragraph 1 |
| Subjects refused to participate | Provide reasons. | N | N/A |
| Subjects excluded | Subjects excluded after consenting but before data acquisition; provide reasons. | N | N/A |
| Subjects participated and analyzed | Provide the number of subjects scanned, number excluded after acquisition, and the number included in the data analysis. If they differ, note the number of subjects in each particular analysis. | Y | **LNM:**  Methods, page 8, paragraph 3;  Additional file 1: Methods S2, page 4, paragraph3  **Normative modeling:**  Methods, page 17, paragraph 1;  Additional file 1: Method S8, page 8, paragraph 2 |
| **Inclusion criteria and descriptive statistics** | *Elaborate each by group if have more than one group.* |  |  |
| Age | Mean, standard deviation and range. | Y | **LNM:**  Methods, page 8, paragraph 3; Additional file 1: Methods S2, page 4, paragraph3; Table S1  **Normative modeling:**  Methods, page 17, paragraph 1;  Additional file 1: Methods S8, page 8, paragraph 2; Table S13 |
| Sex | Absolute counts or relative frequencies. | Y | **LNM:**  Methods, page 8, paragraph 3; Additional file 1: Methods S2, page 4, paragraph3; Table S1  **Normative modeling:**  Methods, page 17, paragraph 1;  Additional file 1: Methods S8, page 8, paragraph 2; Table S13 |
| Race & ethnicity | Per guidelines of NIH or other relevant agency. | N | N/A |
| Education, SES | Education is essential for studies comparing patient and control groups; complete SES reporting less important for single-group studies, but still useful.  Specify measurement instrument used; may be parental SES and education if study has minors. | Y | N/A (single-group studies and Large-scale human MRI dataset) |
| IQ | Specify measurement instrument used. | N | N/A |
| Handedness | Absolute or relative frequencies; basis of handedness-attribution (self-report, EHI, other tests). (Important for fMRI, may be less important for structural studies.) | Y | **LNM:**  Additional file 1: Methods S2, page 4, paragraph3  **Normative modeling:**  Additional file 1: Methods S8, page 8, paragraph 2 |
| Exclusion criteria | Describe any screening criteria, including those applied to “normal” sample such as MRI exclusion criteria. | Y | **LNM:**  Additional file 1: Methods S2, page 4, paragraph 3; page 5, paragraph 2  **Normative modeling:**  Additional file 1: Methods S8, page 8, paragraph 2 and paragraph 3 |
| Clinical criteria | Detail the area of recruitment (in- vs. outpatient setting, community hospital vs. tertiary referral center etc.) as well as whether patients were currently in treatment. | Y | **LNM:**  Methods, page 8, paragraph 3; Additional file 1: Methods S2, page 5, paragraph 2.  **Normative modeling:**  Methods, page 17, paragraph 1; Additional file 1: Methods S8, page 8, paragraph 3. |
| Clinical instruments | Describe the instruments used to obtain the diagnosis and provide tests of intra- or inter-rater reliability. Clarify whether a “clinical diagnosis” or “inventory diagnosis” was used (if applicable). State the diagnostic system (ICD, DSM etc.) that was used. | Y | **LNM:**  Additional file 1: Methods S2, page 5, paragraph 2  **Normative modeling:**  Additional file 1: Methods S8, page 8, paragraph 3 |
| Matching strategy | If applicable. | Y | N/A (single-group studies and Large-scale human MRI data) |
| Population & recruitment strategy | Population from which subjects were drawn, and how and where recruitment took place, e.g., schools, clinics, etc. If possible, note if subjects are research-naive or have participated in other studies before. | Y | **LNM:**  Methods, page 8, paragraph 3;  **Normative modeling:**  Methods, page 17, paragraph 1. |
| Subject scanning order | With multiple groups, information on ordering and or balance over time; especially report relative to scanner changes/upgrades. (Ideally, use randomized or interleaved order to avoid bias due to scanner changes/upgrades.) | Y | **LNM:**  Additional file 1: Methods S2, page 5, paragraph 3  **Normative modeling:**  Additional file 1: Methods S8, page 8, paragraph 4 |
| Neurocognitive measures | All measures collected on subjects should be described and reported. | Y | **LNM:**  Additional file 1: Methods S2, page 4, paragraph3; Table S1  **Normative modeling:**  Additional file 1: Methods S8, page 8, paragraph 2; Table S13 |
| **Ethical considerations** |  |  |  |
| Ethical approval | Describe approval given, including the particular institutional review board, medical ethics committee or equivalent that granted the approval. When data is shared, describe the ethics/institutional approvals required from either the author (source) or recipient. | Y | Methods, page 7, paragraph 1 |
| Informed consent | Record whether subjects provided informed consent or, if applicable, informed assent. | Y | Methods, page 7, paragraph 1 |
| **Design specifications** |  |  |  |
| Design type | Task or resting state. Event_related or block design. (See body text for usage of ‘block design’ terminology.) | Y | **LNM:**  Methods, page 8, paragraph 3;  **Normative modeling:**  Methods, page 17, paragraph 1. |
| Condition & stimuli | Clearly describe each condition and the stimuli used. Be sure to completely describe baseline (e.g. blank white/black screen, presence of fixation cross, or any other text), especially for resting-state studies. When possible provide images or screen snapshots of the stimuli. | Y | **LNM:**  Additional file 1: Methods S2, page 5, paragraph 3  **Normative modeling:**  Additional file 1: Methods S8, page 8, paragraph 4 |
| Number of blocks, trials or experimental units | Specify per session, and if differing by subject, summary statistics (mean, range and/or standard deviation) of such counts. | Y | N/A (resting-state fMRI and structural MRI) |
| Timing and duration | Length of each trial or block (both, if trials are blocked), and interval between trials. Provide the timing structure of the events in the task, whether a random/jittered pattern or a regular arrangement; any jittering of block onsets. | Y | N/A (resting-state fMRI and structural MRI) |
| Length of the experiment | Describe the total length of the scanning session, as well as the duration of each run. (Important to assess subject fatigue.) | Y | **LNM:**  Additional file 1: Methods S2, page 5, paragraph 3  **Normative modeling:**  Additional file 1: Methods S8, page 8, paragraph 4 |
| Design optimization | Whether design was optimized for efficiency, and how. | Y | N/A |
| Presentation software | Name software, version and operating system on which the stimulus presentation was run. When possible, provide code used to drive experiment. | Y | **LNM:**  Additional file 1: Methods S2, page 5, paragraph 4  **Normative modeling:**  Additional file 1: Methods S8, page 8, paragraph 5 |
| **Task specification** |  |  |  |
| Condition | Enumerate the conditions and fully describe and reference each. Consider using a shorthand name, e.g. AUDSTIM, VISSTIM, to refer to each condition, to clarify the distinction between a specific modeled effect and a psychological construct.  Naming should reflect the distinction between instruction periods and actual stimuli, and between single parameters and contrasts of parameters. | Y | N/A (resting-state fMRI and structural MRI) |
| Instructions | Specify the instructions given to subjects for each condition (ideally the exact text in supplement or appendix). For resting-state, be sure to indicate eyes-closed, Eyes-open, any fixation. Describe if the subjects received any rewards during the task, and state if there was a familiarization / training inside or outside the scanner. | Y | N/A (resting-state fMRI and structural MRI) |
| Stimuli | Specifics of stimuli used in each run. For example, the unique number of stimuli used, and whether/how stimuli were repeated over trials or conditions. | Y | N/A (resting-state fMRI and structural MRI) |
| Randomization | Describe block or event ordering as deterministic, or report manner of randomization, in terms of order and timing. If pseudo-randomized, i.e. under constraints, describe how and the criteria used to constrain the orders/timings. | Y | N/A (resting-state fMRI and structural MRI) |
| Stimulus presentation & response collection. | Specify the presentation hardware (e.g. back projection, in-room display, goggles, etc.), and the response systems (e.g. button boxes, eye tracking, physiology).  Note how equipment was synched to the scanner (e.g. scanner TTL, or manual sync.) | Y | N/A (resting-state fMRI and structural MRI) |
| Run order | Order in which tasks runs are conducted in the scanner. | Y | N/A (resting-state fMRI and structural MRI) |
| **Power analysis** |  |  |  |
| Outcome | Specify the type of outcome used as the basis of power computations, e.g. signal in a pre-specified ROI, or whole image voxelwise (or cluster-wise, peak-wise, etc.). | Y | **LNM:**  Methods, page 9, paragraph 2;  **Normative modeling:**  Methods, page 17, paragraph 2. |
| Power parameters | Specify  ● Effect size (or effect magnitude and standard deviation separately).  ● Source of predicted effect size (previous literature with citation; pilot data with description, etc.).  ● Significance level (e.g. uncorrected alpha 0.05 for an ROI, or FEW-corrected significance.  ● Target power (typically 80%).  ● Any other parameters set (e.g., for spatial methods a brain volume and smoothness may be needed to be specified). | Y | **LNM:**  Methods, page 9, paragraph 2;  **Normative modeling:**  Methods, page 17, paragraph 2. |
| **Behavioral performance** |  |  |  |
| Variables recorded | State number of type of variables recorded (e.g. correct button press, response time). | Y | N/A (No behavioral studies were conducted) |
| Summary statistics | Summaries of behavior sufficient to establish that subjects were performing the task as expected. For example, correct response rates and/or response times, summarized over subjects (e.g. mean, range and/or standard deviation). | Y | N/A (No behavioral studies were conducted) |

**Table D.2. Acquisition Reporting**

| **Aspect** | **Notes** | **Mandatory** | **Location** |
| --- | --- | --- | --- |
| **Subject preparation** |  |  |  |
| Mock scanning | Use of an MRI simulator to acclimate subjects to scanner environment. Report type of mock scanner and protocol (i.e. duration, types of simulated scans, experiments). | N | N/A |
| Special accommodations | For example, for pediatric scanning, presence of parent/guardian in the room. | Y | N/A (no special accommodations and carefully checked the MRI contraindications) |
| Experimenter personnel | Whether a single or multiple experimenters interacted with the subjects. | N | **LNM:**  Additional file 1: Methods S2, page 5, paragraph 4  **Normative modeling:**  Additional file 1: Methods S8, page 8, paragraph 5 |
| **MRI system description** |  |  |  |
| Scanner | Provide make, model & field strength in tesla (T). | Y | **LNM:**  Additional file 1: Methods S2, page 5, paragraph 3  **Normative modeling:**  Additional file 1: Methods S8, page 8, paragraph 4 |
| Coil | Receive coil (e.g. “a 12-channel phased array coil”, but more details for a custom coil) and (if nonstandard) transmit coil. Additional information on the gradient system, e.g. gradient strength (if non-standard for the make and model, or switchable). | Y | **LNM:**  Additional file 1: Methods S2, page 5, paragraph 3  **Normative modeling:**  Additional file 1: Methods S8, page 8, paragraph 4 |
| Significant hardware modifications | For example, special gradient inserts/sets. | N | N/A |
| Software version | Highly recommended when sharing vendor-specific protocols or exam cards, as version may be needed to correctly interpret that information. | N | **LNM:**  Additional file 1: Methods S3, page 5, paragraph 4  **Normative modeling:**  Additional file 1: Methods S8, page 8, paragraph 5 |
| **MRI acquisition** |  |  |  |
| Pulse sequence type | For example, gradient echo, spin echo, etc. | Y | **LNM:**  Additional file 1: Methods S3, page 5, paragraph 3  **Normative modeling:**  Additional file 1: Methods S8, page 8, paragraph 4 |
| Imaging type | For example, echo planar imaging (EPI), spiral, 3D.  Number of shots (if multi-shot); partial Fourier scheme & reconstruction method (if used) | Y | **LNM:**  Additional file 1: Methods S3, page 5, paragraph 3  **Normative modeling:**  Additional file 1: Methods S8, page 8, paragraph 4 |
| Essential sequence & imaging parameters. | For all acquisitions:  ● Echo time (TE).  ● Repetition time (TR).  ○ For multi-shot acquisitions, additionally the time per volume.  ● Flip angle (FA).  ● Acquisition time (duration of acquisition).  Functional MRI:  ● Number of volumes.  ● Sparse sampling delay (delay in TR) if used.  Inversion recovery sequences:  ● Inversion time (TI).  B0 field maps:  ● Echo time difference (dTE).  Diffusion MRI:  ● Number of directions.  ○ Direction optimization, if used and type.  ● b-values.  ● Number of b=0 images.  ● Number of averages (if any).  ● Single shell, multi-shell (specify equal or unequal spacing).  ● Single_ or dual-spin-echo, gradient mode (serial or parallel).  ● If cardiac gating used.  Imaging parameters:  ● Field of view.  ● In-plane matrix size, slice thickness and interslice gap, for 2D acquisitions.  ● Slice orientation:  ○ Axial, sagittal, coronal or oblique.  ○ Angulation: If acquistion not aligned with scanner axes, specify angulation to AC-PC line (see Slice position procedure).  ● 3D matrix size, for 3D acquisitions. | Y | **LNM:**  Additional file 1: Methods S3, page 5, paragraph 3  **Normative modeling:**  Additional file 1: Methods S8, page 8, paragraph 4 |
| Phase encoding | Specify phase encoding direction (e.g. as A/P, L/R, or S/I). For 3D, specify “partition encode” (aka slice) direction.  Phase encoding reversal: Mention if used (aka “blip-up/blip-down”). | Y | **LNM:**  Additional file 1: Methods S3, page 5, paragraph 3  **Normative modeling:**  Additional file 1: Methods S8, page 8, paragraph 4 |
| Parallel imaging method & parameters | Report:  ● Method, e.g. SENSE, GRAPPA or other parallel imaging method, and acceleration factor.  ● Matrix coil mode, and coil combining method (if non-standard). | Y | **LNM:**  Additional file 1: Methods S3, page 5, paragraph 3  **Normative modeling:**  Additional file 1: Methods S8, page 8, paragraph 4 |
| Multiband parameters | Multiband factor and field-of-view shift (only if applicable). | Y | N/A |
| Readout parameters | Receiver bandwidth, readout duration, echo spacing. | N | **LNM:**  Additional file 1: Methods S3, page 5, paragraph 3  **Normative modeling:**  Additional file 1: Methods S8, page 8, paragraph 4 |
| Fat suppression | For anatomical scans, whether it was used or not. | Y | N/A |
| Shimming | Any specialized shimming procedures. | Y | **LNM:**  Additional file 1: Methods S3, page 5, paragraph 3  **Normative modeling:**  Additional file 1: Methods S8, page 8, paragraph 4 |
| Slice order & timing | For fMRI acquisitions, interleaved vs. sequential ordering and direction (ascending/descending), location of 1st slice; any specialized slice timing. | Y | Additional file 1: Methods S3, page 5, paragraph 3 |
| Slice position procedure | For example, landmark guided [vs. auto-alignment](vs.auto_alignment). | N | N/A |
| Brain coverage | Report whether coverage was whole-brain, and whether cerebellum and  brainstem were included. If not whole-brain, note the nature of the partial area of coverage. If axial and co-planar with AC-PC line, the volume coverage in terms of Z in mm. | Y | **LNM:**  Additional file 1: Methods S3, page 5, paragraph 3  **Normative modeling:**  Additional file 1: Methods S8, page 8, paragraph 4 |
| Scanner-side preprocessing | Including:  ● Reconstruction matrix size differing from acquisition matrix size.  ● Prospective-motion correction (including details of any optical tracking, and how motion parameters are used).  ● Signal inhomogeneity correction.  ● Distortion-correction. | Y | **LNM:**  Additional file 1: Methods S3, page 5, paragraph 3  **Normative modeling:**  Additional file 1: Methods S8, page 8, paragraph 4 |
| Scan duration | ln seconds | N | **LNM:**  Additional file 1: Methods S3, page 5, paragraph 3  **Normative modeling:**  Additional file 1: Methods S8, page 8, paragraph 4 |
| Other non-standard procedures | Including:  ● Turning off the cold head(s) (e.g. during EEG/fMRl or spectroscopy measurements).  ● Reduce sound pressure by limiting the gradient slew rate. | N | N/A |
| T1 stabilization | Number of initial “dummy” scans acquired and then discarded by the scanner. | Y | Additional file 1: Methods S8, page 9, paragraph 1 |
| Diffusion MRI gradient table | Also referred to as the b-matrix (but not to be confused with the 3×3 matrix that describes diffusion weighting for a single diffusion weighted measurement). | N | N/A |
| Perfusion: Arterial Spin Labelling MRI | ● ASL Labelling method (e.g. continuous ASL (CASL), pseudo-continuous ASL (PCASL), Pulsed ALS (PASL), velocity selective ASL (VSASL)).  ● Use of background suppression pulses and their timing.  ● For either PCASL or CASL report:  ○ Label Duration.  ○ Post-labeling delay (PLD).  ○ Location of the labeling plane.  ● For PCASL also report:  ○ Average labeling gradient.  ○ Slice-selective labeling gradient.  ○ Flip angle of B1 pulses.  ○ Assessment of inversion efficiency; QC used to ensure off-resonance artifacts not problematic, signal obtained over whole brain.  ● For CASL also report:  ○ Use of a separate labeling coil.  ○ Control scan/pulse used.  ○ B1 amplitude.  ● For PASL report  ○ TI.  ○ Labeling slab thickness.  ○ Use of QUIPSS pulses and their timing.  ● For VSASL  ○ TI.  ○ Choice of velocity selection cutoff (“VENC”). | Y | N/A (No perfusion imaging analysis was conducted) |
| Perfusion: Dynamic  Susceptibility Contrast MRI | Specify:  ● Number of baseline volumes.  ● Type, name and manufacturer of intravenous bolus (e.g. gadobutrol, Gadavist, Bayer).  ● Bolus amount and concentration (e.g. 0.1 ml/kg and 0.1 mmol/kg).  ● Injection rate (e.g. 5 ml/s).  ● Post-injection of saline (e.g. 20 ml).  ● Injection method (e.g. power injector). | Y | N/A (No perfusion imaging analysis was conducted) |
| **Preliminary quality control** |  |  |  |
| Motion monitoring | For functional or diffusion acquisitions, any visual or quantitative checks for severe motion; likewise, for structural images, checks on motion or general image quality. | Y | **LNM:**  Additional file 1: Methods S3, page 5, paragraph 3  **Normative modeling:**  Additional file 1: Methods S8, page 9, paragraph 1 |
| Incidental findings | Protocol for review of any incidental findings, and how they are handled in particular with respect to possible exclusion of a subject’s data. | N | Additional file 1: Methods S2, page 5, paragraph 4 |

**Table D.3. Preprocessing Reporting**

| **Aspect** | **Notes** | **Mandatory** | **Location** | |  |
| --- | --- | --- | --- | --- | --- |
| Software | For each software used, be sure to include version and revision number. | Y | Methods, page 15, paragraph 2 and paragraph 3; page 15, paragraph 2  Additional file 1: Methods S3, page 5, paragraph 5; page 7, paragraph 3; page 9, paragraph 2; page 10, paragraph 1 | |  |
| Software citation | Include URL and Research Resource Identifier for each software used. | N | Methods, page 15, paragraph 2 and paragraph 3; page 15, paragraph 2  Additional file 1: Methods S3, page 5, paragraph 5; page 7, paragraph 3; page 9, paragraph 2; page 10, paragraph 1 | |  |
| T1 stabilization | Number of initial “dummy” scans discarded as part of preprocessing (if not already performed by scanner). | Y | Additional file 1: Methods S8, page 9, paragraph 2 | |  |
| Brain extraction | If performed, report:  ● Name of software/method (e.g., BET, recon-all in FreeSurfer, etc).  ● Parameter choices (e.g. BET’s fractional intensity threshold).  ● Any manual editing applied to the brain masks. | Y | Additional file 1: Methods S8, page 9, paragraph 2 | |  |
| Segmentation | For structural images, method used to extract gray, white, CSF and other tissue classes. | Y | Additional file 1: Methods S8, page 9, paragraph 2 | |  |
| Slice time correction | If performed, report:  ● Name of software/method.  ● Whether performed after or before motion correction.  ● Reference slice.  ● Interpolation type and order (e.g., 3rd order spline or sinc). | Y | Additional file 1: Methods S8, page 9, paragraph 2 | |  |
| Motion correction | Report:  ● Name of software/method.  ● Use of non-rigid registration, and if so the type of transformation.  ● Use of motion susceptibility correction (fieldmap-based unwarping), as well as the particular software/method.  ● Reference scan (e.g. 1st scan or middle scan).  ● Image similarity metric (e.g. normalized correlation, mutual information, etc).  ● Interpolation type (e.g., spline, sinc), and whether image transformations are combined to allow a single interpolation.  ● Use of any slice-to-volume registration methods, or integrated with slice time correction. | Y | Additional file 1: Methods S8, page 9, paragraph 2 | |  |
| Gradient distortion correction | (If not already described as part of motion susceptibility correction.) | Y | Additional file 1: Methods S3, page 5, paragraph 5 |  |  |
| Diffusion MRI eddy current correction | Report:  ● Name of software/method, and if integrated with motion correction  ● Image similarity / cost function.  ● Type of transformation (e.g. rigid body, affine) and whether constrained only along the phase encode direction.  ● Note if gradient table (b-matrix) is then re-oriented.  ● Volumetric change applied for eddy current along the phase-encode axis (by the Jacobian determinant). | Y | N/A (No Diffusion MRI analysis was conducted) |  |  |
| Diffusion estimation | For all methods, report  ● Model, parameterisation and number of free parameters.  ● Estimation method.  ● Outlier handling approach.  ● Some evidence of fit quality; e.g sample of slices of diffusion weighted data, or residual maps.  Items to note for particular approaches:  ● Tensor or Kurtosis.  ○ Any parameter constraints, like cylindrical symmetry.  ● Multi-compartmental models.  ○ Compartments of the model.  ● Orientation distribution function.  ○ Parametric (model) or nonparametric (basis function) model.  ○ Whether orientation distribution function or fibre orientation density is reported.  ○ For spherical deconvolution, note how the canonical fibre response function is derived (e.g. from the data themselves, or simulated  data). | Y | N/A (No Diffusion MRI analysis was conducted) |  |  |
| Diffusion processing | Report:  ● Summary measures computed (FA, MD, AD, RD, MK, AK, RK, etc.).  ● Whether a track based or voxel-wise method is used.  ● Threshold used to define analysis voxels.  ● Use of population reference track atlas vs. custom atlas (specify set of subjects used to create atlas).  ● Standard deviation map (across subjects). | N | N/A (No Diffusion MRI analysis was conducted) | | |
| Diffusion tractography | Report:  ● Name of software/method.  ● Step size, turning angle and stopping criteria.  ● For ROI based analysis, definition of ROIs (e.g. specify the images used to draw ROIs; manual, semi-automatic or automatic definition of ROIs).  ● For tracking, note step-size, turning angle, any anatomical constraints imposed, and stopping criteria.  ● If a measure of path probability / “connectivity” is extracted, clearly define this measure. | Y | N/A (No Diffusion MRI analysis was conducted) | | |
| Perfusion: Arterial Spin Labeling | Report modelling/post-processing scheme:  ● For subtraction, specify whether simple subtraction, running, sinc-subtraction, etc.  ● For quantitative model, specify model used, number of free parameters. | Y | N/A (No perfusion imaging analysis was conducted) | | |
| Perfusion: Dynamic  Susceptibility Contrast MRI | ● How concentration time curves are calculated, e.g. use of T1 corrections (if short TR) or corrections for leakage.  ● Selection of arterial input function (e.g. manual or automatic with reference to method).  ● Deconvolution method (kinetic model) to estimate residue function (e.g. SVD or parametric model).  ● Details of parameter calculations (e.g. CBF, CBV, MTT, TTP, T max). | Y | N/A (No perfusion imaging analysis was conducted) | | |
| Function-structure (intra-subject) coregistration | Report:  ● Name of software/method.  ● Type of transformation (rigid, nonlinear); if nonlinear, type of transformation  ● Cost function (e.g., correlation ratio, mutual information, boundary-based registration, etc.).  ● lnterpolation method (e.g., spline, linear).  Note this step might not be necessary if direct T2* to a functional template registration is used. | Y | Additional file 1: Methods S3, page 5, paragraph 5; page 7, paragraph 3; page 9, paragraph 2; page 10, paragraph 1 | | |
| Distortion correction | Use of any distortion correction due to field or gradient nonlinearity. | Y | Additional file 1: Methods S3, page 5, paragraph 5 | | |
| Intersubject registration | Report:  ● Name of software/method (e.g., FSL flirt followed by fnirt, FreeSurfer, Caret, Workbench, etc)  ● Whether volume and/or surface based registration is used (if not already clearly implied).  ● Image types registered (e.g. T2* or T 1).  ● Any preprocessing to images; e.g. for T1, bias field correction, or  segmentation of gray matter; for T2*, single image (specify image) or mean image.  ● Template space (e.g., MNI, Talairach, fsaverage, FS_LR), modality (e.g., T1, T2*), resolution (e.g., 2mm, fsaverage5, 32k_FS_LR), and the specific name of template image used; note the domain of the template if not whole brain, i.e. cortical surface only, cerebellum only, CIFTI ‘grayordinates’. (cortical surface vertices + subcortical gray matter voxels), etc.  ● Additional template transformation for reporting; e.g., if using a template in MNI space, but reporting coordinates in Talairach, clearly note and report method used (e.g., Brett’s mni2tal, Lancaster’s icbm_spm2tal).  ● Choice of warp (rigid, nonlinear); if nonlinear, transformation type (e.g., B-splines, stationary velocity field, momentum, non-parametric  displacement field); if a parametric transformation is used, report resolution, e.g., 10x10x10 spline control points.  ● Use of regularization, and the parameter(s) used to set degree of regularization.  ● Interpolation type (e.g., spline, linear); if projection from volume to surface space, how were voxels sampled from the volume (e.g., trilinear; nearest neighbor; ribbon-constrained specifying inner and outer surface used).  ● Cost function (e.g., correlation ratio, mutual information, SSD).  ● Use of cost-function masking. | Y | Additional file 1: Methods S3, page 5, paragraph 5 | | |
| lntensity correction | Bias field corrections for structural MRI, but also correction of odd versus even slice intensity differences attributable to interleaved EPI acquisition without gaps. | Y | Additional file 1: Methods S3, page 5, paragraph 5  Additional file 1: Methods S8, page 9, paragraph 2 |  |  |
| lntensity normalization | Scan_by_scan or run-wide scaling of image intensities before statistical modelling. E.g. SPM scales each run such that the mean image will have mean intracerebral intensity of 100; FSL scales each run such that the mean image will have an intracerebral mode of 10,000. | N | Additional file 1: Methods S3, page 5, paragraph 5 |  |  |
| Artifact and structured noise removal | Use of physiological noise correction method.  Report:  ● Name of software/method used (e.g. CompCor, lCA-FlX, lCA-AROMA, etc.).  ● If using a nuisance regression method, specify regressors used; for each type, include key details, as follows:  ○ Motion parameters.  ■ Expansion basis and order (e.g. 1st temporal derivatives; Volterra kernel expansion)  ○ Tissue signals.  ■ Tissue type (e.g., whole brain, gray matter, white matter, ventricles).  ■ Tissue definition (e.g., a priori seed, automatic segmentation, spatial regression).  ■ Signal definition (e.g., mean of voxels, first singular vector, etc.).  ○ Physiological signals  ■ e.g., heart rate variability, respiration.  ■ Modeling choices (e.g. RETROICOR, cardiac and/or  respiratory response functions) and number of computed  regressors. | Y | Additional file 1: Methods S3, page 5, paragraph 5 |  |  |
| Volume censoring | Remediation of problem scans, also known as “scrubbing” or “de-spiking”.  Report:  ● Name of software/method.  ● Criteria (e.g., frame-by-frame displacement threshold, percentage BOLD change).  ● Use of censoring or interpolation; if interpolation, method used (e.g., spline, spectral estimation). | Y | Additional file 1: Methods S3, page 5, paragraph 5 | |  |
| Resting state fMRI feature | Creation of summary measure like ALFF, fALFF, ReHo.  For ALFF, fALFF report:  ● Lower and upper band pass frequencies.  For ReHo, report:  ● Neighborhood size used to compute local similarity measures (e.g. 6, 18 or 26).  ● Similarity measure (e.g. Kendall’s coefficient of concordance). | Y | Methods, page 9, paragraph 2 | |  |
| Spatial smoothing | If this preprocessing step is performed, report:  ● Name of software/method.  ● Size and type of smoothing kernel.  ● Filtering approach, e.g., fixed kernel or iterative smoothing until fixed FWHM.  ● Space in which smoothing is performed (i.e. native volume, native surface, MNI volume, template surface). | Y | Additional file 1: Methods S3, page 5, paragraph 5 | |  |
| Quality control reports | Summaries of subject motion (e.g. mean framewise displacement), image variance (e.g. DVARS), and note of any other irregularities found (e.g. motion or poor SNR not sufficiently severe to warrant exclusion). Should be included with any publically shared data. | N | Additional file 1: Methods S3, page 5, paragraph 5 | |  |

**Table D.4. Statistical Modeling ＆ Inference**

| **Aspect** | **Notes/Ontology** | **Mandatory** | **Location** | | | |
| --- | --- | --- | --- | --- | --- | --- |
| **Mass univariate analyses** |  |  |  | | | |
| Dependent variable: Data  submitted to statistical modeling | Report the number of time points, number of subjects; specify exclusions of time points / subjects, if not already specified in experimental design. | Y | Methods, page 16-18  Additional file 1: Method S8, Page 8-10 | | | |
| Dependent variable: Spatial region modeled | If not “Full brain”, give a specification of an anatomically or functionally defined mask. | Y | Methods, page 16-18  Additional file 1: Method S8, Page 8-10 | | | |
| Independent variables | For first level fMRI, specify:  ● Event_related design predictors.  ○ Modeled duration, if other than zero.  ○ Parametric modulation.  ● Block Design predictors.  ○ Note whether baseline was explicitly modeled.  ● HRF basis, typically one of:  ○ Canonical only.  ○ Canonical plus temporal derivative.  ○ Canonical plus temporal and dispersion derivative.  ○ Smooth basis (e.g. SPM “informed” or Fourier basis; FSL’s FLOBS).  ○ Finite Impulse Response model.  ● Drift regressors (e.g. DCT basis in SPM, with specified cut_off).  ● Movement regressors; specify if squares and/or temporal derivative used.  ● Any other nuisance regressors, and whether they were entered as  interactions (e.g. with a task effect in 1st level fMRI, or with group effect).  ● Any orthogonalization of regressors, and set of other regressors used to orthogonalize against.  For second level fMRI or general group model, specify:  ● Group effects (patients vs. controls).  ● Clearly state whether or not covariates are split by group (i.e. fit as a group-by-covariate interaction).  ● Other between subject effects (age, sex; for VBM, total GM or ICV). For group model with repeated measures, specify:  ● How condition effects are modeled ([e.g. as](e.g.as) factors, or as linear trends).  ● Whether subject effects are modeled ([i.e. as](i.e.as) regressors, as opposed to with a covariance structure). | Y | Methods, page 16-18  Additional file 1: Method S8, Page 8-10 | | | |
| Model type | Some suggested terms include:  ● “Mass Univariate”.  ● “Multivariate” (e.g. ICA on whole brain data).  ● “Mass Multivariate” (e.g. MANOVA on diffusion or morphometry tensor data).  ● “Local Multivariate” (e.g. “searchlight”).  ● “Multivariate, intra-subject predictive” (e.g. classify individual trials in event-related fMRI).  ● “Multivariate inter-subject predictive” (e.g. classify subjects as patient vs. control).  ● “Representational Similarity Analysis”. | Y | Methods, page 16-18  Additional file 1: Method S8, Page 8-10 | | |  |
| Model settings | The essential details of the model. For mass-univariate, first level fMRl, these include:  ● Drift model, if not already specified as a dependent variable (e.g. locally linear detrending of data & regressors, as in FSL).  ● Autocorrelation model ([e.g. global](e.g.global) approximate AR (1) in SPM; locally regularized autocorrelation function in FSL).  For mass-univariate second level fMRl these include:  ● Fixed effects (all subjects’ data in one model).  ● Random or mixed-effects model, implemented with:  ○ Ordinary least squares (OLS, aka unweighted summary statistics approach; SPM default, FSL FEAT’s “Simple OLS”).  ○ weighted least squares (i.e. FSL FEAT’s “FLAME 1”), using voxel-wise estimate of between subject variance.  ○ Global weighted least squares (i.e. SPM’s MFX).  With any group (multi-subject) model, indicate any specific variance structure, e.g.  ● Un-equal variance between groups (and if globally pooled, as in SPM).  ● If repeated measures, the specific covariance structure assumed (e.g. compound symmetric, or arbitrary; if globally pooled).  For local-multivariate report:  ● The number of voxels in the local model.  ● Local model used (e.g. Canonical Correlation Analysis) with any constraints (e.g. positive weights only). | Y | Methods, page 16-18  Additional file 1: Method S8, Page 8-10 | | |  |
| Inference: Contrast/effect | ● Specification of the precise effect tested, often as a linear contrast of  parameters in a model. When possible, define these in terms of the task or stimulus conditions instead of psychological concepts (See *Task*  *Specification* in *Experimental Design Reporting*).  ● Provide tables/figures on main effects (e.g. in supplement), not just  differences or interactions. For example, an inference on a difference of  two fMRI conditions, A_B, doesn’t indicate if both A & B induced positive  changes; likewise, to fully interpret an interaction requires knowledge of the main effects.  ● Indicate any use of any omnibus ANOVA tests.  ● All contrasts explored as part of the research should be fully described in the methods section, whether or not they are considered in the results.  ● If performing a two-sided test via two one-sided tests, double the one-sided p-values to convert them into two-sided p-values. For example, if looking at both a contrast [_1 1] and [1 _1] together, each with cluster-forming  threshold p=0.001, double the FWE cluster p-values from each contrast to obtain two-sided inferences. | Y | Methods, page 16-18  Additional file 1: Method S8, Page 8-10 | | |  |
| Inference: Search region | ● Whole brain or “small volume”; carefully describe any small volume correction used for each contrast.  ● If a small-volume correction mask is defined anatomically, provide named anatomical regions from a publicly available ROI atlas.  ● If small-volume correction mask is functionally defined, clearly describe the functional task and identify any risk of circularity.  ● All small-volume corrections should be fully described in the methods section, not just mentioned in passing in the results. | Y | Methods, page 16-18  Additional file 1: Method S8, Page 8-10 | | |  |
| Inference: Statistic type | Typically one of:  ● Voxel-wise (aka peak-wise in SPM).  ● Cluster-wise.  ○ Cluster size.  ○ Cluster mass.  ○ Threshold-free Cluster Enhancement (TFCE). For cluster size or mass, report:  ● Cluster-forming threshold.  For all cluster-wise methods, report:  ● Neighborhood size used to form clusters (e.g. 6, 18 or 26). For TFCE, report:  ● Use of non-default TFCE parameters. | Y | Methods, page 16-18  Additional file 1: Method S8, Page 8-10 | | |  |
| Inference: P-value computation | Report if anything but standard parametric inference used to obtain (uncorrected) P-values. If nonparametric method was used, report method (e.g. permutation or bootstrap) and number of permutations/samples used. | Y | Additional file 1: Method S8, Page 8-10 | | |  |
| Inference: Multiple testing correction | For mass-univariate, specify the type of correction and how it is obtained, especially if not the typical usage. Usually one of:  ● Familywise Error.  ○ Random Field Theory (typical).  ○ Permutation.  ○ Monte Carlo.  ○ Bonferroni.  ● False Discovery Rate.  ○ Benjamini & Hochberg FDR (typical).  ○ Positive FDR.  ○ Local FDR.  ○ Cluster-level FDR.  ● None/Uncorrected.  If permutation or Monte Carlo, report the number of permutations/samples. If Monte Carlo, note the brain mask and smoothness used, and how smoothness was estimated. | Y | Additional file 1: Method S8, Page 8-10 | | |  |
| **Functional connectivity** |  |  |  | | | |
| Confound adjustment & filtering | Report:  ● Method for detecting movement artifacts, movement-related variation, and remediation (e.g. ‘scrubbing’, ‘despiking’, etc).  ● Use of global signal regression, exact type of global signal used and how it was computed.  ● Whether a high_ or low-pass temporal filtering is applied to data, and at which point in the analysis pipeline. Note, any temporal regression model using filtered data should have it’s regressors likewise filtered. | Y | Additional file 1: Methods S3, page 5, paragraph 5 | | | |
| Multivariate method:  Independent Component Analysis | Report:  ● Algorithm to estimate components.  ● Number of components (if fixed), or algorithm for estimating number of components.  ● If used, method to synthesize multiple runs.  ● Sorting method of IC’s, if any.  ● Detailed description of how components were chosen for further analysis. | Y | N/A (No ICA analysis was conducted) | | | |
| Dependent variable definition | For seed-based analyses report:  ● Definition of the seed region(s).  ● Rationale for choosing these regions.  For region-based analyses report:  ● Number of ROIs.  ● How the ROI’s are defined (e.g. citable anatomical atlas; auxiliary fMRI experiments); note if ROIs overlap.  ● Assignment of signals to regions (i.e. how a time series is obtained from each region, e.g. averaging or first singular vector)  ● Note if considering only bilateral (L+R) merged regions.  ● Note if considering only interhemispheric homotopic connectivity. | Y | Methods, page 9, paragraph 2 | | | |
| Functional connectivity measure/ model | Report:  ● Measure of dependence used, e.g. Pearson’s (full) correlation, partial correlation, mutual information, etc; also specify:  ○ Use of Fisher’s Z-transform (Yes/No) and, if standardised, effective N is used to compute standard error (to account for any filtering  operations on the data).  ○ Estimator used for partial correlation.  ○ Estimator used for mutual information.  ● Regression model used to remove confounding effects (Pearson or partial correlation). | Y | Additional file 1: Methods S3, page 6, paragraph 2 | |  |  |
| Effectivity connectivity | Report:  ● Model.  ● Algorithm used to fit model.  ● If per-subject model, method used to generalize inferences to population.  ● Itemize models considered, and method used for model comparison. | Y | N/A (No effectivity connectivity analysis was conducted) | |  |  |
| Graph analysis | Report the ‘dependent variable’ and ‘functional connectivity measure’ used (see above).  Specify either:  ● Weighted graph analysis or,  ● Binarized graph analysis is used, clarifying the method used for  thresholding (e.g. a 10% density threshold, or a statistically-defined  threshold); consider the sensitivity of your findings to the particular choice of threshold used.  Itemise the graph summaries used (e.g. clustering coefficient, efficiency, etc),  whether these are global or per-node/per-edge summaries. ln particular with fMRI or EEG, clarify if measures applied to individual subject networks or group networks. | Y | N/A (No Graph analysis was conducted) | |  |  |
| **Multivariate modelling & predictive analysis** |  |  | N/A (No Multivariate modelling and predictive analysis were conducted) | |  |  |
| Independent variables | Specify:  ● Variable type (discrete or continuous).  ● Class proportions in classification settings.  ● Variable dimension.  o For whole-brain prediction, this is a voxel count.  o For searchlight analyses, the exact number of voxels in the search region, not just a radius.  o Provide dimension before and after any feature selection and/or dimension reduction.  If available, report on population stratification:  ● Information on how the target values relate to the population (e.g. male/female frequency or age distribution by group).  ● Specify how this is taken into account in the predictive model. | Y | N/A |  |  |  |
| Features extraction and dimension reduction | Specify the use of any:  ● Feature transformation.  ● Feature selection.  ● Dimension reduction.  When these techniques are data-driven, specify the procedures used to learn the parameters involved. | Y | N/A |  |  |  |
| Model | For traditional multivariate analyses, report:  ● Type of model, e.g. MANOVA.  ● Assumptions made on the covariance structure, e.g. independence, or a common arbitrary covariance between groups.  ● Statistic used to assess significance, e.g. Wilk’s lambda, Hotelling-Lawley trace, etc.  For predictive models, report:  ● Type of model, e.g. Linear discriminant analysis, support vector machines, logistic regression, etc.  ● For kernel-based methods (i.e. SVM) report type of kernel used, type and number of parameters needed to be estimated. | Y | N/A |  |  |  |
| Learning method | Report:  ● Figure-of-merit optimised.  ● Fitting method.  ● Parameter settings, those fixed and those estimated; specify how fixed parameter values were chosen.  ● How the convergence of the learning method is monitored. | Y | N/A |  |  |  |
| Training procedure | Describe:  ● Pipeline structure applied uniformly to all cases (e.g. that could be independently applied to a new case).  ● Method for hyper-parameter setting.  ● Data splitting (cross validation). | Y | N/A | |  |  |
| Evaluation metrics: Discrete response | Describe the evaluation metrics that are to be computed. Always compute:  ● Accuracy.  ● If group sizes unequal, balanced (or average) accuracy.  When there are only 2 classes, and one can be labeled “positive”:  ● Precision (1 _ false discovery rate).  ● Recall (sensitivity).  ● False positive rate (1_specificity).  ● F1 (incorporates both precision and recall).  ● Receiver operating characteristic (ROC) curves, e.g. summarised by area under the curve (AUC); AUC for only high specificity (e.g. false positive rates no greater than 10%) are also useful.  When there are 3 or more classes:  ● Report the confusion matrix. | Y | N/A | |  |  |
| Evaluation metrics: Continuous response | “Prediction R^2^”, the percentage of variance explained by prediction, computed as one minus the ratio of prediction sum-of-squares to total sum-of-squares. (Note this *is not* the squared correlation coefficient between true and predicted values). | Y | N/A | |  |  |
| Evaluation metrics:  Representational similarity analysis | Report the Kendall Tau statistic for each candidate model considered. | Y | N/A | |  |  |
| Evaluation metrics: Significance | When possible use formal test to obtain P-value to assess whether evaluation metric is “significant” or consistent with noise. | Y | N/A |  |  |  |
| Fit interpretation | Procedure used to interpret the fit of the classifier, identifying the relative importance of the features (e.g. the weight vector in linear discriminant). | N | N/A |  |  |  |

**Table D.5. Results Reporting**

| **Aspect** | **Notes/Ontology** | **Mandatory** | **Location** |
| --- | --- | --- | --- |
| **Mass univariate analysis** |  |  |  |
| Effects tested | Provide a complete list of tested and omitted effects. | Y | Results, page 25-26, paragraphs 1 |
| Extracted data | ● Define how voxels/elements were selected; if region is based on the same data, clarify how circularity was accounted for.  ● For any summary reported, give units. Ideally these are as interpretable as possible (e.g. percent change).  ● If reporting R2 (coefficient of determination) clarify how nuisance variability is considered. For instance, in task fMRI the vast majority of variance is  explained by slow temporal drift, and R2 values for an effect of interest will be vastly different if computed with or without counting drift in the total  variance. | Y | Results, page 25-26, paragraphs 1 |
| Tables of coordinates | Provide one table of coordinates including:  ● Contrast / effect to which it refers.  ● XYZ coordinate (with coordinate system, MNI, Talairach, noted in caption; also clarify whether peak or center-of-mass location).  ● Anatomical region (in caption or body text, describe source of labels, e.g. subjective, atlas, etc).  ● P-value forming basis of inference (e.g. voxel-wise FWE corrected P; or cluster-wise FDR corrected P).  ● T/Z/F statistic (with degrees of freedom in table caption)  ● In caption, state whether coordinates are from whole brain, or from a specific constrained volume.  ● If cluster-wise inference is used, the cluster size. Report in mm3 or, if in voxels, be explicit about the size of voxels. If a cluster statistic other than size is used (e.g. mass) it should be listed as well.  ● In caption or body text, note criterion for peak per cluster reporting; e.g. “one peak per cluster listed”, or “up to 3 per cluster that are at least 8mm apart” (SPM default), etc. | Y | Results, page 25-26, paragraphs 1 |
| Thresholded maps | For each effect, provide images of maps of significant regions, ensuring that each caption describes:  ● Type of inference and the correction method, as well as form of any  sub-volume corrections applied when computing corrected significance.  ● Include color bars; when presenting multiple maps in a figure, use a common color bar to ensure the results are comparable. | N | N/A |
| Unthresholded maps | Share, via supplementary material or repository:  ● Unthresholded statistic maps.  ● Optionally, the thresholded statistic maps.  ● Optionally, the effect size map (e.g. % BOLD change, % GM change). | Y | Additional file1: Fig S1, Page 47 |
| Extracted data | State whether data extracted from an ROI (e.g. to compute an effect size) is defined based on independent data, as otherwise it is susceptible to bias.  If ROIs are circularly defined, best not to provide any statistical summary (i.e. P-values, R^2^, etc). | Y | Results, page 25-26, paragraphs 1 |
| Spatial features | Report the  ● Size of the analysis volume in voxels, mm.  ● Spatial smoothness of noise (e.g. FWHM) and Resel count (if using Random Field Theory). | Y | Results, page 25-26, paragraphs 1 |
| **Functional connectivity** |  |  |  |
| ICA analyses | Report the total number of components (especially when estimated from the data and not fixed). Report the number of these analyzed and the reason for their selection. | Y | N/A |
| Graph analyses: Null hypothesis tested | For graph-based methods, carefully state what is the null hypothesis of the test and how the statistic distribution under the null is computed. | Y | N/A |
| **Multivariate modelling & predictiveanalysis** |  |  |  |
| Optimised evaluation metrics | Report the values obtained for the evaluation metrics chosen (see Evaluation Metrics, above), as well as any P-values to justify above-chance performance. | Y | N/A |

**Table D.6. Data Sharing**

| **Aspect** | **Notes** | | **Mandatory** | **Location** |
| --- | --- | --- | --- | --- |
| **Reporting a data sharing resource** |  | |  |  |
| Material shared | List types of images and non-imaging data provided.  Report on the completeness of the data (e.g., number of subjects where all types of imaging, demographic, and behavioral data is available). | | Y | Declarations, page 36, paragraph 4 |
| URL, access information | Provide:  ● Stable URL or DOI.  ● Specific instructions on how to gain access. Specifically mention whether application must be vetted for particular intended research use ([e.g. to](e.g.to)  preclude multiple users investigating the same question), or whether a research collaboration must be established.  ● Cost of access. | | Y | Declarations, page 37, paragraph 1 |
| Ethics compliance | Confirm that the ethics board of the host institution generating the data approves the sharing of the data made available.  Clarify any constraints on uses of shared data, for example, whether users downloading the data also need ethics approval from their own institution. | Y | | Declarations, page 36, paragraph 4 |
| Documentation | Provide URL to documentation, and specify its scope (e.g. worked examples, white papers, etc). | N | | Declarations, page 37, paragraph 1 |
| Data format | Report the format of the image data shared, e.g. DICOM, MINC, NIFTI, etc. | Y | | Declarations, page 36, paragraph 4 |
| Ontologies | Data organization structures, including Data Dictionaries and Schemas. Is the software using an established ontology? | N | | Declarations, page 37, paragraph 2 |
| Visualization | Availability of in-resource visualization of the imaging or non-imaging data. | N | | Declarations, page 36, paragraph 4 |
| De-identification | How, if at all, data are de-identified. | N | | N/A |
| Provenance and history | Availability of detailed provenance of preprocessing and analysis of shared data. | N | | Declarations, page 36, paragraph 4 |
| Interoperability | Ability of a repository to work in a multi-database environment, availability of API’s and ability to connect to analysis pipelines. | N | | N/A |
| Querying | Mechanisms available for constructing queries on the repository (e.g. SQL, SPARQL). | N | | N/A |
| Versioning | How users can check version of downloaded data and compare it to the current version at a later time. | N | | N/A |

**Table D.7. Reproducibility**

| **Aspect** | **Notes/Ontology** | **Mandatory** | **Location** |
| --- | --- | --- | --- |
| **Documentation** |  |  |  |
| Tools used | Tool names, versions, and URLs. | Y | Methods, page 15, paragraph 2 and paragraph 3; page 15, paragraph 2  Additional file 1: Methods S3, page 5, paragraph 5; page 7, paragraph 3; page 9, paragraph 2; page 10, paragraph 1 |
| Infrastructure | Machine CPU model, operating system version, any use of parallelization. | Y | Additional file 1: Methods S3, page 6, paragraph 2 |
| Workflow | Use of a workflow system, its version and URL. | N | N/A |
| Provenance trace | State whether detailed provenance information is available. | N | N/A |
| Literate program implementing results | Provide a URL linking to the relevant resource; for example, an ipython notebook implementing key analyses. | N | N/A |
| English language version | As the scientific lingua franca, documentation should be provided in English in addition to any other languages. | N | Full manuscript and all additional files |
| **Archiving** |  |  |  |
| Tools availability | Note if tools are publically available. | N | Methods, page 15, paragraph 2 and paragraph 3; page 15, paragraph 2  Additional file 1: Methods S3, page 5, paragraph 5; page 7, paragraph 3; page 9, paragraph 2; page 10, paragraph 1 |
| Virtual appliances | Note if a virtual environment to facilitate a repeated analysis is available. | N | N/A |
| **Citation** |  |  |  |
| Data | Provide permanent identifier if possible. | N | Data Availability statement |
| Workflow | Provide permanent identifier if possible. | N | N/A |
